# Microglia-Secreted Factors Enhance Dopaminergic Differentiation of Tissue- and iPSC-Derived Human Neural Stem Cells

**DOI:** 10.1016/j.stemcr.2020.12.011

**Published:** 2021-01-21

**Authors:** Sissel Ida Schmidt, Helle Bogetofte, Louise Ritter, Jette Bach Agergaard, Ditte Hammerich, Amina Arslanagic Kabiljagic, Agnieszka Wlodarczyk, Silvia Garcia Lopez, Mia Dahl Sørensen, Mie Lærkegård Jørgensen, Justyna Okarmus, Alberto Martínez Serrano, Bjarne Winther Kristensen, Kristine Freude, Trevor Owens, Morten Meyer

**Affiliations:** 1 Department of Neurobiology Research, Institute of Molecular Medicine, University of Southern Denmark, Odense, DK; 2 Department of Molecular Biology and Center of Molecular Biology Severo Ochoa, University Autonoma Madrid-C.S.I.C., Madrid, ES; 3 Department of Pathology, Odense University Hospital, Odense, DK; 4 Department of Neurology, Odense University Hospital, Odense, DK; 5 Faculty of Health and Medical Sciences, Department of Veterinary and Animal Sciences, Section for Pathobiological Sciences, University of Copenhagen, Copenhagen, DK; 6 BRIDGE – Brain Research Inter-Disciplinary Guided Excellence, Department of Clinical Research, University of Southern Denmark, Odense, DK

**Keywords:** co-culture, microglia, NSCs, TNFα, IGF1, IL-1β, dopamine, neuron-microglia interaction, secretome analysis, Parkinson’s disease

## Abstract

Microglia have recently been established as key regulators of brain development. However, their role in neuronal subtype specification remains largely unknown. Using three different co-culture setups, we show that microglia-secreted factors enhance dopaminergic differentiation of somatic and induced pluripotent stem cell-derived human neural stem cells (NSCs). The effect was consistent across different NSC and microglial cell lines and was independent of prior microglial activation, although restricted to microglia of embryonic origin. We provide evidence that the effect is mediated through reduced cell proliferation and decreased apoptosis and necrosis orchestrated in a sequential manner during the differentiation process. tumor necrosis factor alpha, interleukin-1β, and insulinlike growth factor 1 are identified as key mediators of the effect and shown to directly increase dopaminergic differentiation of human NSCs. These findings demonstrate a positive effect of microglia on dopaminergic neurogenesis and may provide new insights into inductive and protective factors that can stimulate *in vitro* derivation of dopaminergic neurons.

## Highlights

• Differentiating NSCs in co-culture with microglia enhance dopaminergic differentiation
• The effect is restricted to microglia of embryonic origin
• Microglial TNFα, IL-1β, and IGF1 are key mediators of the effect

## Graphical Abstract

**Figure:**
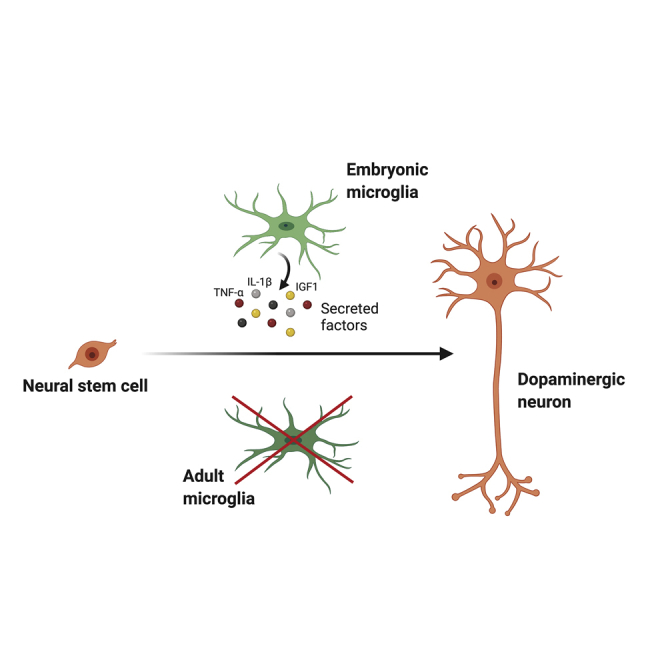

## Teaser

In this article, Schmidt and colleagues show that differentiating human NSCs in co-culture with microglia enhance dopaminergic differentiation. The effect is consistent across different NSC and microglial cell lines but restricted to microglia of embryonic origin. TNFα, IL-1β, and IGF1 are identified as key mediators of the effect, providing new insights into factors stimulating dopaminergic differentiation.

## Introduction

Neurogenesis is a complex process comprising several steps that require regulation by the microenvironment. Neural stem cell (NSC) proliferation and differentiation, migration of neuroblasts to their appropriate location, survival of immature and mature neurons, and construction of synaptic connectivity thus all rely on extrinsic cues (Ekdahl et al., 2009).

Microglia are immune cells in the central nervous system (CNS) that originate from hematopoietic progenitors of the yolk sac and start to colonize the developing brain as early as the fourth gestational week (Menassa and Gomez-Nicola, 2018). As microglia are present in the brain before the emergence of neurons and other glia, they may play an important role in providing a proper microenvironment for embryonic neurogenesis. This hypothesis is supported by studies showing that colony-stimulating factor 1 receptor knockout mice, which lack microglia, display abnormal brain development (Elmore et al., 2014), and that microglia can induce developmental apoptosis and thereby regulate the size of the neural precursor pool (Cunningham et al., 2013; Marin-Teva et al., 2004; Tronnes et al., 2016; Wakselman et al., 2008). In addition, microglia have been reported to modulate synaptogenesis through local synthesis of neurotrophic factors (Miyamoto et al., 2016; Parkhurst et al., 2013), participate in synaptic pruning (Paolicelli et al., 2011; Schafer et al., 2012), and guide axonal outgrowth (Pont-Lezica et al., 2014; Squarzoni et al., 2014).

Studies suggest that the neurogenic effect of microglia is dependent on their activation state and is mediated by their cytokine release (Shigemoto-Mogami et al., 2014; Wang et al., 2007). Following pro-inflammatory activation (e.g., by stimulation with lipopolysaccharide [LPS]), microglia secrete pro-inflammatory cytokines including tumor necrosis factor alpha (TNFα), interleukin (IL)-1β, interferon-γ (IFN-γ), and nitric oxide and reduce their release of neurotrophic factors. In contrast, stimulation with, e.g., IL-4 causes microglia to secrete anti-inflammatory cytokines including IL-4 and IL-10, and neurotrophic factors such as brain-derived neurotrophic factor (BDNF), glial cell line-derived neurotrophic factor (GDNF), and insulin growth factor 1 (IGF1) (Franco and Fernandez-Suarez, 2015; Polazzi and Monti, 2010). While the pro-inflammatory activated microglial cells generally appear to inhibit differentiation and proliferation of NSCs and cause aberrant migration of newly formed neurons in the adult rat hippocampus (Ekdahl et al., 2003; Monje et al., 2003; Yang et al., 2010), the anti-inflammatory activated microglia have a neuroprotective role and can increase neurogenesis and oligodendrogenesis of NSCs (Butovsky et al., 2006; Yuan et al., 2017). However, it remains unclear how the effects mediated by pro- and anti-inflammatory secreted molecules is influenced by NSC-microglia interactions (Mosher et al., 2012; Su et al., 2014).

Microglia are widely, but not uniformly, distributed throughout the CNS. In the rodent brain, they are present in higher density in many brain regions containing dopaminergic neurons or dopaminergic projections, such as the substantia nigra, striatum, hippocampus, and the olfactory system, compared with surrounding structures (De Biase et al., 2017; Lawson et al., 1990). This suggests that the microenvironment offered by the microglia might be important for dopaminergic neuronal development in particular, providing potential new insights into inductive factors for dopaminergic differentiation.

To address the role of microglia and their activation state in dopaminergic neurogenesis, we investigated whether microglia-secreted factors enhanced dopaminergic differentiation of somatic and induced pluripotent stem cell (iPSC)-derived human NSCs *in vitro*. We found that, independent of the microglial cell type and NSC lines, co-culturing NSCs with microglia during differentiation increased the content of dopaminergic neurons in the cultures. This effect was limited to microglia of embryonic origin and was not influenced by prior microglial activation. Microglial-secreted TNFα, IL-1β, and IGF1 were identified as key mediators, and recombinant TNFα, IL-1β, and IGF1 were shown to directly increase dopaminergic differentiation.

## Results

### Microglia-Secreted Factors Enhance Dopaminergic Differentiation of Human NSCs

**Figure 1:**
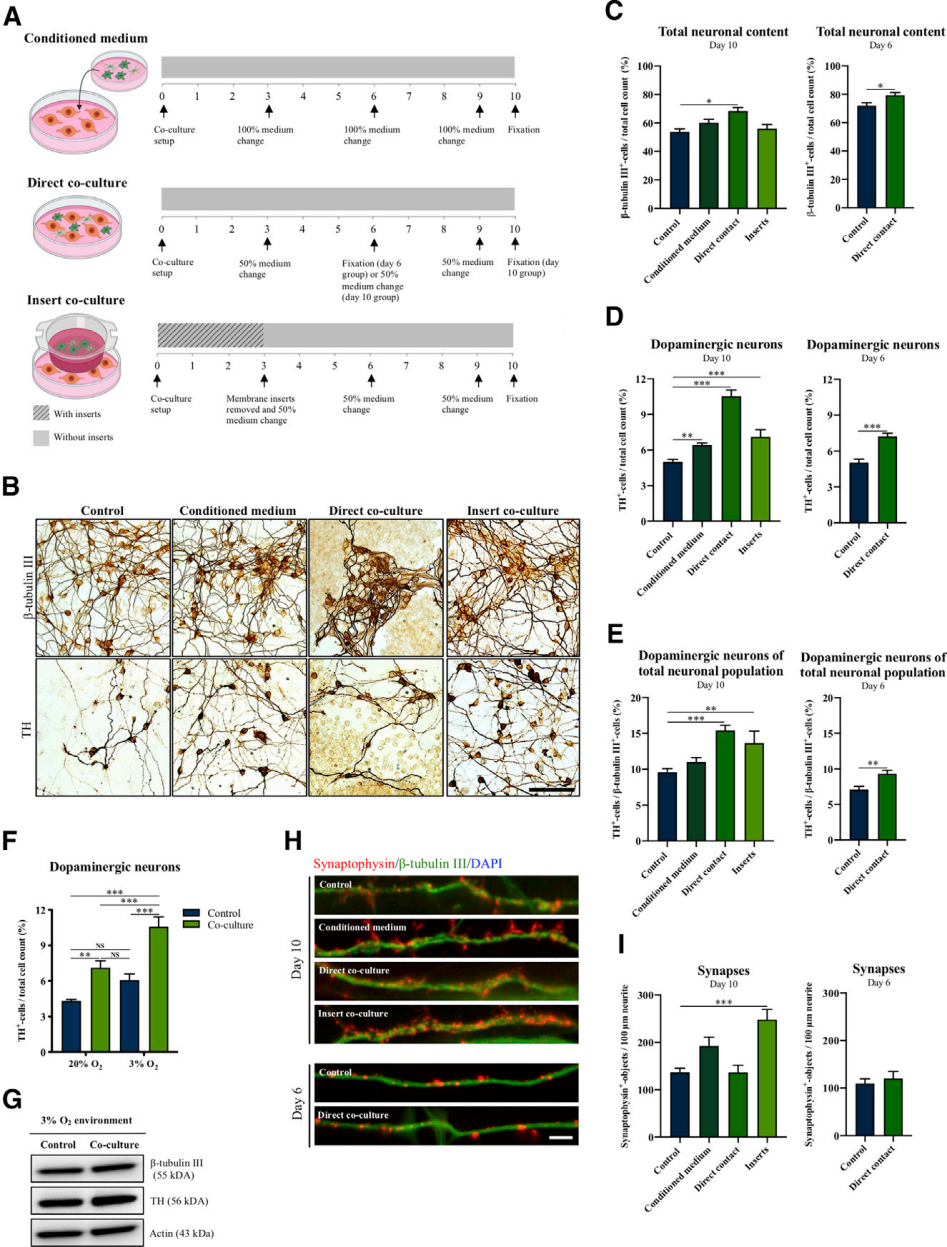
Increased Dopaminergic Differentiation of NSCs Using Different Microglia Co-culture Setups (A) hVM1-Bcl-X_L_ NSCs were either exposed to BV2 microglia-conditioned medium, directly co-cultured with BV2 microglia (physical contact), or indirectly co-cultured (separated by semi-porous membrane inserts). (B–E) Immunocytochemical staining and quantification of differentiated neurons for (C) β-tubulin III^+^ neurons/total cell count, (D) TH^+^ neurons/total cell count, and (E) the number of TH^+^ neurons/β-tubulin III^+^ neurons. Scale bar: 100 μm. One-way ANOVA, Dunnett's multiple comparison test with reference to control. Day 10: control, n = 23, N = 6; conditioned medium, n = 16, N = 4; direct contact, n = 6, N = 2; inserts, n = 13, N = 4. Day 6: control, n = 14, N = 4; direct contact, n = 14, N = 4. (F and G) TH^+^ neurons/total cell count and Western blotting for β-tubulin III and TH in differentiated hVM1-Bcl-X_L_ NSCs cultures after combining the indirect co-culture setup with physiological O_2_tension (3% O_2_). Two-way ANOVA, Tukey's multiple comparison test. Control, n = 14, N = 4; co-culture, n = 13, N = 4. (H and I) Synaptophysin^+^ objects/100 μm neurite. Scalebar: 5 μm. One-way ANOVA, Dunnett's multiple comparison test with reference to control. Day 10: control, n = 6, N = 2; conditioned medium, n = 6, N = 2; direct contact, n = 4, N = 2, inserts, n = 6, N = 2. Day 6: control, n = 4, N = 2; direct contact, n = 4, N = 2. Mean ± SEM. ^∗^p < 0.05, ^∗∗^p < 0.01, ^∗∗∗^p < 0.001, NS = not significant. See Figures S1 for additional data.

Three different co-culture settings were applied to investigate the effect of microglia on dopaminergic differentiation of human NSCs. During 10 days of spontaneous differentiation, hVM1-Bcl-X_L_ NSCs (human ventral mesencephalic NSCs) were either grown in BV2 microglia-conditioned medium or directly (physical contact) or indirectly (separated by semi-porous membrane inserts) co-cultured with BV2 microglia (Figure 1A). Upon differentiation, the number of cells positive for the pan-neuronal marker β-tubulin III had increased for the direct co-culture group (Figures 1B and 1C), whereas the number of dopaminergic tyrosine hydroxylase-positive (TH^+^) neurons was significantly increased in all groups compared with control (Figure 1D and 1B). As BV2 microglia were overgrowing the direct co-culture (Figure S1A), an earlier time point (day 6) was also examined, showing a similar effect on TH^+^ neuronal content. When calculating the percentage of TH^+^ neurons relative to the total neuronal population (TH/β-tubulin III ratio), only the direct and indirect co-culture setups revealed a significant increase in the yield of TH^+^ neurons (Figure 1E). No difference in the total cell numbers was observed (Figure S1B).

The high density of microglia in the direct co-culture group caused the neurons to cluster together at day 10 (Figures 1B and S1A). We therefore investigated whether this growth pattern influenced the neuronal connectivity in the cultures and found an increased synaptic density (synaptophysin^+^ objects/100 μm neurite) and a more differentiated morphology for neurons in the indirect co-culture group compared with control (Figures 1H and 1I). Verification of the data using other NSC and microglia cell lines revealed that the positive effects on TH^+^ neuronal yield and synaptic density were consistent across all groups compared with control (Figures S1C–S1F).

We have previously shown that exposing NSCs during differentiation to physiological oxygen (O_2_) tension (3%–5%) rather than atmospheric O_2_ tension (20%), which is the standard for most cell culture incubators, enhances the dopaminergic differentiation outcome (Krabbe et al., 2014). Differentiating hVM1-Bcl-X_L_ NSCs at physiological O_2_ tension in co-culture with BV2 microglia using membrane inserts further increased the yield of TH^+^ neurons (Figures 1F and 1G).

Since increased numbers of TH^+^ cells were detected at all co-culture conditions, the effect of microglia was most likely mediated by secreted factors and unlikely to be dependent on physical cell-cell interaction. Based on cell morphology and the yield of TH^+^ neurons, we therefore concluded that the optimal co-culture differentiation setup would be achieved using semi-porous membrane inserts combined with physiological O_2_ tension, which was applied for the remaining experiments.

**Figure 2:**
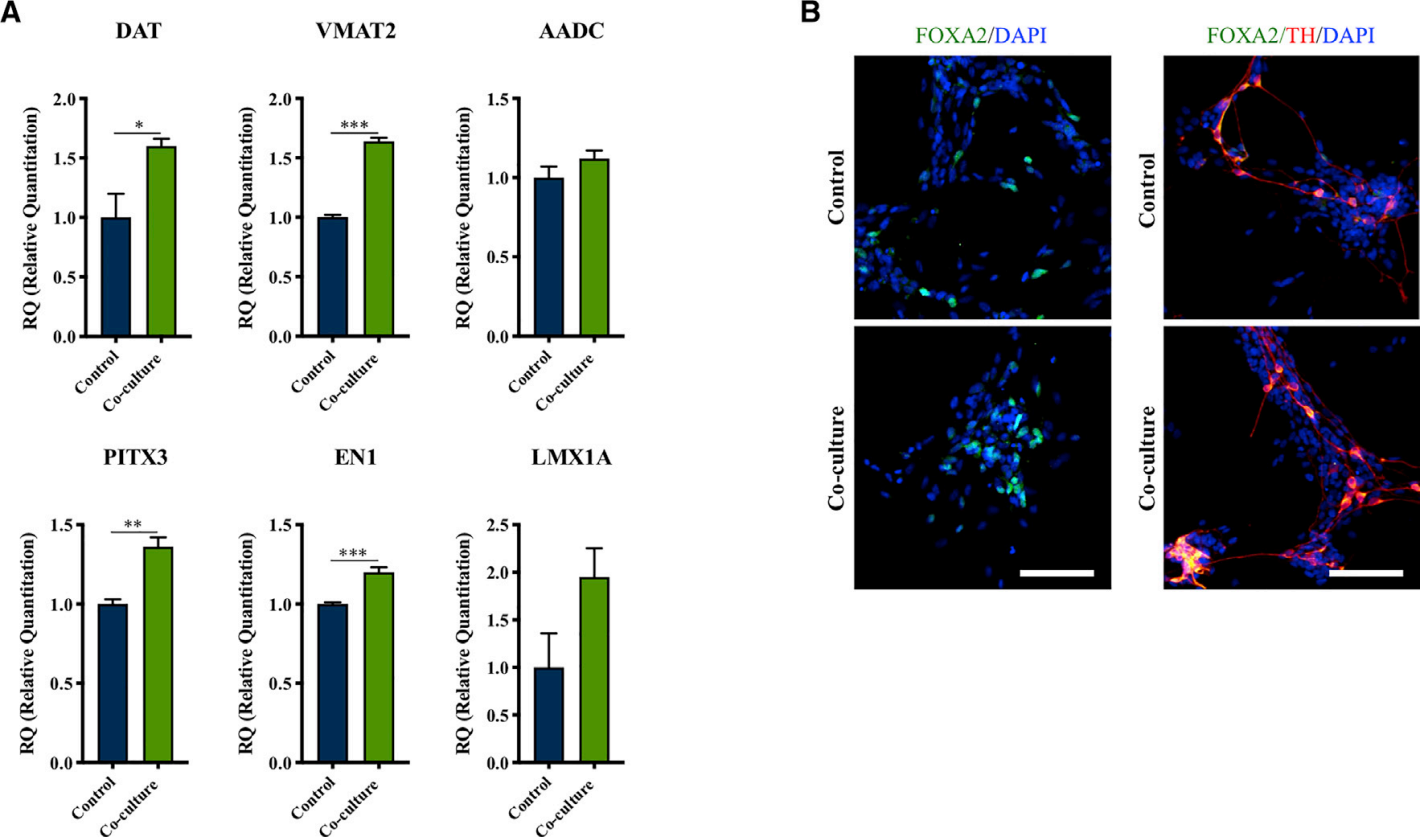
Microglia Co-culture Increased Expression of Dopaminergic and Midbrain-Specific Markers (A) qRT-PCR data for the plasma membrane *DAT*, *VMAT2*, the midbrain-specific transcription factors *PITX3*, the homeobox protein *EN1*, *AADC*, and the midbrain dopaminergic *LMX1A* upon co-culture differentiation of hVM1-Bcl-X_L_ NSCs and BV2 microglia. Student's t test. Control, n = 4, N = 2; co-culture, n = 4, N = 2. Mean ± SEM. ^∗^p < 0.05, ^∗∗^p < 0.01, ^∗∗∗^p < 0.001. (B) Expression of the floorplate marker FOXA2 in TH^+^ neurons. Scale bar: 100 μm.

To further characterize the population of TH^+^ neurons, qRT-PCR was performed for additional dopaminergic neuronal markers (Figure 2A). Co-culture with microglia significantly increased the expression of the plasma membrane dopamine transporter (*DAT*), the vesicular monoamine transporter 2 (*VMAT2*), and the midbrain-specific transcription factors pituitary homeobox 3 (*PITX3*) and homeobox protein engrailed-1 (*EN1*) genes. The expression of the aromatic amino acid decarboxylase (*AADC*) and the midbrain dopaminergic LIM homeobox transcription factor 1-alpha (*LMX1A*) was not significantly changed. Midbrain characteristics of the TH^+^ neurons were also confirmed by FOXA2 immunostaining (Figure 2B).

### Enhanced Dopaminergic Differentiation Is Consistently Found When Combining Different Microglial Cells and NSCs

**Figure 3:**
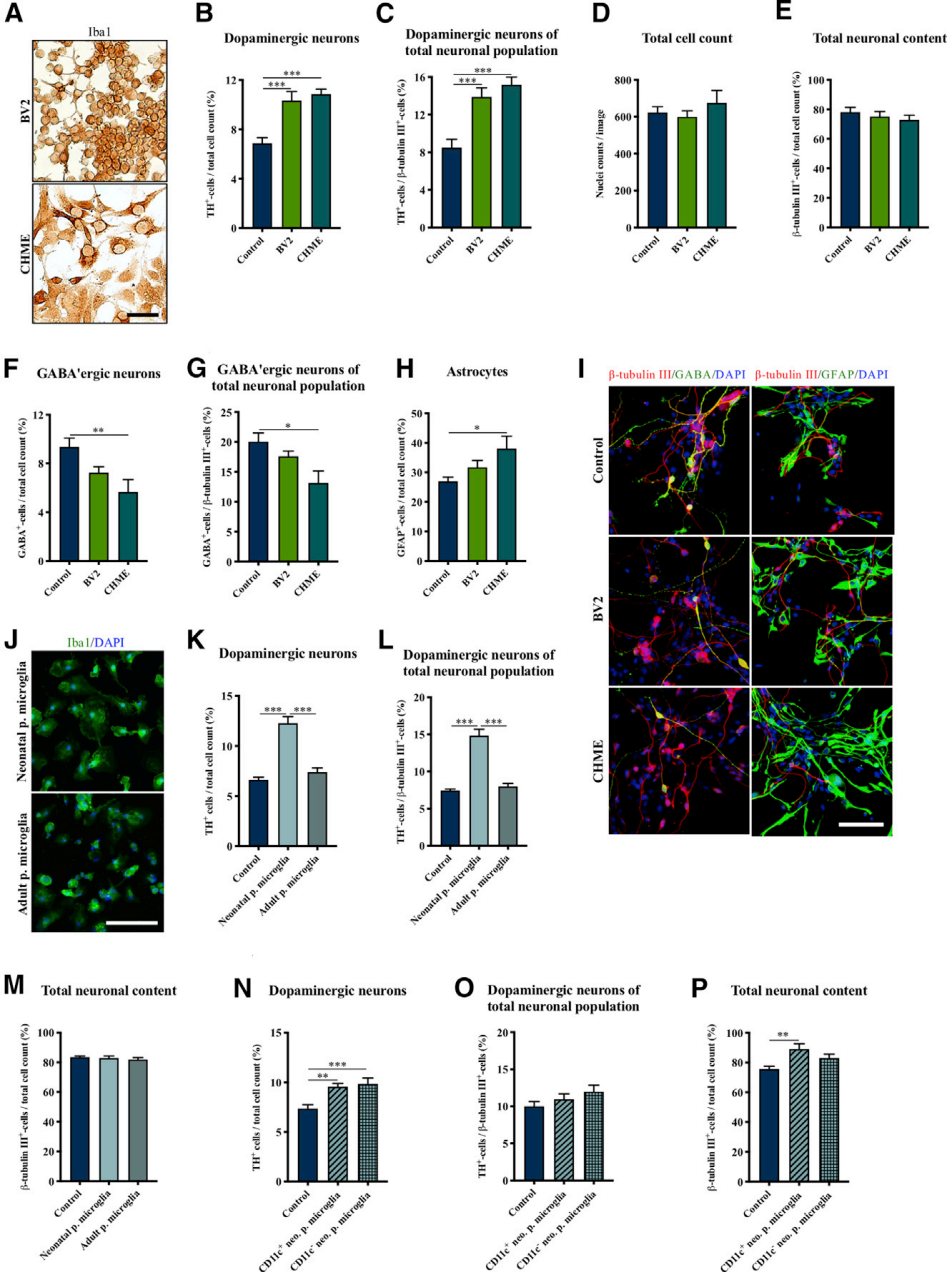
Consistent Positive Effect on Dopaminergic Differentiation Across Different Microglia Cell Lines (A) Immunocytochemical staining of murine BV2 and human CHME microglia cells for the microglial marker Iba1. Scale bar: 50 μm. (B–E) Immunocytochemical staining and quantification of hVM1-Bcl-X_L_ NSC-derived neurons after CHME and BV2 microglia co-culture differentiation for (B) TH^+^ neurons/total cell count, (C) TH^+^ neurons/β-tubulin III^+^ neurons, (D) total cell count, and (E) β-tubulin III^+^ neurons/total cell count. Control, n = 21, N = 8; BV2, n = 15, N = 5; CHME, n = 12, N = 5. (F–I) Immunofluorescence staining and quantification of (F and I) GABA^+^ neurons/total cell count, (G) GABA^+^ neurons/β-tubulin III^+^ neurons, and (H and I) GFAP^+^-astrocytes/total cell count. Scale bar: 100 μm. Control, n = 6, N = 2; BV2, n = 6, N = 2; CHME, n = 6, N = 2. (J) Immunofluorescence staining of neonatal and adult primary microglia (p. microglia) for the microglial marker Iba1. Scale bar: 100 μm. (K–M) Quantification of (K) TH^+^ neurons/total cell count; control, n = 18, n = 4; neonatal p. microglia, n = 9, N = 2; adult p. microglia, n = 12, N = 4, (L) β-tubulin III^+^ neurons/total cell count, and (M) TH^+^ neurons/β-tubulin III^+^ neurons; control, n = 14, n = 4; neonatal p. microglia, n = 9, N = 2; adult p. microglia, n = 8, N = 2, after differentiation of hVM1-Bcl-X_L_ NSCs in co-culture with murine neonatal or adult p. microglia. (N–P) Quantification of (N) TH^+^ neurons/total cell count, (O) β-tubulin III^+^ neurons/total cell count, and (P) TH^+^ neurons/β-tubulin III^+^ neurons after differentiating hVM1-Bcl-X_L_ NSCs co-cultured with murine CD11c^+^ or CD11c^−^ neonatal p. microglia. Control, n = 17, N = 6; CD11c^+^*neo*. p. microglia, n = 10, N = 4, CD11c^−^*neo*. p. microglia, n = 10, N = 4. Mean ± SEM. One-way ANOVA, Dunnett's multiple comparison test with reference to control. ^∗^p < 0.05, ^∗∗^p < 0.01, ^∗∗∗^p < 0.001. See Figures S2–S4 for additional data.

We next investigated how consistent the positive effect of microglia on dopaminergic differentiation was for different microglial and NSC lines. Human hVM1-Bcl-X_L_ cells were spontaneously differentiated for 10 days in co-culture with either murine BV2 microglia or human CHME microglia cells (Figure 3A). Both microglial cell lines were able to significantly enhance the number of TH^+^ neurons as evaluated both relative to total cell count and the TH/β-tubulin III ratio (Figures 3B, 3C, and S3A). This was verified using another NSC line (Figure S2). Surprisingly, the increased numbers of TH^+^ neurons were not reflected in either the total cell count or the total neuronal cell count, which revealed no difference for either of the co-culture groups compared with control (Figures 3D and 3E). Taken together, these results suggest that, while the effect on the TH^+^ neuronal content was consistently increased across the microglial cell lines, it was not mediated by an overall increased neurogenesis. To investigate if this was due to a shift in the cellular composition of the culture, we characterized the content of other cell types known to be present in the midbrain (Morello and Partanen, 2015). This revealed that the increase in the content of TH^+^ neurons might be at the expense of GABAergic differentiation as the number of GABA^+^ neurons was decreased for both co-culture groups (BV2 and CHME microglia) compared with control; however, it was only significant for the CHME group (Figures 3F, 3G, and 3I). Concerning other neuronal subpopulations, the cultures contained very few glutamatergic (<1%) and serotonergic (<1%) neurons (Figure S3B), and no obvious difference between the groups was seen. In contrast, the numbers of glial fibrillary acidic protein (GFAP)^+^ astrocytes were increased for both groups, but again were only significant for the CHME microglial cell line (Figures 3H and 3I). The GFAP staining was found to co-localize with the markers S100β and vimentin (Figure S3C), confirming the presence of astrocytes.

The positive effect of co-culturing hVM1-Bcl-X_L_ NSCs with microglia during dopaminergic differentiation was further verified using primary microglia from either P3-P5 pups or adult mice (Figure 3J). Interestingly, only neonatal primary microglia increased the TH^+^ neuronal yield, while the total neuronal content and total cell numbers were unchanged for both types of microglia (Figures 3K–3M and S3D). It was recently discovered that a CD11c^+^ neonatal microglia subtype plays a key role in myelination and neurogenesis in the developing mouse brain through release of IGF1 (Wlodarczyk et al., 2017). We therefore next investigated if a CD11c^+^ subtype of neonatal primary microglia was responsible for the positive effect of microglia on dopaminergic differentiation. Interestingly, both the CD11c^+^ and CD11c^−^ neonatal primary microglia were able to increase the number of TH^+^ neurons, whereas the TH/β-tubulin III ratio was unchanged compared with control (Figures 3N–3O). Contrary to the effect of the BV2 and CHME microglia cell lines, CD11c^+^ neonatal primary microglia increased the total neuronal yield, but no difference was observed for the total cell number (Figures 3P and S3E). A comparative secretome analysis revealed that both BV2, CHME, adult and neonatal primary microglia secreted all of the investigated cytokines, but considerably higher levels were detected for adult and neonatal primary microglia. Only IGF1 was not detected in conditioned culture medium from adult primary microglia (Figure S4).

**Figure 4:**
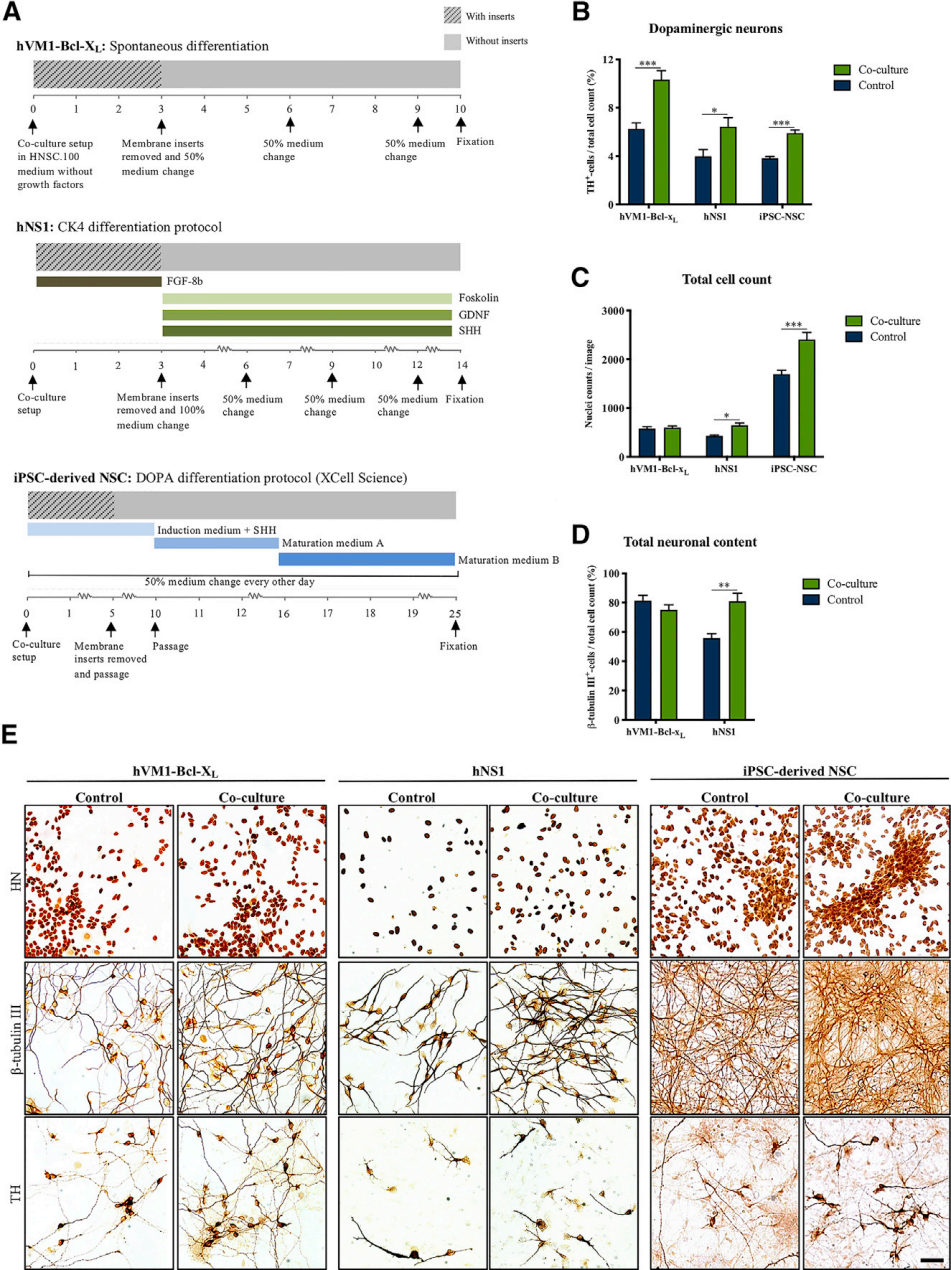
Microglia Co-culture Consistently Increases Dopaminergic Differentiation of Different NSC Lines (A) Graphical representation of the three different dopaminergic differentiation protocols applied for the hVM1-Bcl-X_L_, hNS1, and iPSC-derived NSCs and the period of BV2 microglia co-culturing. (B–E) Effect of BV2 microglia co-culture differentiation on all three NSC lines as measured by (B and E) TH^+^ neurons/total cell count (C and E) total cell count and (D and E) β-tubulin III^+^ neurons/total cell count. Scale bar: 50 μm. Multiple t test, Holm-Sidak's multiple comparison test. HVM1-Bcl-X_L_: control, n = 15, N = 5; co-culture, n = 15, N = 5. hNS1: control, n = 8, N = 2; co-culture, n = 8, N = 2. iPSC-NSCs: control, n = 17, N = 4; co-culture, n = 15, N = 4. Mean ± SEM. ^∗^p < 0.05, ^∗∗^p < 0.01, ^∗∗∗^p < 0.001.

We then investigated if the dopaminergic neurogenic effect of microglia was consistent across different NSC lines. The human forebrain NSC line hNS1, the iPSC-derived NSC line XCL1, and the hVM1-Bcl-X_L_ cell line were all differentiated in co-culture with BV2 microglia, resulting in consistently enhanced TH^+^ neuronal content (Figures 4A, 4B, and 4E). Interestingly, the total cell number following differentiation was markedly increased for the hNS1 and iPSC-NSC co-culture setups, but not for hVM1-Bcl-X_L_ (Figures 4C and 4E). Similarly, the number of β-tubulin III^+^ cells in the hNS1 cultures was significantly increased for the co-culture group, which contradicted the results obtained for hVM1-Bcl-X_L_ cells (Figures 4D and 4E). Due to the iPSC-NSC cultures being highly confluent, β-tubulin III cell quantification was not feasible. However, iPSC-NSC cultures immunostained for β-tubulin III displayed a markedly denser staining pattern in the co-culture group (Figure 4E). Table S1 summarizes the cell counts for all co-culture combinations of NSC and microglia cell lines presented here.

### Microglial Co-culture Enhances Dopaminergic Neuronal Neurite Length, Inhibits Proliferation, and Improves Survival of Differentiating NSCs

**Figure 5:**
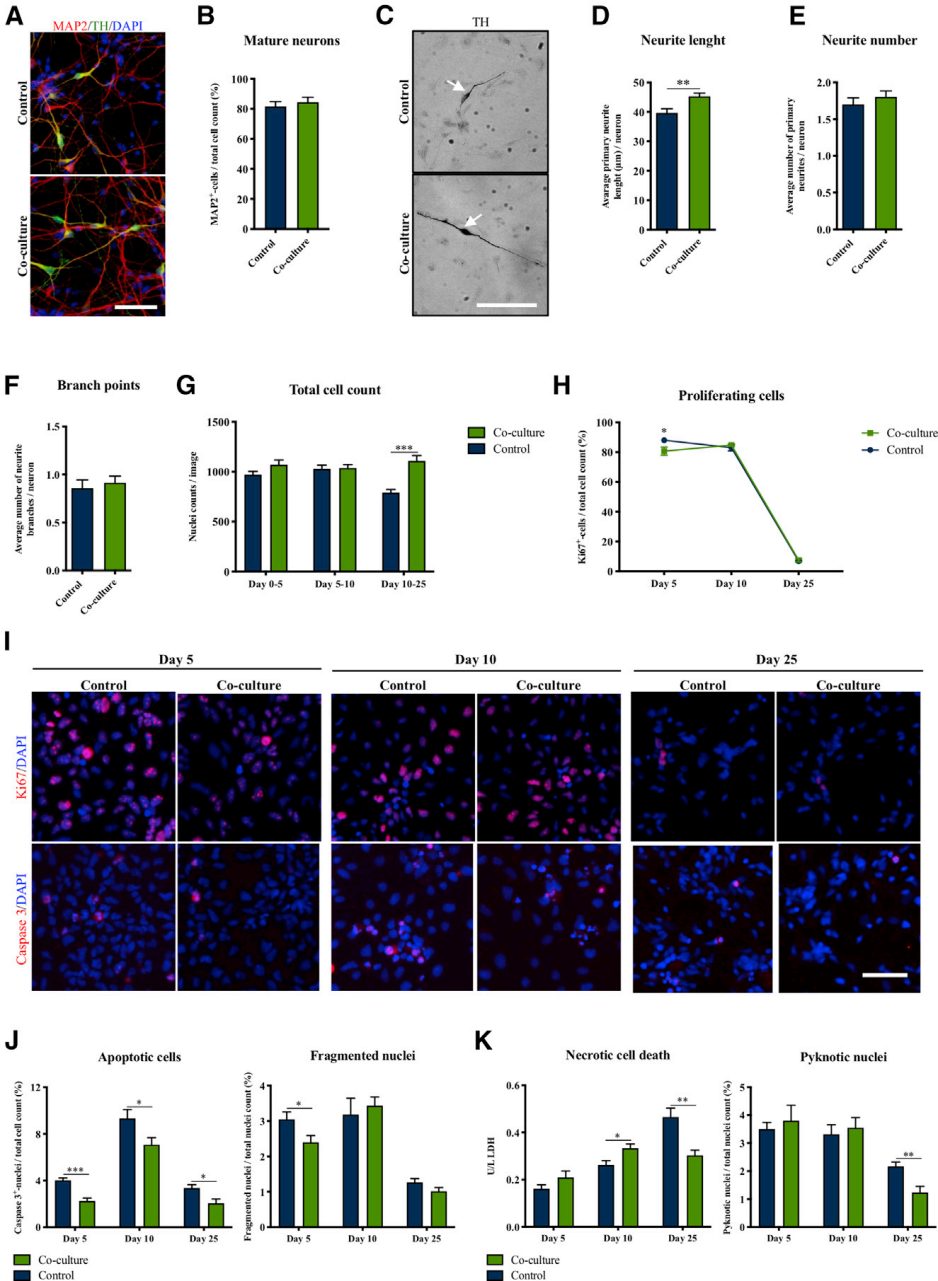
Microglia Co-culture Affects Neurite Outgrowth, Proliferation, and Cell Death of NSCs (A and B) Immunofluorescence staining and quantification of iPSC-NSC/BV2 co-culture and control differentiated cultures for the mature pan neuronal marker microtubule-associated protein (MAP2)/total cell count. Scale bar: 50 μm. Student's t test. Control, n = 7, N = 3; co-culture, n = 7, N = 3. (C–F) Morphological analysis of TH^+^ neurons in hNS1 NSC/BV2 co-culture assessing (D) neurite length, (E) neurite number, and (F) number of neurite branch points. Scale bar: 100 μm. Student’s t test. Control, n = 8, N = 2; co-culture, n = 8, N = 2. (G) Total cell counts at days 5, 10, and 25 during differentiation of iPSC-NSCs in co-culture with BV2. Multiple t test, Holm-Sidak's multiple comparison test. Days 0–5 and 5–10: control, n = 6, N = 2; co-culture, n = 6, N = 2. Days 10–25: control, n = 19, N = 4; co-culture, n = 14, N = 4. (H–J) (H and I) Immunofluorescence staining and quantification of iPSC-NSCs for the proliferation marker Ki67 at days 5, 10, and 25 during differentiation. Scale bar: 50 μm. Multiple t tests, Holm-Sidak's multiple comparison test. Day 5 and 10: control, n = 6, N = 2; co-culture, n = 6, N = 2. Day 25: control, n = 9, N = 4; co-culture, n = 7, N = 4. (I and J) Apoptotic cell death in iPSC-NSC/BV2 co-culture at days 5, 10, and 25 during differentiation measured by immunofluorescence staining and quantification of cleaved caspase 3^+^ nuclei/total cell count and fragmented nuclei/total cell count. Multiple t test, Holm-Sidak's multiple comparison test. Days 5, 10, and 25: control, n = 6, N = 2; co-culture, n = 6, N = 2. (K) Necrotic cell death in iPSC-NSC/BV2 co-culture at days 5, 10, and 25 during differentiation measured by lactate dehydrogenase (LDH) release to the medium and pyknotic nuclei/total cell count. Multiple t test, Holm-Sidak's multiple comparison test. Day 5: control, n = 6, N = 2; co-culture; n = 6, N = 2. Days 10 and 25: control, n = 12, N = 4; co-culture, n = 12, N = 4. Mean ± SEM. ^∗^p < 0.05, ^∗∗^p < 0.01, ^∗∗∗^p < 0.001.

To investigate the effect of microglia on neuronal maturation, we quantified in 25-day-old iPSC-NSC-derived cultures the number of microtubule-associated protein 2 (MAP2)^+^ mature neurons, which was unaffected by BV2 co-culture (Figures 5A and 5B). However, morphological analysis in 14-day-old hNS1 cultures showed that the mature TH^+^ neurons in the co-culture group had significantly increased neurite length compared with controls, suggesting a more mature dopaminergic neuronal phenotype (Figures 5C and 5D). Neurite numbers and numbers of neurite branches per neurite were unchanged (Figures 5E and 5F). Collectively, these data suggest that the microglia do not directly influence the number of mature neurons generated, but rather the maturation of the dopaminergic neuronal morphology.

As the total cell count upon co-culture differentiation was markedly increased for both the hNS1 and iPSC-NSCs, but not for the hVM1-Bcl-X_L_ cells (Figure 4C), we investigated when this increase in total cell count appeared during differentiation of the iPSC-NSCs in co-culture with BV2 cells. At days 5 and 10, total cell counts did not differ between controls and co-cultures, but, at day 25, co-cultures displayed significantly increased cell numbers (Figure 5G). As this could either be due to increased proliferation and/or decreased cell death in the cultures, these parameters were investigated during the differentiation of the iPSC-NSCs. The proliferation marker Ki67 was slightly, but significantly, reduced in the co-cultures at day 5, but unchanged at later time points (Figures 5H and 5I). Immunostaining for the apoptotic marker cleaved caspase 3 combined with morphological analysis of fragmented DAPI^+^ nuclei revealed a significant reduction of apoptotic cell death in the co-cultures at all time points investigated (Figures 5I and 5J). Investigation of necrotic cell death by measuring lactate dehydrogenase release and by counting pyknotic DAPI^+^ nuclei revealed that necrotic cell death was significantly reduced in the co-culture group at day 25 (Figure 5K).

### Dopaminergic Neurogenic Effect of Microglia Is Independent of The Microglial Activation State and Possibly Mediated by Release of TNFα, IL-1β, and IGF1

**Figure 6:**
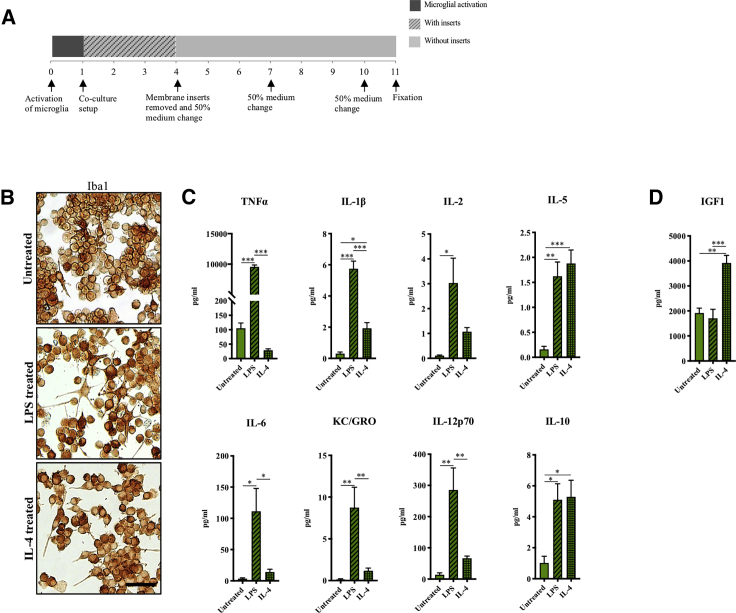
Microglial Cells Treated with LPS and IL-4 show Pro- and Anti-inflammatory Activation Profiles, Respectively (A) BV2 microglia were activated with 100 ng/mL LPS or 20 ng/mL IL-4 for 24 h prior to co-culture with hVM1-Bcl-X_L_ cells. (B) Immunocytochemical staining of activated BV2 microglia for the microglial marker Iba1. Scale bar: 100 μm. (C) Cytokine profiling of microglia medium after 24 h of activation. Untreated, n = 5, N = 3; LPS, n = 7, N = 4; IL-4, n = 7, N = 4. (D) ELISA for IGF1 of microglia medium at 24 h post activation. Untreated, n = 4, N = 3; LPS, n = 6, N = 4; IL-4, n = 6, N = 4. One-way ANOVA, Tukey's multiple comparison test. Mean ± SEM. ^∗^p < 0.05, ^∗∗^p < 0.01, ^∗∗∗^p < 0.001. See Figures S5 and S7 for additional data.

To investigate if the neurogenic effect of microglia depended on their activation state (Butovsky et al., 2006; Yuan et al., 2017), BV2 microglia were either stimulated with 100 ng/mL LPS or 20 ng/mL IL-4 for 24 h prior to co-culture with hVM1-Bcl-X_L_ cells (Figure 6A) (Kobayashi et al., 2013). Microglia activation was confirmed by cytokine profiling of conditioned culture medium collected 24 h after stimulation (for dose-response data see Figure S5A). Compared with untreated BV2 microglia, LPS-stimulated BV2 microglia displayed a more ramified morphology (Figure 6B) and significantly increased secretion of the pro-inflammatory cytokines TNFα, IL-1β, IL-2, IL-5, IL-6, KC/GROα, IL-12p70, but also IL-10 (Figure 6C). IL-4-stimulated microglia displayed a bipolar morphology (Figure 6B) and decreased their secretion of TNFα compared with untreated BV2 microglia while increasing their secretion of IL-10 and IL-5. IL-4-stimulated microglia were also found to increase their secretion of IL-1β, IL-2, IL-6, KC/GROα, and IL-12p70, but to a significantly lesser extent than the LPS-stimulated microglia (Figure 6C). Importantly, IL-4-stimulated BV2 microglial cells markedly increased their secretion of IGF1 compared with untreated BV2 and LPS-activated BV2 microglia (Figure 6D). Altogether, these secretome analyses confirmed a pro-inflammatory activation upon LPS stimulation of the BV2 microglia and a more anti-inflammatory activation upon IL-4 stimulation.

**Figure 7:**
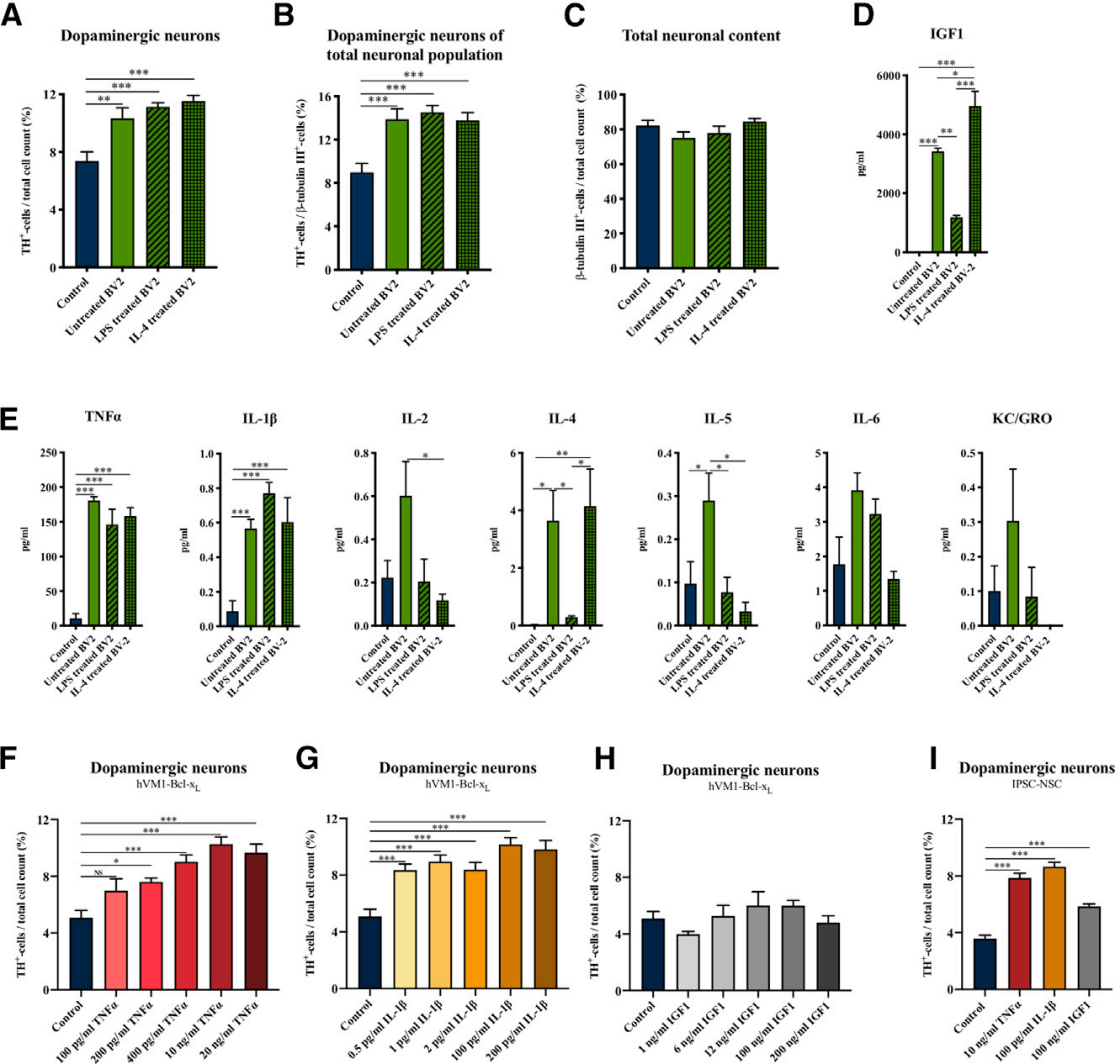
Increased Dopaminergic Differentiation of NSCs Independent on Prior Microglial Activation and Identification of TNFα, IL-1β, and IGF1 as Potential Mediators (A–C) Immunocytochemical staining and quantification of hVM1-Bcl-X_L_ NSC-derived neurons after co-culture differentiation with pro- and anti-inflammatory activated BV2 microglia for (A) TH^+^ neurons/total cell count, (B) TH^+^ neurons/β-tubulin III^+^ neurons, and (C) β-tubulin III^+^ neurons/total cell count. One-way ANOVA, Dunnett's multiple comparison test with reference to control. Control, n = 20, N = 7; untreated BV2, n = 15, N = 5; LPS-treated BV2, n = 10, N = 4; IL-4 treated BV2, n = 12, N = 5. (D) IGF1 ELISA; all groups, n = 4, N = 2, and (E) cytokine profiling; control, n = 6, N = 3; untreated BV2, LPS-treated BV2, and IL-4 treated BV2, n = 4, N = 2, of co-culture medium collected at day 3 of differentiation. One-way ANOVA, Tukey's multiple comparison test. (F–H) Effect on TH^+^ neurons/total cell count of direct addition of recombinant human (F) TNFα, (G) IL-1β, and (H) IGF1 to hVM1-Bcl-X_L_ NSCs during differentiation. One-way ANOVA, Dunnett's multiple comparison test with reference to control. All groups, n = 6, N = 2. (I) Most efficient concentrations of recombinant TNFα, IL-1β, and IGF1 tested on iPSC-NSCs. One-way ANOVA, Dunnett's multiple comparison test with reference to control. All groups, n = 6, N = 2. Mean ± SEM. ^∗^p < 0.05, ^∗∗^p < 0.01, ^∗∗∗^p < 0.001, NS = not significant. See Figures S6 and S7 for additional data.

Subsequent evaluation of the activated BV2 microglia's effect on the TH^+^ neuronal yield following NSC differentiation revealed that neither pro- nor anti-inflammatory activation had changed the positive effect of the BV2 microglia (Figures 7A–7C, S5B, and S5C). This could suggest that co-culturing with NSCs had altered the microglial secretome independently of the prior microglial activation state. Cytokine profiling of the conditioned medium, collected after 3 days of co-culture, showed that the microglial secretome was altered upon co-culturing (Figures 7E and S6), but the secretome of carry-on cultures also revealed that the degree of activation was reduced over time (Figure S7). Medium from all three co-culture groups contained similar levels of TNFα (145–180 pg/mL) and IL-1β (0.5–0.8 pg/mL), which were significantly increased compared with the control group. IL-2, IL-4, IL-5, IL-6, and KC/GROα were also detected in the culture medium but varied between the groups. As the effect on the dopaminergic differentiation of the NSCs was similar for untreated and stimulated BV2 microglia, TNFα and IL-1β could be the potential mediators. When the same medium was analyzed for IGF1 using ELISA, we saw a high content (1,000–5,000 pg/mL) for all co-culture groups compared with a non-detectable level in conditioned medium from controls (Figure 7D). Although the IGF1 content in the media from the LPS-activated BV2 microglia co-culture group was significantly lower than that of the untreated BV2 and IL-4-treated BV2 co-culture groups, the presence of high levels of IGF1 in all co-culture groups suggested that IGF1 was also a potential mediator.

We next investigated if direct addition of recombinant TNFα, IL-1β and IGF1 could increase the dopaminergic differentiation. Three concentrations matching the levels detected in the conditioned culture medium and two concentrations reflecting existing literature (Doherty, 2007; Ling et al., 1998; Supeno et al., 2013) were added to differentiating hVM1-Bcl-X_L_ cells. Both TNFα and IL-1β increased the yield of TH^+^ neurons, whereas IGF1 had no significant effect (Figures 7F–7H). The most efficient concentrations of TNFα, IL-1β, and IGF1 were also tested on the iPSC-derived NSC line XCL1, which confirmed the positive effect of TNFα and IL-1β, but also IGF1 was found to increase the yield of TH^+^ neurons from this cell line (Figure 7I).

## Discussion

In the present study, we have shown that co-culture of differentiating human NSCs with microglia stimulates dopaminergic differentiation, an effect that was consistent across different types of microglial and NSC lines (Table S1).

The direct co-culture, allowing physical cell-cell contact, was impaired by microglial overgrowth, so, based on both cell morphology and yield of TH^+^ neurons, the indirect co-culture using semi-porous membrane inserts was found to be optimal. As these membranes allow only diffusible, non-physical interactions between the NSCs and microglia, secreted factors were likely responsible for the observed increase in dopaminergic content. The positive effect of microglia co-culture on dopaminergic differentiation was supported by qRT-PCR, revealing increased expression of general dopaminergic markers and midbrain-specific transcription factors, and co-expression of the floorplate marker FOXA2 in TH^+^ neurons. Previous studies have confirmed that all the applied NSC lines (hVM1-Bcl-X_L_, hNS,1 and iPSC-NSC [XCL1]) generate TH^+^ neurons of the midbrain dopaminergic phenotype (Bogetofte et al., 2019; Liste et al., 2004; Okarmus et al., 2020; Seiz et al., 2012).

Although the positive effect on dopaminergic differentiation was consistent across the investigated microglia and NSC lines, the effect on the general neurogenesis varied. For the hVM1-Bcl-X_L_ cells, CD11c^+^ neonatal primary microglia increased the percentage of β-tubulin III^+^-neurons, whereas no difference was found for unsorted neonatal primary microglia, and BV2 and CHME microglia. The increased dopaminergic differentiation caused by CHME and BV2 microglia was possibly at the expense of GABAergic differentiation, resulting in an unchanged general neurogenesis. This could suggest a difference between microglia cell lines in their effect on the differentiation of other neuronal subtypes than the dopaminergic, but the general neurogenic effect was also found to vary between the NSC lines. Differentiating hNS1 in co-culture with BV2 increased the percentage of β-tubulin III^+^-neurons, and a similar trend was observed for the iPSC-NSCs (not quantified). This difference might be explained by overexpression of the anti-apoptotic protein Bcl-X_L_ in the hVM1-Bcl-X_L_ line as the effect of the microglia was partly apoptosis mediated.

Primary microglia isolated from adult mice failed to increase the dopaminergic differentiation of the NSCs. Adult and neonatal microglia have recently been shown to have very different gene expression profiles (Matcovitch-Natan et al., 2016; Wlodarczyk et al., 2017), and, as both CHME and BV2 microglia cell lines have been produced from neonatal microglia (Blasi et al., 1990; Janabi et al., 1995), our findings suggest that the dopaminergic neurogenic effect of microglia is restricted to microglia of embryonic origin. Our secretome comparison between the different microglia cells revealed secretion of all investigated cytokines; however, adult and primary microglia generally secreted higher levels than BV2 and CHME. It can be speculated whether this is due to the isolation and sorting of the primary microglia prior to *in vitro* culturing, as such procedures have been shown to induce transient activation of microglia (Bohlen et al., 2019).

As microglia have been reported to play a variety of roles during brain development (Menassa and Gomez-Nicola, 2018), we investigated their effect on neuronal maturation, morphological development, proliferation, and cell death during dopaminergic differentiation of the NSCs. Although no apparent effect on the number of MAP2^+^ mature neurons was observed, the dopaminergic neurons displayed a more mature morphology with increased neurite length. This is in line with previous studies on human fetal tissue where microglia were associated with axonal tracts during development (Monier et al., 2007; Verney et al., 2010) and microglial deficiency caused aberrant dopaminergic axonal outgrowth in mouse embryos (Squarzoni et al., 2014), suggesting a role of microglia in axonal development. A slight decline in proliferation and reduced apoptosis was observed at the beginning of differentiation in the presence of the microglia, suggesting neurotrophic support from microglia. Apoptotic and necrotic cell death was reduced at the end of differentiation when microglia were no longer present. As differentiating the NSCs in co-culture with microglia also increased the number of astrocytes, the reduced cell death at the end of differentiation may have been due to microglia-astrocyte crosstalk leading to increased neurotrophic support. The total cell count upon co-culture differentiation was markedly increased for both the hNS1 and iPSC-NSC cell lines but not for the hVM1-Bcl-X_L_ cells. It is possible that the overexpression of the anti-apoptotic protein Bcl-X_L_ in this cell line may have masked the anti-apoptotic effect of the microglia secretome.

Although previous studies have reported a selective neurogenic effect of anti-inflammatory activated microglia (Butovsky et al., 2006; Yuan et al., 2017), we found that the dopaminergic neurogenic effect on NSCs was independent of prior microglial activation as both untreated, pro-, and anti-inflammatory activated BV2 microglia increased the yields of dopaminergic neurons. In line with this, cytokine profiling of the conditioned co-culture media at day 3 of differentiation showed very different profiles for untreated, LPS-treated, and IL-4-treated BV2 groups compared with their cytokine release prior to co-culture, suggesting that NSC-microglia crosstalk changes the microglial secretome toward a neurogenic profile independent of the prior activation state. This was supported by secretome analysis comparing co-cultures with microglia carry-on monocultures at day 3, which revealed higher cytokine levels in medium from co-cultures, especially of TNFα and IL-1β, that were unaffected by prior activation. It was, however, also shown from the carry-on microglia that the degree of activation was reduced over time. In comparison, a previous study showed that iPSC-derived microglial progenitors activated with LPS displayed a more anti-inflammatory cytokine response when co-cultured with iPSC-derived cortical neuronal progenitors compared with corresponding monocultures (Haenseler et al., 2017). Furthermore, activation of embryonic microglia has previously been reported not to alter the increased survival of embryonic dopaminergic neurons observed in a primary co-culture cell model (Zietlow et al., 1999). NSC-microglia crosstalk is therefore most likely playing a role.

TNFα, IL-1β, and IGF1 were the only factors found at similar levels in the media for all co-culture groups, suggesting that they might be responsible for the increased dopaminergic neurogenesis. Embryonic microglia are known to secrete cytokines, including IL-1β and TNFα (Deverman and Patterson, 2009; Giulian et al., 1988; Munoz-Fernandez and Fresno, 1998), and IGF1 (Wlodarczyk et al., 2017). IL-1β is a recognized mitogen for astrocytes (Giulian et al., 1988; Wang et al., 2007) and has been found to increase astrogliogenesis while decreasing neurogenesis of hippocampal neural progenitor cells (NPCs) (Chen et al., 2013; Koo and Duman, 2008; Wang et al., 2007; Zunszain et al., 2012), but recombinant IL-1β has also been shown to enhance differentiation of mesencephalic NPCs into dopaminergic neurons (Ling et al., 1998). The IL-1β concentration detected in our conditioned co-culture medium was very low (0.5–0.8 pg/mL), but treatment of differentiating hVM1-Bcl-X_L_ NSCs with both low and high concentrations of recombinant human IL-1β increased the yield of dopaminergic neurons.

IGF1 exerts pleiotropic effects during embryogenesis and in adulthood, including NSC proliferation, astrogliogenesis, and neuronal survival, differentiation, and maturation (Nieto-Estevez et al., 2016). Microglia-induced neurogenesis in the early postnatal subventricular zone is not mediated through the release of IGF1 from microglia, however, but rather their release of IL-1β, IL-6, TNFα, and IFN-γ (Shigemoto-Mogami et al., 2014). To our knowledge, no previous studies have linked IGF1 with selective dopaminergic neurogenesis, and we found that CD11c^+^ and CD11c^−^ neonatal primary microglia increased the yields of dopaminergic neurons to a similar extent, although CD11C^+^ microglia express much higher levels of IGF1 (Wlodarczyk et al., 2017). This suggests that IGF1 alone is not responsible for the increased dopaminergic differentiation observed in our experiments. Direct addition of recombinant human IGF1 to differentiating hVM1-Bcl-X_L_ did not affect the resulting number of dopaminergic neurons.

TNFα is an important regulator of developmental apoptosis and synaptogenesis (Mosser et al., 2017), but it is also implicated in the pathogenesis of neurodegenerative conditions such as Parkinson disease (Mogi et al., 1994, 2000). It has previously been reported that exposure of E12.5 mouse ventral mesencephalic dopaminergic neurons to TNFα increased the number of dopaminergic neurons, while TNFα-treated cultures from E14-E16 mice displayed a decreased number of dopaminergic neurons due to apoptotic cell death (Doherty, 2007). This suggests that developing ventral mesencephalic dopaminergic neurons switch their response to TNFα from neurotrophic to neurotoxic as they mature. In accordance with this hypothesis, treatment of hVM1-Bcl-X_L_ NSCs with recombinant human TNFα was found to stimulate dopaminergic neuronal differentiation. Testing the effect of recombinant TNFα, IL-1β, and IGF-1 on another NSC line, iPSC-derived NSCs, revealed that all factors had a positive effect on dopaminergic differentiation, suggesting that IGF1, under certain circumstances, may also exert a positive role.

This is the first study to comprehensively demonstrate that co-culturing of human NSCs with microglia during differentiation enhances the yield of dopaminergic neurons. The effect was found to be consistent across different NSC lines but was restricted to microglia of embryonic origin. Pre-activation of the microglia did not change the positive effect, and TNFα, IL-1β, and IGF-1 were identified as potential key mediators. We provide evidence that the effect is mediated through reduced proliferation and decreased apoptotic/necrotic cell death taking place in a sequential manner during the differentiation process. These findings indicate an instructive role of microglia on dopaminergic neurogenesis and may provide new insights into potential inductive and protective factors that can improve *in vitro* derivation of human dopaminergic neurons.

## Experimental Procedures

See Supplemental Information for detailed methods.

### Human NSC Lines

Cell isolation, genetic modification, and general characterization of the somatic human NSC lines hVM1-Bcl-X_L_ (ventral mesencephalic NSC line) and hNS1 (forebrain NSC line) are described elsewhere (Courtois et al., 2010; Krabbe et al., 2009, 2014; Villa et al., 2000, 2004, 2009). Propagation and dopaminergic differentiation were performed as previously described (Krabbe et al., 2009, 2014). The human iPSC-derived NSC line (XCL1) was obtained from XCell Science Inc. (Novato, CA) (Swistowski et al., 2010) and propagated according to standard protocols provided by the manufacturer. See Supplemental information for detailed procedures.

### Microglia Cell Lines

Establishments of the mouse microglia cell line BV2 and the human microglia cell line CHME are described elsewhere (Blasi et al., 1990; Janabi et al., 1995). Propagation methods are described in Supplemental information.

Primary microglia were isolated from the CNS of P3-P5 C57BL/6j mice and from adult (8–10 weeks) C57BL/6j mice (Wlodarczyk et al., 2014). Cell isolation and sorting are described in Supplemental information.

### Co-culture Procedures

NSCs were exposed to microglia-secreted factors during differentiation by three different co-culture setups: exposure to BV2 microglia-conditioned media, direct co-culture, or co-culture using semi-porous membrane inserts (Figure 1A).

Microglia-conditioned medium was generated by culturing BV2 microglia in HNSC.100 medium for 3 days and collecting the medium through a 0.22-μm filter (Sarstedt) before mixing 1:1 with new unconditioned medium. hVM1-Bcl-X_L_ cells were seeded in this medium at day 0 of differentiation. One-hundred percent media change was performed every third day using newly prepared conditioned medium mixed in the same way. The control group was differentiated in unconditioned HNSC.100 medium.

For direct co-culture, hVM1-Bcl-X_L_ cells were seeded together with BV2 microglia cells in a 1:50 ratio, and differentiation was performed as described in the Supplemental information. The control group did not contain any microglia cells.

For co-culture using semi-porous membrane inserts (Merck), NSCs were seeded and allowed to attach for half an hour before inserts were placed and microglia seeded on the inserts in a 1:5 ratio relative to the NSCs. Differentiation was performed as described in the Supplemental information, and inserts were removed at day 3 for hVM1-Bcl-X_L_ and hNS1 differentiations and at day 5 for iPSC-NSC differentiation. The control groups received empty inserts.

### Immunocytochemistry, Bioimaging and Western Blotting

Immunocytochemistry, image analysis, and western blotting were performed as described in Supplemental information.

### Microglial Activation and Cytokine Profiling

BV2 microglia were activated with either 100 ng/mL LPS from *Escherichia coli* O111:B4 (Sigma) or 20 ng/mL IL-4 (Peprotech) for 24 h in serum-free RPMI-1640 medium prior to co-culture setup (Kobayashi et al., 2013). Activation was confirmed by cytokine profiling using the V-PLEX Proinflammatoy Panel 1 Mouse kit (MesoScale Discovery) and IGF1 ELISA (Sigma) according to the manufacturers' instructions. The same MesoScale kit and IGF1 ELISA were used to screen the conditioned co-culture media at day 3 of differentiation and for carry-on microglia cultures.

### TNFα, IL-1β, and IGF-1 Treatments

hVM1-Bcl-X_L_ cells and iPSC-NSCs were differentiated with addition of human recombinant TNFα (Sigma), IL-1β (R&D), or IGF-1 (R&D) during the first part of differentiation. Details are provided in Supplemental information.

### Statistical Analysis

Analysis was performed in GraphPad Prism version 6.0 (GraphPad Software, United States) using two-tailed unpaired Student's t test, multiple t test, one-way ANOVA, or two-way ANOVA as appropriate. Data are presented as mean ± SEM, and p values < 0.05 were considered statistically significant.

## Author Contributions

Conceptualization, S.I.S., H.B., and M.M.; Methodology and Investigation, S.I.S., H.B., L.R., J.B.A., D.H., A.A., A.W., S.G.L., M.J., and J.O.; Resources, A.M.S., M.D.S., B.W.K., T.O., and M.M.; Data Curation, S.I.S; Writing – Original Draft, S.I.S., H.B., K.F., and M.M.; Writing – Review & Editing, all authors; Funding Acquisition, T.O., A.M.S. and M.M.

## Supplemental Information

Document S1. Supplemental Experimental Procedures, Figures S1–S7, and Table S1

Document S2. Article plus Supplemental Information

## Footnotes

Supplemental Information can be found online at https://doi.org/10.1016/j.stemcr.2020.12.011.
