# Supplementary material for: Microglia-Secreted Factors Enhance Dopaminergic Differentiation of Tissue- and iPSC-Derived Human Neural Stem Cells
Source: Stem Cell Reports. 2021 Jan 21;16(2):281–94. doi: 10.1016/j.stemcr.2020.12.011 (PMC7878834; doi:10.1016/j.stemcr.2020.12.011)
Supplement: Document S1. Supplemental Experimental Procedures, Figures S1–S7, and Table S1 [file mmc1.pdf]

Stem Cell Reports, Volume *16*

Supplemental Information

Microglia-Secreted Factors Enhance Dopaminergic Differentiation of Tissue- and iPSC-Derived Human Neural Stem Cells

Sissel Ida Schmidt, Helle Bogetofte, Louise Ritter, Jette Bach Agergaard, Ditte Hammerich, Amina Arslanagic Kabiljagic, Agnieszka Wlodarczyk, Silvia Garcia Lopez, Mia Dahl Sørensen, Mie Lærkegård Jørgensen, Justyna Okarmus, Alberto Martínez Serrano, Bjarne Winther Kristensen, Kristine Freude, Trevor Owens, and Morten Meyer

Supplemental Information

Supplemental Table of Contents

Figure S1. Additional data for the different co-culture setups, Related to Figure 1.

Figure S2. Consistent positive effect of BV2 and CHME microglia on dopaminergic differentiation of the iPSC-NSC line XCL-1, Related to Figure 3.

Figure S3. Further characterization of differentiated hVM1-Bcl-X_L/BV2/CHME co-cultures and effects on total cell count for co-culture with different primary microglial cell types, Related to Figure 3.

Figure S4. Secretome comparison between BV2, CHME, adult and neonatal primary microglia, Related to Figure 3.

Figure S5. Dose-response data for BV2 microglial activation with LPS and IL-4, Related to Figure 5 and 6.

Figure S6. Secretome comparison of BV2 microglia in co-culture vs. carry-on monocultures, Related to Figure 7.

Figure S7. Secretome analysis of activated BV2 carry-on cultures, Related to Figure 6 and 7.

Table S1. Result overview for the different co-culture combinations of NSC and microglial cell lines.

Supplemental Experimental Procedures

Supplemental References

Figure S1

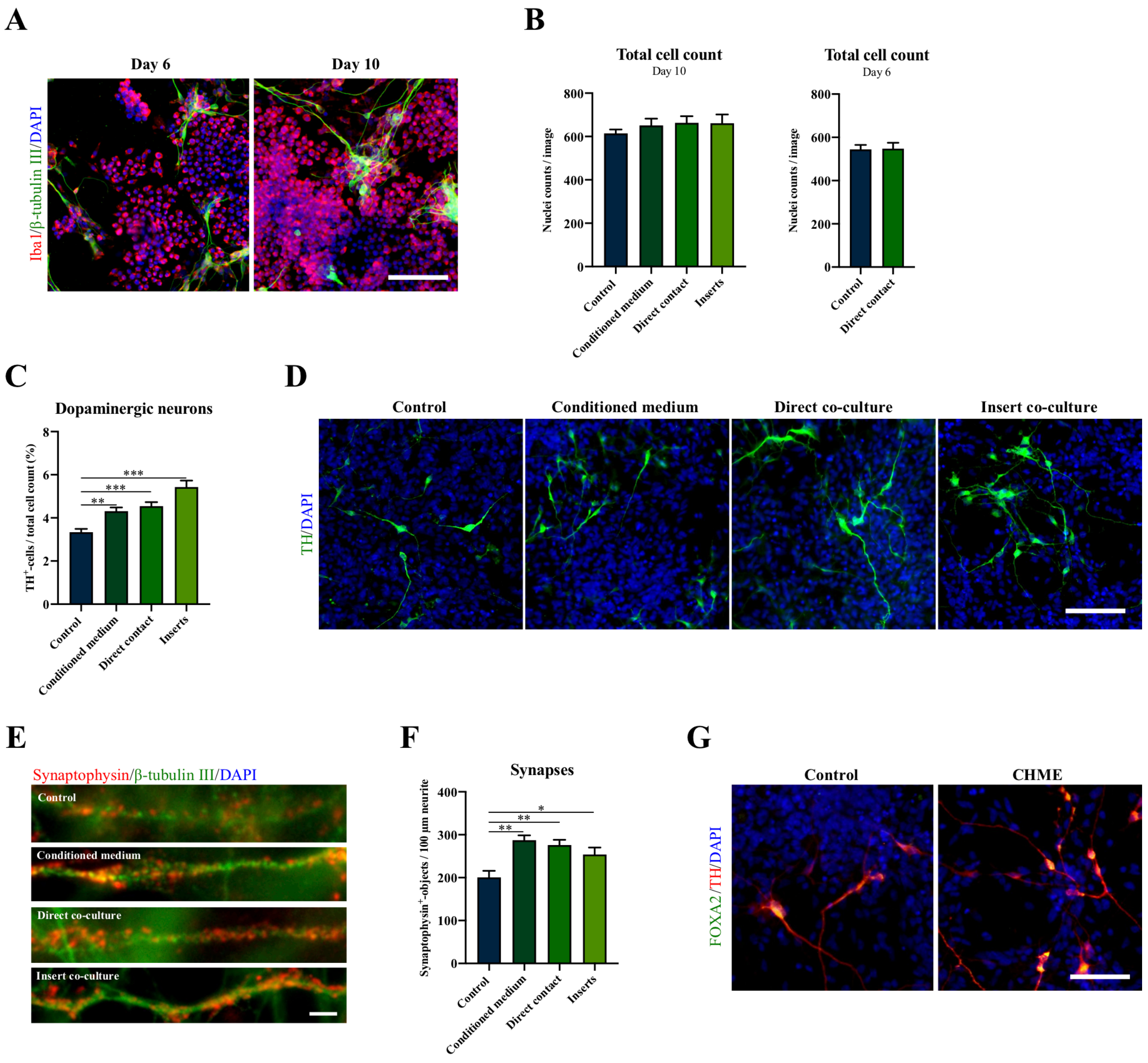

Figure S1. Additional data for the different co-culture setups, Related to Figure 1. (A) Immunofluorescence staining for the microglial marker Iba1 and the pan-neuronal marker β-tubulin III of day 6 and 10 direct co-cultures showing BV2 microglial overgrowth during the direct co-culture differentiation of hVM1-Bcl-X_L NSCs. Scalebar: 100 µm. (B) Total cell counts for the different co-culture setups with BV2 microglia and hVM1-Bcl-X_L NSCs. Day 10: control, n = 17, N= 6; conditioned medium, n = 10, N = 4; direct contact, n = 10, N = 2; inserts, n = 13, N = 4; day 6: control, n = 14, N = 4; direct contact, n = 14, N = 4. (C-D) The different co-culture setups; conditioned media, direct or indirect co-culture using membrane inserts, tested on the iPSC-NSC line XCL1 and the human CHME microglia cell line showing (C) TH^+ neurons/total cell count and (D) representative images of TH^+ neurons. Control, n = 9, N = 2; conditioned medium, direct contact and inserts, n = 6, N = 2. (E-F) Synaptogenesis, quantified as synaptophysin+ objects/100 µm neurite, in the different co-culture setups of iPSC-NSC/CHME. Scalebar: 5 µm. All groups, n = 4, N = 2. (G) Expression of the floorplate marker FOXA2 in TH^+ neurons in indirect iPSC-NSC/CHME co-cultures. Scale bar: 100 µm. One-way ANOVA, Dunnett's multiple comparison test with reference to control. Mean ± SEM. *($p < 0.05$), **($p < 0.01$), ***($p < 0.001$).

Figure S2

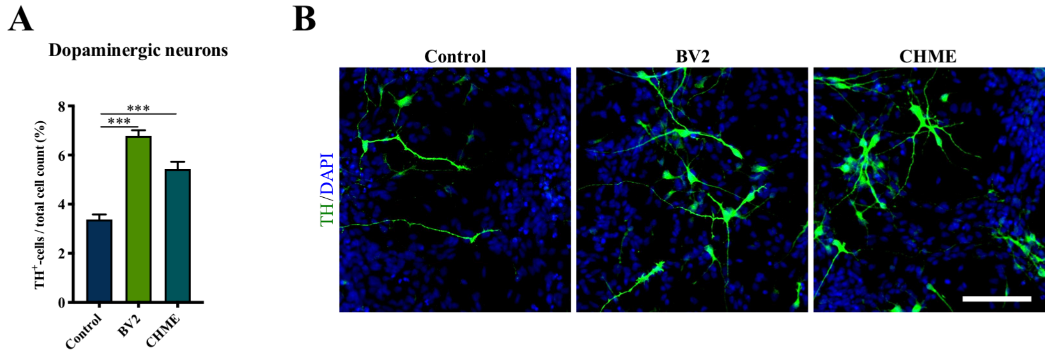

Figure S2. Consistent positive effect of BV2 and CHME microglia on dopaminergic differentiation of the iPSC-NSC line XCL-1, Related to Figure 3. (A) TH^+ neurons/total cell count and (B) representative images of TH^+ neurons. Scalebar: 100 µm. One-way ANOVA, Dunnett's multiple comparison test with reference to control. All groups, n = 6, N = 2. Mean ± SEM. ***(p < 0.001).

Figure S3

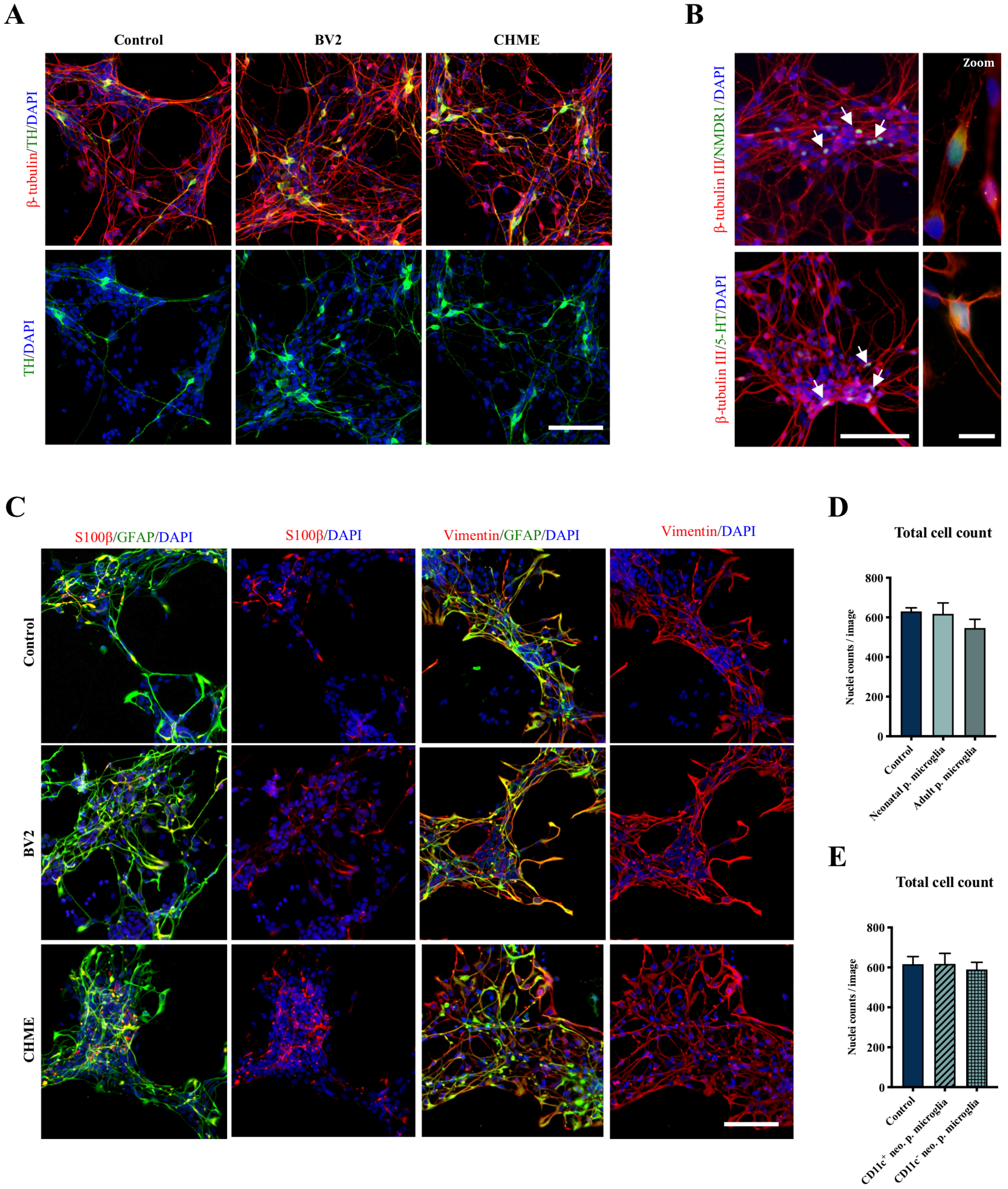

Figure S3. Further culture characterization of differentiated hVM1-Bcl-X$_L$/BV2/CHME co-cultures and effects on total cell count for co-culture with different primary microglial cell types, Related to Figure 3. (A) Immunofluorescence staining for TH and β-tubulin III of hVM1-Bcl-X$_L$ NSCs differentiated in co-culture with BV2 or CHME microglia. Scalebar: 100 µm. (B) The differentiated hVM1-Bcl-X$_L$ co-cultures contained very few glutaminergic (NMDR1$^+$) and serotonergic (5-HT$^+$) cell clusters (>1%). Scalebar: 100 µm. Scalebar zoomed image: 10 µm. (C) GFAP$^+$ astrocytes in the differentiated hVM1-Bcl-X$_L$ co-cultures co-expressed S100β and Vimentin. Scalebar: 100 µm. (D-E) Total cell count of hVM1-Bcl-X$_L$ cells differentiated in co-culture with (D) unsorted neonatal and adult primary microglia; control, n = 11, N = 4; neonatal p. microglia, n = 9, N = 2; adult p. microglia, n = 12, N = 4, or (E) neonatal primary

microglia sorted for CD11c; all groups, n = 8, N = 4. One-way ANOVA, Dunnett's multiple comparison test with reference to control. Mean ± SEM.

Figure S4:

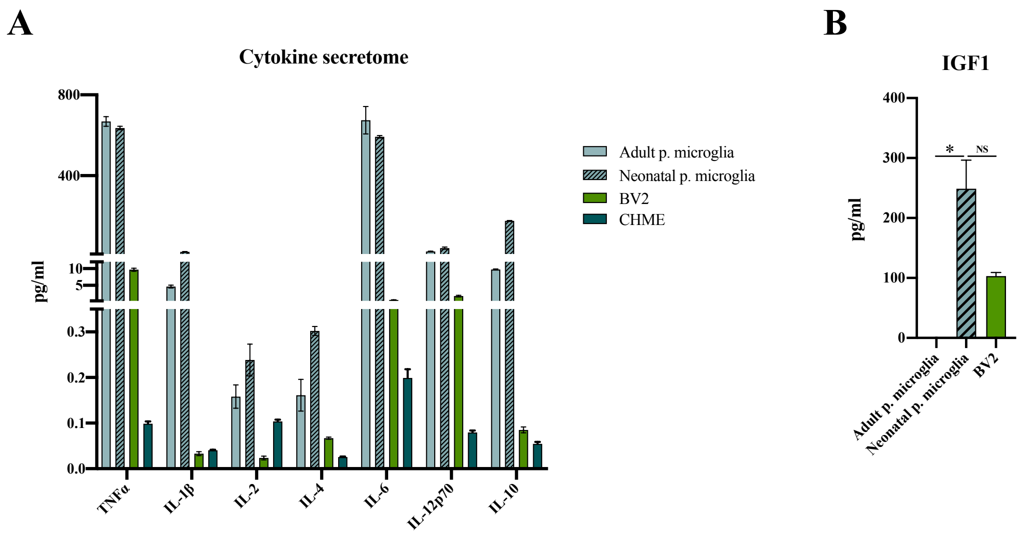

Figure S4. Secretome comparison between BV2, CHME, adult and neonatal primary microglia, Related to Figure 3. (A) Cytokine profiling of medium from adult and neonatal primary microglia, BV2 and CHME microglia. Cytokines were detected for all types of microglia, but higher concentrations were seen for primary microglia (statistical differences not indicated in the figure). All groups, n = 4, N = 2. (B) ELISA for IGF1 of medium from adult and neonatal primary microglia and BV2. All groups, n = 2, N = 2. Values are adjusted for microglia cell density. One-way ANOVA, Tukey's multiple comparison test. Mean ± SEM. *($p < 0.05$), NS = not significant.

Figure S5

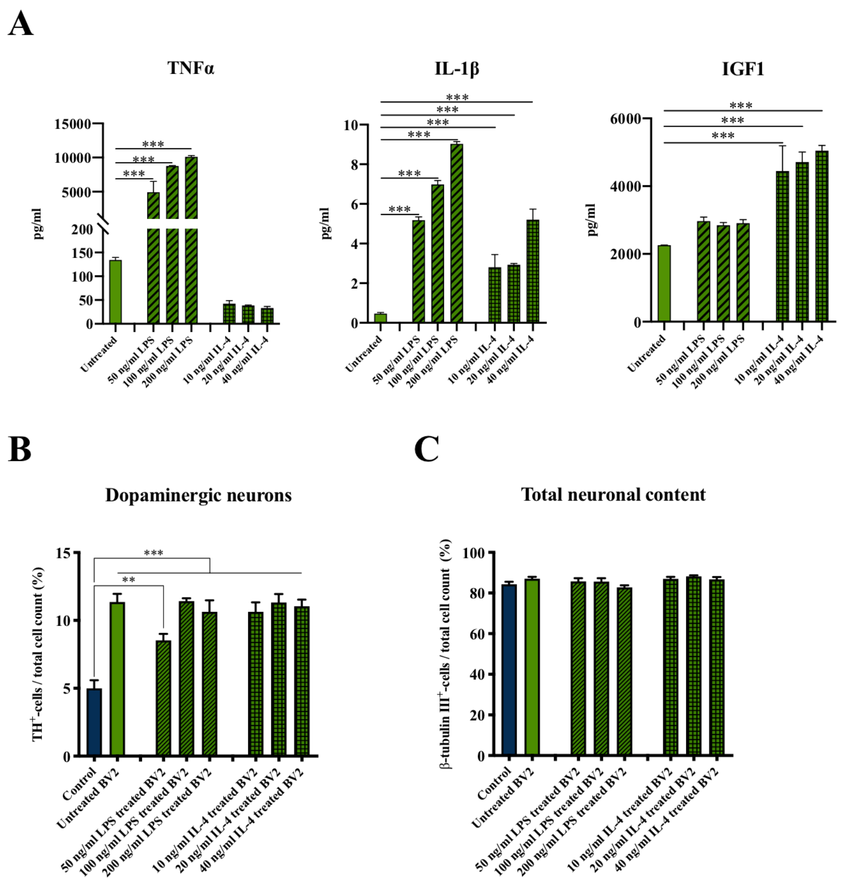

Figure S5. Dose-response data for BV2 microglial activation with LPS and IL-4, Related to Figure 6 and 7. (A) Secretome analysis of selected factors (TNFα, IL-1β and IGF1) in BV2 microglia medium 24 hours post activation with LPS or IL-4. LPS concentrations tested; 50, 100 and 200 ng/ml, IL-4 concentrations tested; 10, 20 and 40 ng/ml. All groups, n = 2-3, N = 2. (B-C) Differentiation outcome of hVM1-Bcl-X_L NSCs in co-culture with activated BV2 microglia evaluated by (B) TH^+ neurons/total cell count and (C) β-tubulin III^+ neurons/total cell count. One-way ANOVA, Dunnett's multiple comparison test with reference to control. All groups, n = 6, N = 2. Mean ± SEM. **($p < 0.01$), ***($p < 0.001$).

Figure S6

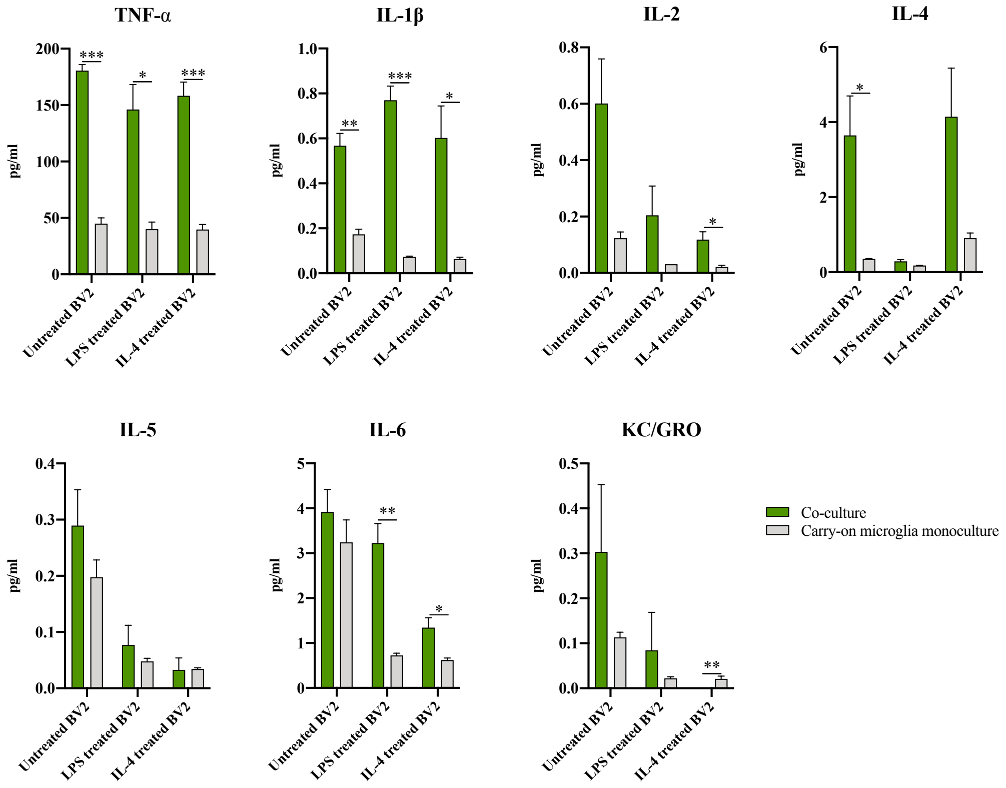

Figure S6. Secretome comparison of BV2 microglia in co-culture vs. carry-on monocultures, Related to Figure 7. Cytokine profiling of medium from day 3 co-cultures compared to day 3 carry-on BV2 monocultures showing a general increased cytokine secretion from co-cultures. Multiple t-test, Holm-Sidak's multiple comparison test. All co-culture groups, n = 4, N = 2; all carry-on groups, n = 3, N = 2. Values are adjusted for microglia cell density. Mean ± SEM. *($p < 0.05$), **($p < 0.01$), ***($p < 0.001$).

Figure S7

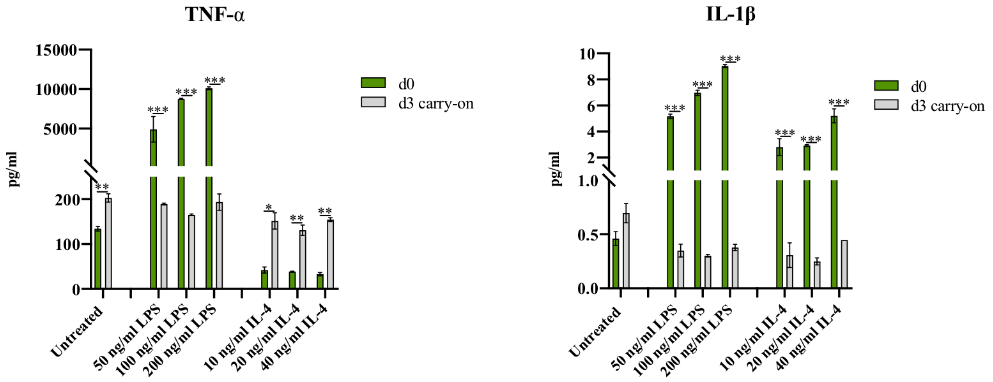

Figure S7. Secretome analysis of activated BV2 carry-on cultures, Related to Figure 6 and 7. Cytokine profiling of medium from day 3 carry-on microglia cultures activated with increasing concentrations of LPS and IL-4 for 24 hours at day 0 (selected cytokines presented; TNFα and IL-1β). Multiple t-test, Holm-Sidak's multiple comparison test. All groups, n = 2-3, N = 2. Mean ± SEM. **($p < 0.01$), ***($p < 0.001$).

Table S1: Result overview for the different co-culture combinations of NSC and microglial cell lines.

**Cell lines: NSCs + microglia**	**hVM1-Bcl-X_L + BV2**	**hVM1-Bcl-X_L + CHME**	**hVM1-Bcl-X_L + neonatal primary microglia**	**hVM1-Bcl-X_L + adult primary microglia**	**hNS1 + BV2**	**iPSC-NSC + BV2**	**iPSC-NSC + CHME**
TH^+ neurons/total cell count	↑*	↑*	↑*	↔	↑*	↑*	↑*
Total cell count	↔	↔	↔	↔	↑*	↑*	-
TH^+ neurons/β-tubulin III^+ neurons	↑*	↑*	↑*	↔	-	-	-
β-tubulin III^+ neurons/total cell count	↔	↔	↔/↑	↔	↑*	-	-
$GABA^+$ neurons/ total cell count	↓	↓*	-	-	-	-	-
$GFAP^+$ astrocytes/ total cell count	↑	↑*	-	-	-	-	-

Abbreviations: NSCs, neural stem cells; TH, tyrosine hydroxylase; GFAP, glial fibrillary acidic protein. Arrows mark whether relative cell counts are increased (↑), decreased (↓) or unchanged (↔). Significant changes are marked with *.

Supplemental Experimental Procedures

Ethics

Human cell lines were used in accordance with Danish national regulations, the ethical guidelines issued by the Network of European CNS Transplantation and Restoration (NECTAR), and the International Society for Stem Cell Research (ISSCR). The Research Ethics Committee of the Region of Southern Denmark approved the study prior to initiation (S-20130101). Ethic statements about the human fetal origin of the NSC lines hVM1-Bcl-X_L and hNS1 can be found in the original paper describing the hVM1-Bcl-X_L cell line (Villa et al., 2009).
All animal experiments were approved by the Danish Animal Experiments Inspectorate (approval number 2014-15-0201-00369).

Propagation and differentiation of NSCs

The NSC lines hVM1-Bcl-X_L and hNS1 were propagated in poly-L-lysine (PLL, 10 µg/ml, Sigma)-coated flasks in HNSC.100 medium consisting of DMEM/F12 with Glutamax (Gibco), 0.6% D-glucose (Sigma), 0.5% 1 M Hepes (Gibco), 0.5% AlbuMAX-I (Gibco), 1% N2 supplement (Gibco), 1% NEAA (Sigma), and 1% Penicillin/streptomycin (Gibco), supplemented with 20 ng/ml recombinant human epidermal growth factor (rh-EGF, R&D Systems) and 20 ng/ml recombinant human basic fibroblast growth factor (rh-bFGF, R&D Systems). Medium change was performed every third day and cells were passaged when 80-90% confluent, by dissociating the cells for 5 min with trypsin-EDTA (Gibco). For differentiation, hVM1-Bcl-X_L cells were seeded onto PLL-coated plates at a density of 50,000 cells/cm^2 and spontaneously differentiated to neurons for 10 days by withdrawal of growth factors (rh-EGF and rh-bFGF) from the HNSC.100 medium. A 50% medium change was performed every third day. The hNS1 cells were seeded onto PLL-coated plates at a density of 50,000 cells/cm^2 and differentiated for 14 days using the CK4 protocol (Krabbe et al., 2009); HNSC.100 medium supplemented with 50 ng/ml recombinant human fibroblast growth factor 8 (rh-FGF8, R&D Systems) for the first three days, followed by HNSC.100 medium supplemented with 25 µM forskolin (Sigma), 5 ng/ml recombinant human glial cell line-derived neurotrophic factor (rh-GDNF, R&D Systems), and 25 ng/ml recombinant human sonic hedgehog (R&D Systems) for the remaining 11 days with a 50% medium change every third day.
The iPSC-derived NSC line XCL1 was propagated on Geltrex (Gibco)-coated plates in Neurobasal medium supplemented with 1x B27 (Gibco), 2 mM NEAA (Gibco), 2 mM GlutaMAX-1 (Gibco), 1% penicillin/streptomycin (Gibco) and 10 ng/ml rh-bFGF (R&D Systems,). Medium change was performed every other day and cells were passaged 1:3 when 80-90% confluent, by dissociating the cells for 5 min with accutase (Gibco). For differentiation iPSC-derived NSCs were seeded onto plates coated with 20 µg/ml poly-L-ornithine (PLO, Sigma) and 10 µg/ml laminin (Life Technologies) at a density of 50,000 cells/cm^2. The first 10 days of differentiation were carried out in DOPA Induction Medium (XCell Science) supplemented with DOPA Induction Supplement A, B and C (XCell Science) and 200 ng/ml rh-SHH (Peprotech) with 50% medium change every other day. At days 5 and 10, cells were passaged using accutase for 5 min and plated at a density of 50,000 cells/cm^2 unless otherwise specified. At day 10, the medium was changed to DOPA Maturation Medium (XCell Science) with DOPA Maturation Supplement A (XCell Science) until day 16 and DOPA Maturation Supplement B (XCell Science) until day 25 with 50% medium change every other day.
NSC cultures were propagated in an incubator at 37°C with 5% CO2 and 95% humidified air containing 20% O_2 and differentiated at either the same conditions or at low O_2 tension (5% CO_2, 92% N_2, and 3% O_2) monitored by an O_2-sensitive alarm system (Forma Scientific Inc., OH, USA).

Propagation of microglial cells

BV2 and CHME microglial cell lines were propagated in PLO (15 µg/ml, Sigma)-coated flasks in RPMI-1640 with Glutamax (Gibco) supplemented with 5% heat inactivated Fetal Bovine Serum (FBS, Gibco) and 1% penicillin/streptomycin (Gibco). The culture medium was changed every second to third day and passaged 1:20 when 80-90% confluent by scraping off cells using a sterile cell scraper.
Neonatal and adult primary microglia were cultured similar to the BV2 and CHME cells (cell isolation is described below).

Mice

C57BL/6j female mice aged 7–8 weeks were obtained from Taconic Europe A/S and maintained as a breeding colony in the Biomedical Laboratory, University of Southern Denmark (Odense). Neonatal C57BL/6j mice (P3-5) and adult mice (8 weeks) used for experiments were of mixed sex.

Primary microglia isolation

The mice were anesthetized with 200 mg/kg pentobarbital and intracardially perfused with ice-cold PBS and brain and spinal cord tissues were collected. For isolating total unfractioned microglia from neonatal and adult mice, tissues were

dissociated using Neural Tissue Dissociation Kit (P) (Miltenyi Biotec) and single cells were collected after centrifugation in 37% Percoll (GE Healthcare Biosciences AB). Microglia were isolated by magnetic separation using CD11b (Microglia) MicroBeads (Miltenyi Biotec). All steps were done according to manufacturer's protocols.
For isolating CD11c$^+$ and CD11c$^-$ neonatal microglia, a single-cell suspension was generated by forcing the tissue through a 70 mm cell strainer (BD Biosciences) and single cells were collected after centrifugation in 37% Percoll. They were first incubated with anti-CD45 (Clone 30-F11; Biolegend), anti-CD11b (Clone M1/70; Biolegend), and biotin conjugated anti-CD11c (Clone HL3; BD Pharmingen) antibodies in PBS with 2% FBS and finally with streptavidin-APC (Biolegend). Cell populations were gated based on isotype-matched control antibodies as CD45dim CD11b$^+$ CD11c$^-$ (CD11c$^-$ microglia), CD45dim CD11b$^+$ CD11c$^+$ (CD11c$^+$ microglia) and sorted on a FACSAriaTM III cell sorter (BD Biosciences).

Immunocytochemistry
Cells were fixed in 4% paraformaldehyde (PFA, Sigma) in 0.15 M phosphate buffer (pH 7.4) for 20 min and washed for 3x15 min in 0.05 M Tris-buffered saline (TBS, pH 7.4) with 0.1% Triton X-100 (Sigma). For DAB staining cells were pre-incubated in TBS containing 10% FBS (Sigma) for 30 min to block unspecific antibody binding before incubation overnight (ON) at 4°C with primary antibodies diluted in TBS/10% FBS serum. Cells were washed 3x15 min in TBS/0.1% Triton X-100 and incubated for 1 hour at room temperature (RT) with biotinylated secondary donkey anti-rabbit IgG (GE Healthcare), sheep anti-mouse IgG (GE Healthcare), or goat anti-rat IgG (Vector Laboratories) diluted 1:200 in TBS/10% FBS serum. After rinsing in TBS/1% Triton-X-100, cells were incubated for 1 hour at RT in horseradish peroxidase (HRP) conjugated streptavidin (GE Healthcare) diluted 1:200 in TBS/10% FBS serum. Cells were then washed in TBS before visualization of the immunocytochemical complexes with 0.01% 3.3'-diaminobenzidine (DAB, Sigma) and 0.015% H_2O_2 (Merck) in TBS followed by mounting using glass coverslips and Aquatex (Merck #108562).
For immunofluorescence staining cells were incubated ON with primary antibodies and washed in TBS/0,1% Triton X-100 as described above. Cells were then incubated with secondary Alexa Fluor 555 goat anti-mouse IgG (Molecular Probes) and/or Alexa Fluor 488 goat anti-rabbit IgG (Invitrogen) diluted 1:500 in TBS/10% FBS serum for 2 hours at RT. Cell nuclei were counterstained with 10 μM 4'.6-diamidino-2-phenylindole (DAPI) (Sigma) for 15 min at RT. Cultures were mounted onto glass slides using ProLong® Diamond (Molecular Probes).
Primary antibodies used: mouse anti-human nuclei (HN, Merck #MAB1281) 1:500, rabbit anti-tyrosine hydroxylase (TH, Merck #AB152) 1:600, mouse anti-TH (Merck #MAB5280) 1:2000, mouse anti-β-tubulin III (Sigma #T8660) 1:2000, rabbit anti-β-tubulin III (Sigma #T2200) 1:2000, mouse anti-synaptophysin (Sigma #S5768) 1: 200, goat anti-FOXA2 (R&D, #AF2400) 1:250, rabbit anti-Iba1 (Wako #019-19741) 1:300, rabbit anti-GABA (Sigma #A2052) 1:2000, rabbit anti-NMDAR-1 (Millipore, #AB9864) 1: 100, mouse anti-5-HT (DAKO #M0758) 1:1000, rabbit anti-glial fibrillary acidic protein (GFAP, DAKO #Z0334) 1:4000, mouse anti-S100β (Sigma S2532) 1: 1000, mouse anti-vimentin (Santa Cruz #SC373717) 1:200, mouse anti-microtubule-associated protein 2a+b (MAP2, Sigma #M1406) 1:2000, and mouse anti-ki67 (BD Pharmingen #550609) 1:500, rabbit anti-cleaved caspase 3 (Cell Signaling #9661) 1: 400.

Bioimaging and image analysis
Cell counts on 3.3'-diaminobenzidine (DAB) stained cultures were performed by bright-field microscopy (Olympus) in 16 randomly selected areas per well using an ocular grid (Olympus) and normalized to total cell numbers as quantified from HN counts.
Fluorescence images were acquired either on a fluorescence microscope (Olympus) for five randomly chosen areas per coverslip or using an ImageXpress automated imaging system (Molecular Device). Cell counts were performed using the Cell Counter plugin for ImageJ and normalized to the total cell numbers as quantified by CellProfiler analysis for DAPI$^+$ nuclei (Carpenter et al., 2006; Schneider et al., 2012). Synaptophysin$^+$ objects were quantified automatically in ImageJ and normalized to neurite length. Morphological analysis was performed on hNS1-derived TH$^+$ neurons using the NeuronJ plugin for ImageJ (Meijering et al., 2004).

Western blotting
Cell pellets were lysed in phosphate buffered saline (PBS) with 1% Triton-X-100 and protease inhibitor (Complete Tablets, Roche) and sonicated for 3x10 sec at amplitude 2 microns on ice. Protein concentrations were measured with bicinchoninic acid assay (BCA, Pierce) and equal amounts of protein from each sample were denatured for 10 min at 70ºC in PBS. Proteins (10 μg) were separated on 4-12% Bis-Tris gels (NuPAGE) at 200V for 50 min with MOPS running buffer (NuPAGE) supplemented with 0.25% antioxidant (NuPAGE) and transferred to a polyvinylidene difluoride (PVDF) membranes (Invitrogen) at 20V for 8 min using the iBlot transfer system (Invitrogen). Plus2 Prestained Protein Standard (SeeBlue) was used to estimate the molecular weight of the proteins. Membranes were blocked for 60 min at 4°C in 5% skim milk (Natur Drogeriet) diluted in TBS/0.05% Tween-20 before incubation ON at 4°C with primary antibodies diluted in TBS/0.05% Tween-20. After repeated washing in TBS/0.05% Tween-20, membranes were incubated for 1 hour at RT with HRP-conjugated secondary rabbit anti-mouse IgG (DAKO) diluted 1:2000 in TBS/0.05% Tween-

20. Subsequently, membranes were repeatedly washed in TBS/0.05% Tween-20 and developed using luminol-based enhanced chemiluminescence (ECL kit, ThermoFisher Scientific) on a ChemiDoc MP imaging system (Bio-Rad). As loading control, all blots were subsequently incubated ON at 4°C with mouse anti-α actin antibody (Merck) diluted 1:6000 in TBS/0.05% Tween-20 and developed as described above.

RNA isolation, cDNA synthesis, and quantitative real-time polymerase chain reaction
For mRNA extraction, co-culture and control differentiated hVM1-Bcl-X_L cells were harvested in Trizol lysis reagent (Life Technologies) at day 10 of differentiation and lysed by vortexing for 30 sec. The RNA was purified using the RNeasy Mini kit (Qiagen) and treated with DNaseI kit according to the manufacturer's instruction. cDNA was synthesized from 250 ng of total RNA using the High Capacity cDNA Archive Kit (Applied Biosystems, Thermo Fischer). Real-time PCR of selected genes was performed using TaqMan-probe assays in an AB fast-7900HT System (Applied Biosystems, Thermo Fisher) under standard running conditions. Predesigned TaqMan assays are listed in Supplemental Information. Three replicates were run for each combination of genes and samples. Ct assignment was performed using the Sequence Detection System 2.4 software (Applied Biosystems) to set baseline and threshold parameters. Results were analyzed using the comparative method (2^{-ddCt}) and normalized to endogenous expression of *18S*, *GAPDH*, and *HPRT*.

TaqMan qPCR primers:

**Target**	**Assay ID**
VMAT2	Hs00161858_m1
DAT	Hs00168988_m1
AADC	Hs01105042_m1
PITX3	Hs00374504_m1
EN1	Hs00154977_m1
LMX1A	Hs00602600_m1
GAPDH	Hs02758991_g1
HPRT	Hs02800695_m1
18S	Hs03003631_g1

Cell death measurements
Nuclear morphological analysis was performed at day 5, 10, and 25 during differentiation of iPSC-derived NSCs. The number of fragmented DAPI$^+$-nuclei was used to estimate apoptotic cell death, and the number of pyknotic DAPI$^+$-nuclei was used to estimate necrotic cell death in the cultures (Dindler et al., 2018). Nuclear counting was performed in a blinded manner and normalized to total nuclei count.
Necrotic cell death was additionally estimated from lactate dehydrogenase release in iPSC-derived neural cultures during differentiation (day 5, 10 and 25) using the CytoTox96® Non-Radioactive Cytotoxicity Assay (Promega). The assay was performed according to the manufacturer's protocol.

LPS and IL-4 dose-response activation
BV2 microglia were activated with increasing concentrations of LPS (from Escherichia coli O111:B4, Sigma) and IL-4 (Peprotech) 24 hours prior to co-culture setup with hVM1-Bcl-X_L NSCs. Concentrations applied: LPS; 50, 100 and 200 ng/ml, IL-4; 10, 20 and 40 ng/ml.

TNFα, IL-1β and IGF1 treatment
The hVM1-Bcl-X_L NSC line were spontaneously differentiated for 10 days with addition of TNFα, IL-1β or IGF1 at day 0 in a dose-response setup. 50% media change were performed every third day. Five concentrations were tested for each factor; three concentrations matching the detected levels in the conditioned co-culture medium and two concentrations matching what has previously been used in the literature (Doherty, 2007; Ling et al., 1998; Supeno et al., 2013): TNFα; 100 pg/ml, 200 pg/ml, 400 pg/ml, 10 ng/ml and 20 ng/ml, IL-1β; 0.5 pg/ml, 1 pg/ml, 2 pg/ml, 100 pg/ml and 200 pg/ml, IGF1; 1 ng/ml, 6 ng/ml, 12 ng/ml, 100 ng/ml and 200 ng/ml.
The iPSC-NSC line XCL1 was differentiated for 25 days using the DOPA differentiation kit (XCell Science) with exposure to either 10 ng/ml TNFα, 100 pg/ml IL-1β or 100 ng/ml IGF1 during the first five days of differentiation.

Graphical illustrations:
Illustrations were made using BioRender and PowerPoint.

Supplemental References

Carpenter, A.E., Jones, T.R., Lamprecht, M.R., Clarke, C., Kang, I.H., Friman, O., Guertin, D.A., Chang, J.H., Lindquist, R.A., Moffat, J., *et al.* (2006). CellProfiler: image analysis software for identifying and quantifying cell phenotypes. Genome Biol *7*, R100.

Dindler, A., Blaabjerg, M., Kamand, M., Bogetofte, H., and Meyer, M. (2018). Activation of Group II Metabotropic Glutamate Receptors Increases Proliferation but does not Influence Neuronal Differentiation of a Human Neural Stem Cell Line. Basic Clin Pharmacol Toxicol *122*, 367-372.

Doherty, G.H. (2007). Developmental switch in the effects of TNFalpha on ventral midbrain dopaminergic neurons. Neurosci Res *57*, 296-305.

Krabbe, C., Courtois, E., Jensen, P., Jorgensen, J.R., Zimmer, J., Martinez-Serrano, A., and Meyer, M. (2009). Enhanced dopaminergic differentiation of human neural stem cells by synergistic effect of Bcl-xL and reduced oxygen tension. J Neurochem *110*, 1908-1920.

Ling, Z.D., Potter, E.D., Lipton, J.W., and Carvey, P.M. (1998). Differentiation of mesencephalic progenitor cells into dopaminergic neurons by cytokines. Exp Neurol *149*, 411-423.

Meijering, E., Jacob, M., Sarria, J.C., Steiner, P., Hirling, H., and Unser, M. (2004). Design and validation of a tool for neurite tracing and analysis in fluorescence microscopy images. Cytometry A *58*, 167-176.

Schneider, C.A., Rasband, W.S., and Eliceiri, K.W. (2012). NIH Image to ImageJ: 25 years of image analysis. Nat Methods *9*, 671-675.

Supeno, N.E., Pati, S., Hadi, R.A., Ghani, A.R., Mustafa, Z., Abdullah, J.M., Idris, F.M., Han, X., and Jaafar, H. (2013). IGF-1 acts as controlling switch for long-term proliferation and maintenance of EGF/FGF-responsive striatal neural stem cells. Int J Med Sci *10*, 522-531.

Villa, A., Liste, I., Courtois, E.T., Seiz, E.G., Ramos, M., Meyer, M., Juliusson, B., Kusk, P., and Martinez-Serrano, A. (2009). Generation and properties of a new human ventral mesencephalic neural stem cell line. Exp Cell Res *315*, 1860-1874.
