# Supplementary material for: Microglia-Secreted Factors Enhance Dopaminergic Differentiation of Tissue- and iPSC-Derived Human Neural Stem Cells
Source: Stem Cell Reports. 2021 Jan 21;16(2):281–94. doi: 10.1016/j.stemcr.2020.12.011 (PMC7878834; doi:10.1016/j.stemcr.2020.12.011)
Supplement: Document S2. Article plus Supplemental Information [file mmc2.pdf]

Stem Cell Reports
Article
ISSCR
OPEN ACCESS

Microglia-Secreted Factors Enhance Dopaminergic Differentiation of Tissue- and iPSC-Derived Human Neural Stem Cells

Sissel Ida Schmidt,[1] Helle Bogetofte,[1] Louise Ritter,[1] Jette Bach Agergaard,[1] Ditte Hammerich,[1] Amina Arslanagic Kabiljagic,[1] Agnieszka Wlodarczyk,[1] Silvia Garcia Lopez,[2] Mia Dahl Sørensen,[3] Mie Lærkegård Jørgensen,[1] Justyna Okarmus,[1] Alberto Martínez Serrano,[2] Bjarne Winther Kristensen,[3,6] Kristine Freude,[5] Trevor Owens,[1,6] and Morten Meyer[1,4,6,*]
[1]Department of Neurobiology Research, Institute of Molecular Medicine, University of Southern Denmark, Odense, DK
[2]Department of Molecular Biology and Center of Molecular Biology Severo Ochoa, University Autonoma Madrid-C.S.I.C., Madrid, ES
[3]Department of Pathology, Odense University Hospital, Odense, DK
[4]Department of Neurology, Odense University Hospital, Odense, DK
[5]Faculty of Health and Medical Sciences, Department of Veterinary and Animal Sciences, Section for Pathobiological Sciences, University of Copenhagen, Copenhagen, DK
[6]BRIDGE – Brain Research Inter-Disciplinary Guided Excellence, Department of Clinical Research, University of Southern Denmark, Odense, DK
*Correspondence: mmeyer@health.sdu.dk
https://doi.org/10.1016/j.stemcr.2020.12.011

SUMMARY

Microglia have recently been established as key regulators of brain development. However, their role in neuronal subtype specification remains largely unknown. Using three different co-culture setups, we show that microglia-secreted factors enhance dopaminergic differentiation of somatic and induced pluripotent stem cell-derived human neural stem cells (NSCs). The effect was consistent across different NSC and microglial cell lines and was independent of prior microglial activation, although restricted to microglia of embryonic origin. We provide evidence that the effect is mediated through reduced cell proliferation and decreased apoptosis and necrosis orchestrated in a sequential manner during the differentiation process. tumor necrosis factor alpha, interleukin-1β, and insulinlike growth factor 1 are identified as key mediators of the effect and shown to directly increase dopaminergic differentiation of human NSCs. These findings demonstrate a positive effect of microglia on dopaminergic neurogenesis and may provide new insights into inductive and protective factors that can stimulate *in vitro* derivation of dopaminergic neurons.

INTRODUCTION

Neurogenesis is a complex process comprising several steps that require regulation by the microenvironment. Neural stem cell (NSC) proliferation and differentiation, migration of neuroblasts to their appropriate location, survival of immature and mature neurons, and construction of synaptic connectivity thus all rely on extrinsic cues (Ekdahl et al., 2009).

Microglia are immune cells in the central nervous system (CNS) that originate from hematopoietic progenitors of the yolk sac and start to colonize the developing brain as early as the fourth gestational week (Menassa and Gomez-Nicola, 2018). As microglia are present in the brain before the emergence of neurons and other glia, they may play an important role in providing a proper microenvironment for embryonic neurogenesis. This hypothesis is supported by studies showing that colony-stimulating factor 1 receptor knockout mice, which lack microglia, display abnormal brain development (Elmore et al., 2014), and that microglia can induce developmental apoptosis and thereby regulate the size of the neural precursor pool (Cunningham et al., 2013; Marin-Teva et al., 2004; Tronnes et al., 2016; Wakselman et al., 2008). In addition, microglia have been reported to modulate synaptogenesis through local synthesis of neurotrophic factors (Miyamoto et al., 2016; Parkhurst et al., 2013), participate in synaptic pruning (Paolicelli et al., 2011; Schafer et al., 2012), and guide axonal outgrowth (Pont-Lezica et al., 2014; Squarzoni et al., 2014).

Studies suggest that the neurogenic effect of microglia is dependent on their activation state and is mediated by their cytokine release (Shigemoto-Mogami et al., 2014; Wang et al., 2007). Following pro-inflammatory activation (e.g., by stimulation with lipopolysaccharide [LPS]), microglia secrete pro-inflammatory cytokines including tumor necrosis factor alpha (TNFα), interleukin (IL)-1β, interferon-γ (IFN-γ), and nitric oxide and reduce their release of neurotrophic factors. In contrast, stimulation with, e.g., IL-4 causes microglia to secrete anti-inflammatory cytokines including IL-4 and IL-10, and neurotrophic factors such as brain-derived neurotrophic factor (BDNF), glial cell line-derived neurotrophic factor (GDNF), and insulin growth factor 1 (IGF1) (Franco and Fernandez-Suarez, 2015; Polazzi and Monti, 2010). While the pro-inflammatory activated microglial cells generally appear to inhibit differentiation and proliferation of NSCs and cause aberrant migration of newly formed neurons in the adult rat hippocampus (Ekdahl et al., 2003; Monje et al., 2003; Yang et al., 2010), the anti-inflammatory activated microglia have a neuroprotective role and can increase

This is an open access article under the CC BY license (http://creativecommons.org/licenses/by/4.0/).

neurogenesis and oligodendrogenesis of NSCs (Butovsky et al., 2006; Yuan et al., 2017). However, it remains unclear how the effects mediated by pro- and anti-inflammatory secreted molecules is influenced by NSC-microglia interactions (Mosher et al., 2012; Su et al., 2014).

Microglia are widely, but not uniformly, distributed throughout the CNS. In the rodent brain, they are present in higher density in many brain regions containing dopaminergic neurons or dopaminergic projections, such as the substantia nigra, striatum, hippocampus, and the olfactory system, compared with surrounding structures (De Biase et al., 2017; Lawson et al., 1990). This suggests that the microenvironment offered by the microglia might be important for dopaminergic neuronal development in particular, providing potential new insights into inductive factors for dopaminergic differentiation.

To address the role of microglia and their activation state in dopaminergic neurogenesis, we investigated whether microglia-secreted factors enhanced dopaminergic differentiation of somatic and induced pluripotent stem cell (iPSC)-derived human NSCs *in vitro*. We found that, independent of the microglial cell type and NSC lines, co-culturing NSCs with microglia during differentiation increased the content of dopaminergic neurons in the cultures. This effect was limited to microglia of embryonic origin and was not influenced by prior microglial activation. Microglial-secreted TNFα, IL-1β, and IGF1 were identified as key mediators, and recombinant TNFα, IL-1β, and IGF1 were shown to directly increase dopaminergic differentiation.

RESULTS

Microglia-Secreted Factors Enhance Dopaminergic Differentiation of Human NSCs

Three different co-culture settings were applied to investigate the effect of microglia on dopaminergic differentiation of human NSCs. During 10 days of spontaneous differentiation, hVM1-Bcl-X_L NSCs (human ventral mesencephalic NSCs) were either grown in BV2 microglia-conditioned medium or directly (physical contact) or indirectly (separated by semi-porous membrane inserts) co-cultured with BV2 microglia (Figure 1A). Upon differentiation, the number of cells positive for the pan-neuronal marker β-tubulin III had increased for the direct co-culture group (Figures 1B and 1C), whereas the number of dopaminergic tyrosine hydroxylase-positive (TH^+) neurons was significantly increased in all groups compared with control (Figure 1D and 1B). As BV2 microglia were overgrowing the direct co-culture (Figure S1A), an earlier time point (day 6) was also examined, showing a similar effect on TH^+ neuronal content. When calculating the percentage of TH^+ neurons relative to the total neuronal population (TH/β-tubulin III ratio), only the direct and indirect co-culture setups revealed a significant increase in the yield of TH^+ neurons (Figure 1E). No difference in the total cell numbers was observed (Figure S1B).

The high density of microglia in the direct co-culture group caused the neurons to cluster together at day 10 (Figures 1B and S1A). We therefore investigated whether this growth pattern influenced the neuronal connectivity in the cultures and found an increased synaptic density (synaptophysin$^+$ objects/100 μm neurite) and a more differentiated morphology for neurons in the indirect co-culture group compared with control (Figures 1H and 1I). Verification of the data using other NSC and microglia cell lines revealed that the positive effects on TH^+ neuronal yield and synaptic density were consistent across all groups compared with control (Figures S1C–S1F).

We have previously shown that exposing NSCs during differentiation to physiological oxygen (O_2) tension (3%–5%) rather than atmospheric O_2 tension (20%), which is the standard for most cell culture incubators, enhances the dopaminergic differentiation outcome (Krabbe et al., 2014). Differentiating hVM1-Bcl-X_L NSCs at physiological O_2 tension in co-culture with BV2 microglia using membrane inserts further increased the yield of TH^+ neurons (Figures 1F and 1G).

Since increased numbers of TH^+ cells were detected at all co-culture conditions, the effect of microglia was most likely mediated by secreted factors and unlikely to be dependent on physical cell-cell interaction. Based on cell morphology and the yield of TH^+ neurons, we therefore concluded that the optimal co-culture differentiation setup would be achieved using semi-porous membrane inserts combined with physiological O_2 tension, which was applied for the remaining experiments.

To further characterize the population of TH^+ neurons, qRT-PCR was performed for additional dopaminergic neuronal markers (Figure 2A). Co-culture with microglia significantly increased the expression of the plasma membrane dopamine transporter (*DAT*), the vesicular monoamine transporter 2 (*VMAT2*), and the midbrain-specific transcription factors pituitary homeobox 3 (*PITX3*) and homeobox protein engrailed-1 (*EN1*) genes. The expression of the aromatic amino acid decarboxylase (*AADC*) and the midbrain dopaminergic LIM homeobox transcription factor 1-alpha (*LMX1A*) was not significantly changed. Midbrain characteristics of the TH^+ neurons were also confirmed by FOXA2 immunostaining (Figure 2B).

Enhanced Dopaminergic Differentiation Is Consistently Found When Combining Different Microglial Cells and NSCs

We next investigated how consistent the positive effect of microglia on dopaminergic differentiation was for different

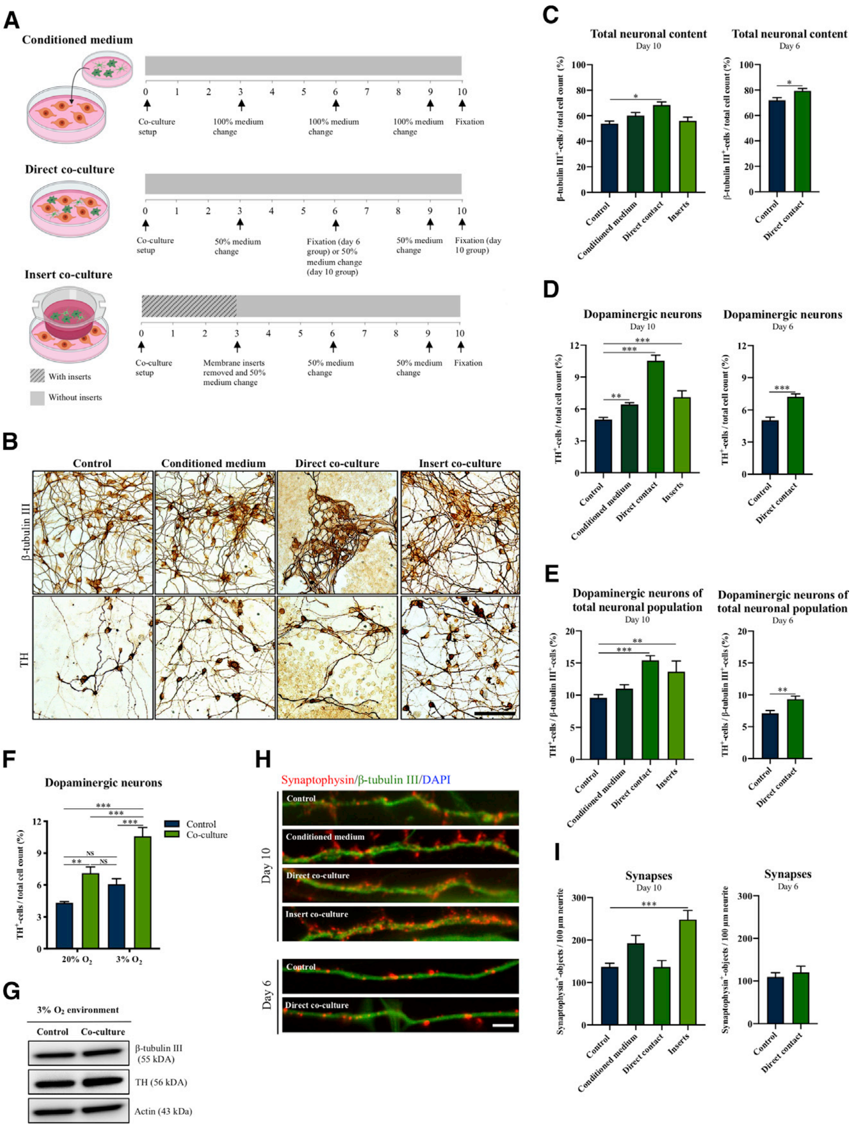

Figure 1. Increased Dopaminergic Differentiation of NSCs Using Different Microglia Co-culture Setups

(A) hVM1-Bcl-X_L NSCs were either exposed to BV2 microglia-conditioned medium, directly co-cultured with BV2 microglia (physical contact), or indirectly co-cultured (separated by semi-porous membrane inserts).

(B–E) Immunocytochemical staining and quantification of differentiated neurons for (C) β-tubulin III^+ neurons/total cell count, (D) TH^+ neurons/total cell count, and (E) the number of TH^+ neurons/β-tubulin III^+ neurons. Scale bar: 100 μm. One-way ANOVA, Dunnett's multiple comparison test with reference to control. Day 10: control, n = 23, N = 6; conditioned medium, n = 16, N = 4; direct contact, n = 6, N = 2; inserts, n = 13, N = 4. Day 6: control, n = 14, N = 4; direct contact, n = 14, N = 4.

(F and G) TH^+ neurons/total cell count and Western blotting for β-tubulin III and TH in differentiated hVM1-Bcl-X_L NSCs cultures after combining the indirect co-culture setup with physiological O_2tension (3% O_2). Two-way ANOVA, Tukey's multiple comparison test. Control, n = 14, N = 4; co-culture, n = 13, N = 4.

(H and I) $Synaptophysin^+$ objects/100 μm neurite. Scalebar: 5 μm. One-way ANOVA, Dunnett's multiple comparison test with reference to control. Day 10: control, n = 6, N = 2; conditioned medium, n = 6, N = 2; direct contact, n = 4, N = 2, inserts, n = 6, N = 2. Day 6: control, n = 4, N = 2; direct contact, n = 4, N = 2. Mean ± SEM. $*p < 0.05$, $**p < 0.01$, $***p < 0.001$, NS = not significant. See Figures S1 for additional data.

microglial and NSC lines. Human hVM1-Bcl-X_L cells were spontaneously differentiated for 10 days in co-culture with either murine BV2 microglia or human CHME microglia cells (Figure 3A). Both microglial cell lines were able to significantly enhance the number of TH^+ neurons as evaluated both relative to total cell count and the TH/β-tubulin III ratio (Figures 3B, 3C, and S3A). This was verified using another NSC line (Figure S2). Surprisingly, the increased numbers of TH^+ neurons were not reflected in either the total cell count or the total neuronal cell count, which revealed no difference for either of the co-culture groups compared with control (Figures 3D and 3E). Taken together, these results suggest that, while the effect on the TH^+ neuronal content was consistently increased across the microglial cell lines, it was not mediated by an overall increased neurogenesis. To investigate if this was due to a shift in the cellular composition of the culture, we characterized the content of other cell types known to be present in the midbrain (Morello and Partanen, 2015). This revealed that the increase in the content of TH^+ neurons might be at the expense of GABAergic differentiation as the number of $GABA^+$ neurons was decreased for both co-culture groups (BV2 and CHME microglia) compared with control; however, it was only significant for the CHME group (Figures 3F, 3G, and 3I). Concerning other neuronal subpopulations, the cultures contained very few glutamatergic (<1%) and serotonergic (<1%) neurons (Figure S3B), and no obvious difference between the groups was seen. In contrast, the numbers of glial fibrillary acidic protein $(GFAP)^+$ astrocytes were increased for both groups, but again were only significant for the CHME microglial cell line (Figures 3H and 3I). The GFAP staining was found

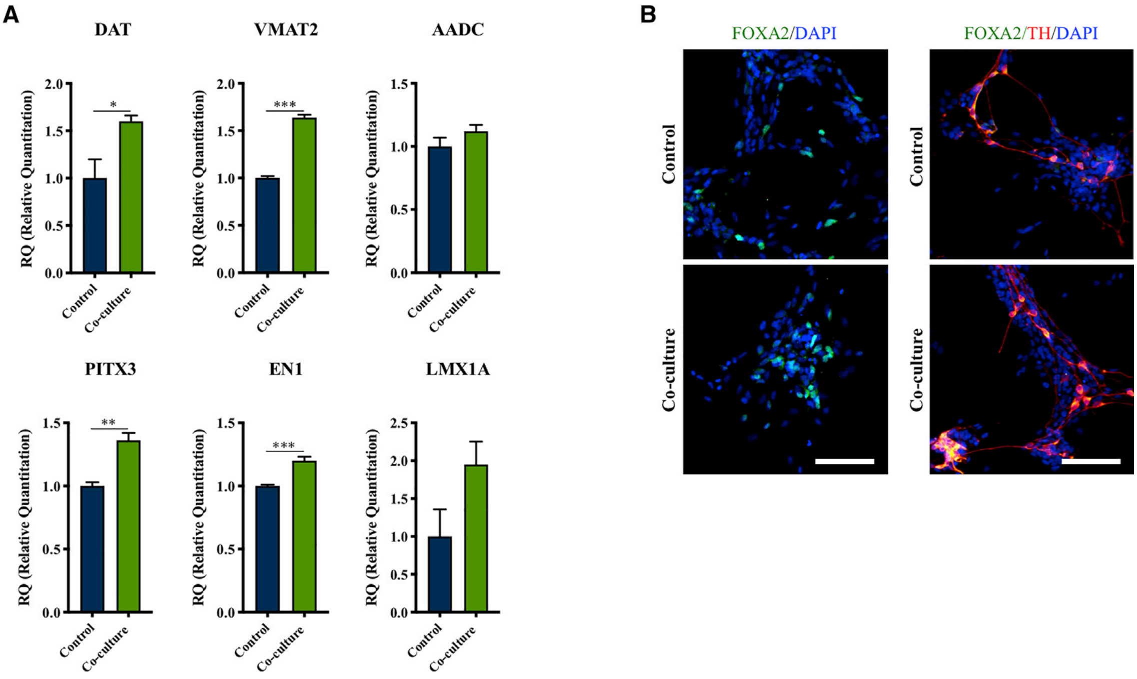

Figure 2. Microglia Co-culture Increased Expression of Dopaminergic and Midbrain-Specific Markers

(A) qRT-PCR data for the plasma membrane *DAT*, *VMAT2*, the midbrain-specific transcription factors *PITX3*, the homeobox protein *EN1*, *AADC*, and the midbrain dopaminergic *LMX1A* upon co-culture differentiation of hVM1-Bcl-X_L NSCs and BV2 microglia. Student's t test. Control, n = 4, N = 2; co-culture, n = 4, N = 2. Mean ± SEM. *$p < 0.05$, **$p < 0.01$, ***$p < 0.001$.

(B) Expression of the floorplate marker FOXA2 in TH^+ neurons. Scale bar: 100 μm.

to co-localize with the markers S100β and vimentin (Figure S3C), confirming the presence of astrocytes.

The positive effect of co-culturing hVM1-Bcl-X_L NSCs with microglia during dopaminergic differentiation was further verified using primary microglia from either P3-P5 pups or adult mice (Figure 3J). Interestingly, only neonatal primary microglia increased the TH^+ neuronal yield, while the total neuronal content and total cell numbers were unchanged for both types of microglia (Figures 3K–3M and S3D). It was recently discovered that a $CD11c^+$ neonatal microglia subtype plays a key role in myelination and neurogenesis in the developing mouse brain through release of IGF1 (Wlodarczyk et al., 2017). We therefore next investigated if a $CD11c^+$ subtype of neonatal primary microglia was responsible for the positive effect of microglia on dopaminergic differentiation. Interestingly, both the $CD11c^+$ and $CD11c^-$ neonatal primary microglia were able to increase the number of TH^+ neurons, whereas the TH/β-tubulin III ratio was unchanged compared with control (Figures 3N–3O). Contrary to the effect of the BV2 and CHME microglia cell lines, $CD11c^+$ neonatal primary microglia increased the total neuronal yield, but no difference was observed for the total cell number (Figures 3P and S3E). A comparative secretome analysis revealed that both BV2, CHME, adult and neonatal primary microglia secreted all of the investigated cytokines, but considerably higher levels were detected for adult and neonatal primary microglia. Only IGF1 was not detected in conditioned culture medium from adult primary microglia (Figure S4).

We then investigated if the dopaminergic neurogenic effect of microglia was consistent across different NSC lines. The human forebrain NSC line hNS1, the iPSC-derived NSC line XCL1, and the hVM1-Bcl-X_L cell line were all differentiated in co-culture with BV2 microglia, resulting in consistently enhanced TH^+ neuronal content (Figures 4A, 4B, and 4E). Interestingly, the total cell number following differentiation was markedly increased for the hNS1 and iPSC-NSC co-culture setups, but not for hVM1-Bcl-X_L (Figures 4C and 4E). Similarly, the number of β-tubulin III^+ cells in the hNS1 cultures was significantly increased for the co-culture group, which contradicted the results obtained for hVM1-Bcl-X_L cells (Figures 4D and 4E). Due to the iPSC-NSC cultures being highly confluent, β-tubulin III cell quantification was not feasible. However, iPSC-NSC cultures immunostained for β-tubulin III displayed a markedly denser staining pattern in the co-culture group (Figure 4E). Table S1 summarizes the cell counts for all co-culture combinations of NSC and microglia cell lines presented here.

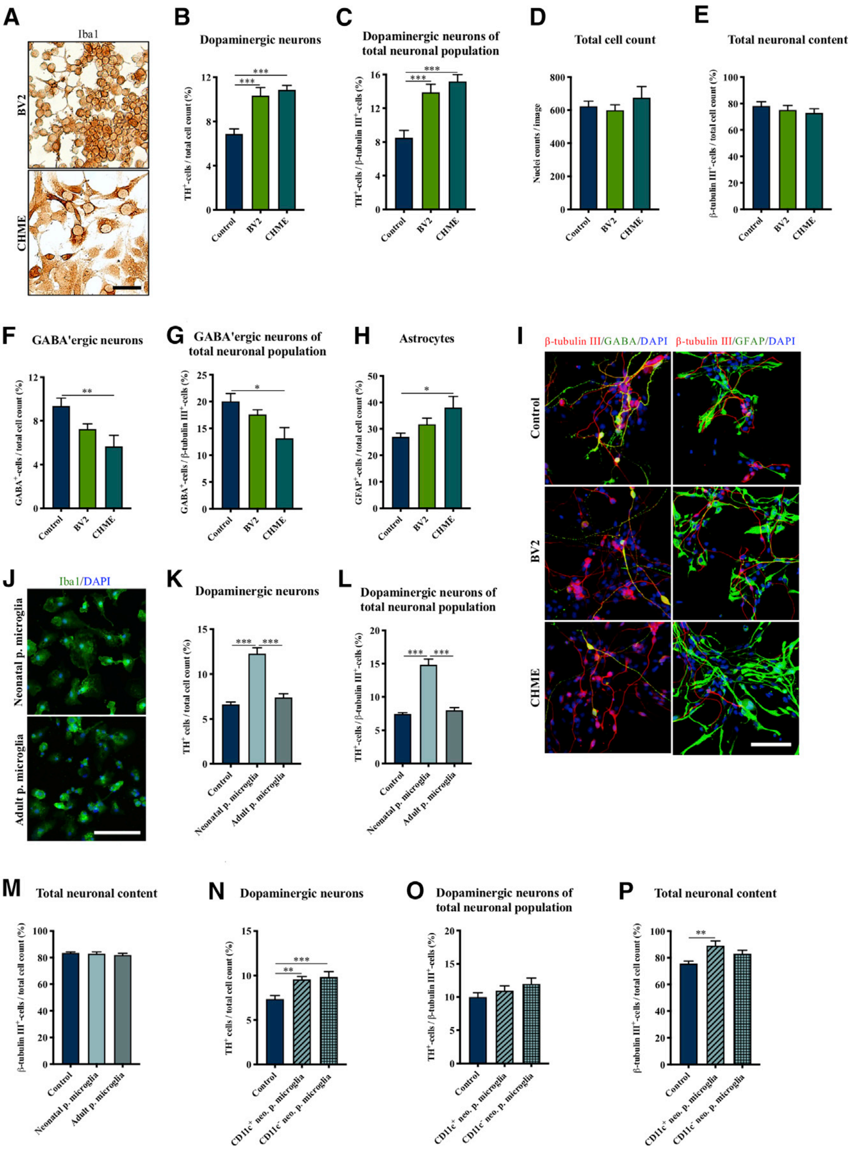

Figure 3. Consistent Positive Effect on Dopaminergic Differentiation Across Different Microglia Cell Lines

(A) Immunocytochemical staining of murine BV2 and human CHME microglia cells for the microglial marker Iba1. Scale bar: 50 μm.

(B–E) Immunocytochemical staining and quantification of hVM1-Bcl-X_L NSC-derived neurons after CHME and BV2 microglia co-culture differentiation for (B) TH^+ neurons/total cell count, (C) TH^+ neurons/β-tubulin III^+ neurons, (D) total cell count, and (E) β-tubulin III^+ neurons/total cell count. Control, n = 21, N = 8; BV2, n = 15, N = 5; CHME, n = 12, N = 5.

(F–I) Immunofluorescence staining and quantification of (F and I) $GABA^+$ neurons/total cell count, (G) $GABA^+$ neurons/β-tubulin III^+ neurons, and (H and I) $GFAP^+$-astrocytes/total cell count. Scale bar: 100 μm. Control, n = 6, N = 2; BV2, n = 6, N = 2; CHME, n = 6, N = 2.

(J) Immunofluorescence staining of neonatal and adult primary microglia (p. microglia) for the microglial marker Iba1. Scale bar: 100 μm.

(K–M) Quantification of (K) TH^+ neurons/total cell count; control, n = 18, n = 4; neonatal p. microglia, n = 9, N = 2; adult p. microglia, n = 12, N = 4, (L) β-tubulin III^+ neurons/total cell count, and (M) TH^+ neurons/β-tubulin III^+ neurons; control, n = 14, n = 4; neonatal p. microglia, n = 9, N = 2; adult p. microglia, n = 8, N = 2, after differentiation of hVM1-Bcl-X_L NSCs in co-culture with murine neonatal or adult p. microglia.

(N–P) Quantification of (N) TH^+ neurons/total cell count, (O) β-tubulin III^+ neurons/total cell count, and (P) TH^+ neurons/β-tubulin III^+ neurons after differentiating hVM1-Bcl-X_L NSCs co-cultured with murine $CD11c^+$ or $CD11c^-$ neonatal p. microglia. Control, n = 17, N = 6; $CD11c^+$ *neo*. p. microglia, n = 10, N = 4, $CD11c^-$ *neo*. p. microglia, n = 10, N = 4. Mean ± SEM. One-way ANOVA, Dunnett's multiple comparison test with reference to control. *p < 0.05, **p < 0.01, ***p < 0.001. See Figures S2–S4 for additional data.

Microglial Co-culture Enhances Dopaminergic Neuronal Neurite Length, Inhibits Proliferation, and Improves Survival of Differentiating NSCs

To investigate the effect of microglia on neuronal maturation, we quantified in 25-day-old iPSC-NSC-derived cultures the number of microtubule-associated protein 2 $(MAP2)^+$ mature neurons, which was unaffected by BV2 co-culture (Figures 5A and 5B). However, morphological analysis in 14-day-old hNS1 cultures showed that the mature TH^+ neurons in the co-culture group had significantly increased neurite length compared with controls, suggesting a more mature dopaminergic neuronal phenotype (Figures 5C and 5D). Neurite numbers and numbers of neurite branches per neurite were unchanged (Figures 5E and 5F). Collectively, these data suggest that the microglia do not directly influence the number of mature neurons generated, but rather the maturation of the dopaminergic neuronal morphology.

As the total cell count upon co-culture differentiation was markedly increased for both the hNS1 and iPSC-NSCs, but not for the hVM1-Bcl-X_L cells (Figure 4C), we investigated when this increase in total cell count appeared

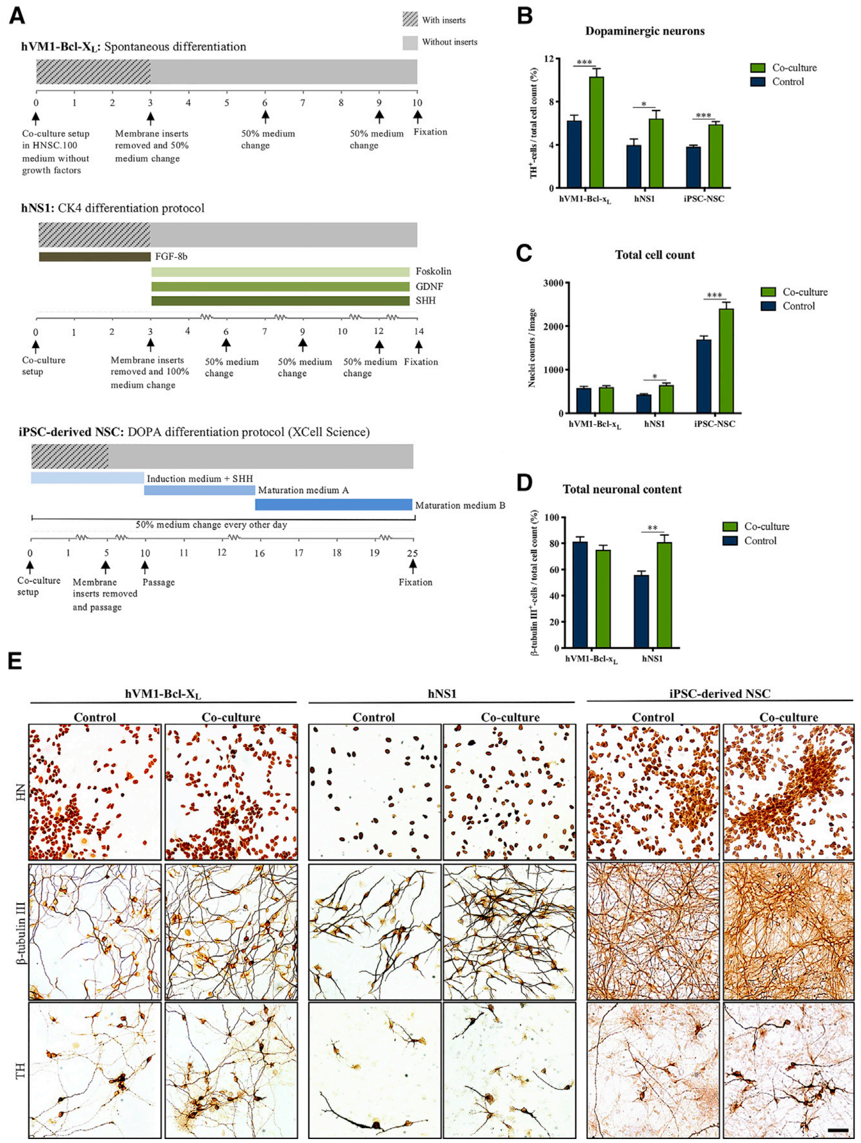

Figure 4. Microglia Co-culture Consistently Increases Dopaminergic Differentiation of Different NSC Lines

(A) Graphical representation of the three different dopaminergic differentiation protocols applied for the hVM1-Bcl-X_L, hNS1, and iPSC-derived NSCs and the period of BV2 microglia co-culturing.

(B–E) Effect of BV2 microglia co-culture differentiation on all three NSC lines as measured by (B and E) TH^+ neurons/total cell count (C and E) total cell count and (D and E) β-tubulin III^+ neurons/total cell count. Scale bar: 50 μm. Multiple t test, Holm-Sidak's multiple comparison test. HVM1-Bcl-X_L: control, n = 15, N = 5; co-culture, n = 15, N = 5. hNS1: control, n = 8, N = 2; co-culture, n = 8, N = 2. iPSC-NSCs: control, n = 17, N = 4; co-culture, n = 15, N = 4. Mean ± SEM. *$p < 0.05$, **$p < 0.01$, ***$p < 0.001$.

during differentiation of the iPSC-NSCs in co-culture with BV2 cells. At days 5 and 10, total cell counts did not differ between controls and co-cultures, but, at day 25, co-cultures displayed significantly increased cell numbers (Figure 5G). As this could either be due to increased proliferation and/or decreased cell death in the cultures, these parameters were investigated during the differentiation of the iPSC-NSCs. The proliferation marker Ki67 was slightly, but significantly, reduced in the co-cultures at day 5, but unchanged at later time points (Figures 5H and 5I). Immunostaining for the apoptotic marker cleaved caspase 3 combined with morphological analysis of fragmented $DAPI^+$ nuclei revealed a significant reduction of apoptotic cell death in the co-cultures at all time points investigated (Figures 5I and 5J). Investigation of necrotic cell death by measuring lactate dehydrogenase release and by counting pyknotic $DAPI^+$ nuclei revealed that necrotic cell death was significantly reduced in the co-culture group at day 25 (Figure 5K).

Dopaminergic Neurogenic Effect of Microglia Is Independent of The Microglial Activation State and Possibly Mediated by Release of TNFα, IL-1β, and IGF1

To investigate if the neurogenic effect of microglia depended on their activation state (Butovsky et al., 2006; Yuan et al., 2017), BV2 microglia were either stimulated with 100 ng/mL LPS or 20 ng/mL IL-4 for 24 h prior to co-culture with hVM1-Bcl-X_L cells (Figure 6A) (Kobayashi et al., 2013). Microglia activation was confirmed by cytokine profiling of conditioned culture medium collected

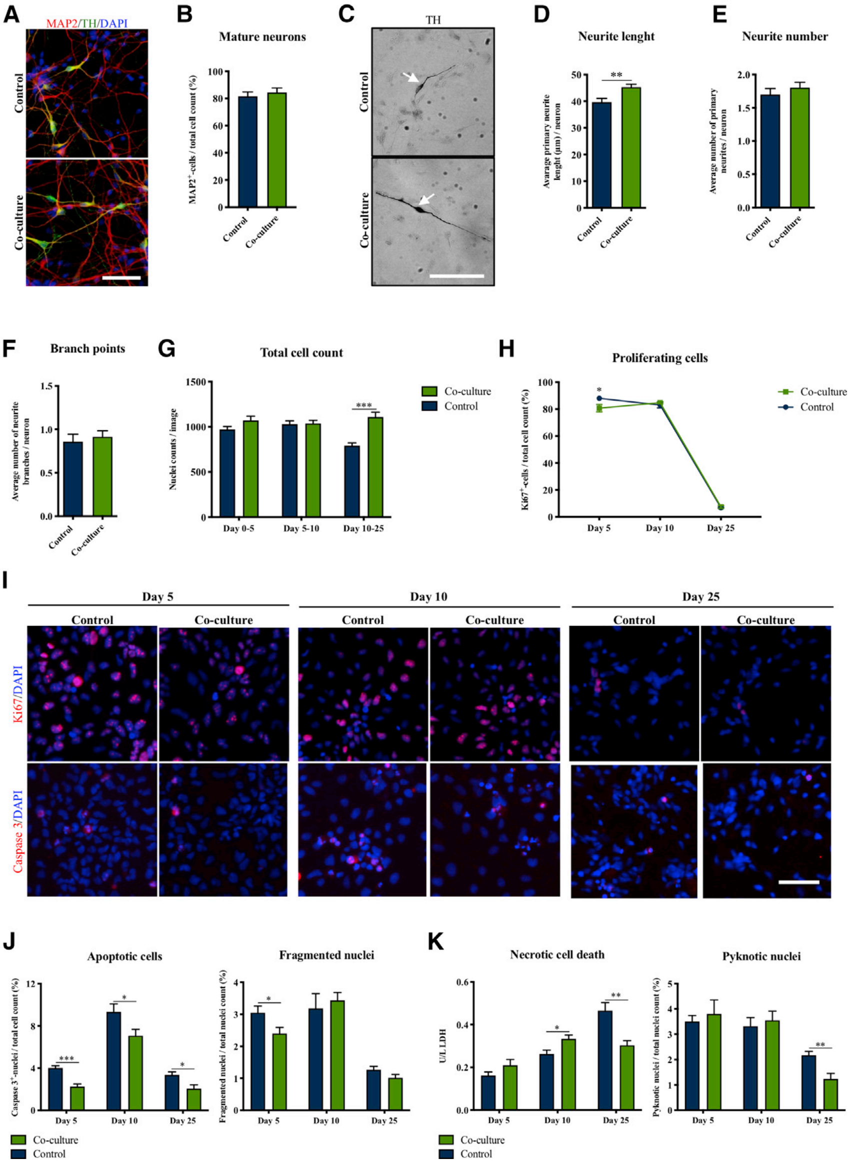

Figure 5. Microglia Co-culture Affects Neurite Outgrowth, Proliferation, and Cell Death of NSCs

(A and B) Immunofluorescence staining and quantification of iPSC-NSC/BV2 co-culture and control differentiated cultures for the mature pan neuronal marker microtubule-associated protein (MAP2)/total cell count. Scale bar: 50 μm. Student's t test. Control, n = 7, N = 3; co-culture, n = 7, N = 3.

(C–F) Morphological analysis of TH^+ neurons in hNS1 NSC/BV2 co-culture assessing (D) neurite length, (E) neurite number, and (F) number of neurite branch points. Scale bar: 100 μm. Student's t test. Control, n = 8, N = 2; co-culture, n = 8, N = 2.

(G) Total cell counts at days 5, 10, and 25 during differentiation of iPSC-NSCs in co-culture with BV2. Multiple t test, Holm-Sidak's multiple comparison test. Days 0–5 and 5–10: control, n = 6, N = 2; co-culture, n = 6, N = 2. Days 10–25: control, n = 19, N = 4; co-culture, n = 14, N = 4.

(H–J) (H and I) Immunofluorescence staining and quantification of iPSC-NSCs for the proliferation marker Ki67 at days 5, 10, and 25 during differentiation. Scale bar: 50 μm. Multiple t tests, Holm-Sidak's multiple comparison test. Day 5 and 10: control, n = 6, N = 2; co-culture, n = 6, N = 2. Day 25: control, n = 9, N = 4; co-culture, n = 7, N = 4. (I and J) Apoptotic cell death in iPSC-NSC/BV2 co-culture at days 5, 10, and 25 during differentiation measured by immunofluorescence staining and quantification of cleaved caspase 3^+ nuclei/total cell count and fragmented nuclei/total cell count. Multiple t test, Holm-Sidak's multiple comparison test. Days 5, 10, and 25: control, n = 6, N = 2; co-culture, n = 6, N = 2.

(K) Necrotic cell death in iPSC-NSC/BV2 co-culture at days 5, 10, and 25 during differentiation measured by lactate dehydrogenase (LDH) release to the medium and pyknotic nuclei/total cell count. Multiple t test, Holm-Sidak's multiple comparison test. Day 5: control, n = 6, N = 2; co-culture; n = 6, N = 2. Days 10 and 25: control, n = 12, N = 4; co-culture, n = 12, N = 4. Mean ± SEM. $^*p < 0.05$, $^{**}p < 0.01$, $^{***}p < 0.001$.

24 h after stimulation (for dose-response data see Figure S5A). Compared with untreated BV2 microglia, LPS-stimulated BV2 microglia displayed a more ramified morphology (Figure 6B) and significantly increased secretion of the pro-inflammatory cytokines TNFα, IL-1β, IL-2, IL-5, IL-6, KC/GROα, IL-12p70, but also IL-10 (Figure 6C). IL-4-stimulated microglia displayed a bipolar morphology (Figure 6B) and decreased their secretion of TNFα compared with untreated BV2 microglia while increasing their secretion of IL-10 and IL-5. IL-4-stimulated microglia were also found to increase their secretion of IL-1β, IL-2, IL-6, KC/GROα, and IL-12p70, but to a significantly lesser extent than the LPS-stimulated microglia (Figure 6C). Importantly, IL-4-stimulated BV2 microglial cells markedly increased their secretion of IGF1 compared with untreated BV2 and LPS-activated BV2 microglia (Figure 6D). Altogether, these secretome analyses confirmed a pro-inflammatory activation upon LPS stimulation of the BV2 microglia and a more anti-inflammatory activation upon IL-4 stimulation.

Subsequent evaluation of the activated BV2 microglia's effect on the TH^+ neuronal yield following NSC

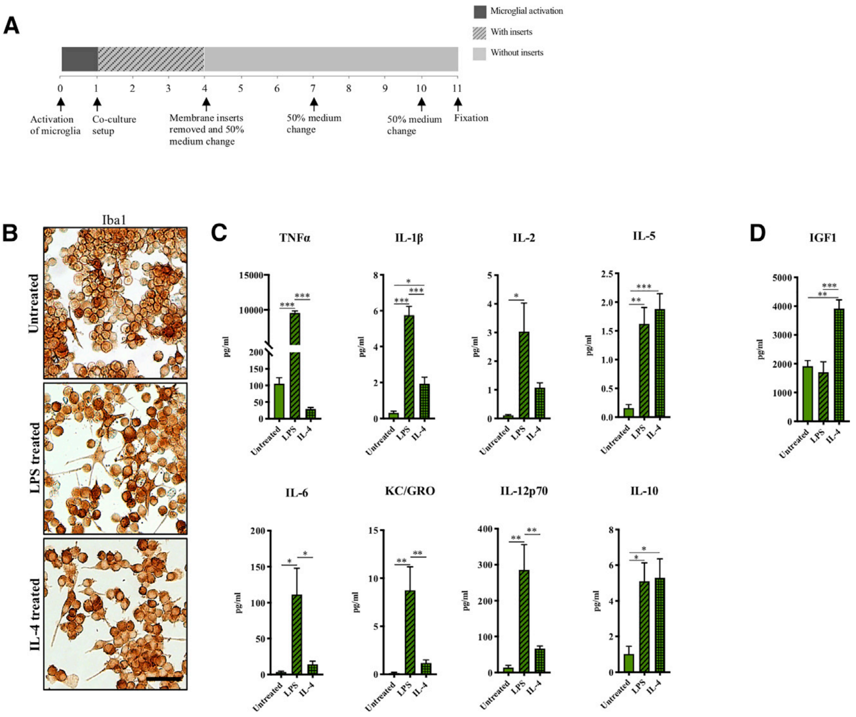

Figure 6. Microglial Cells Treated with LPS and IL-4 show Pro- and Anti-inflammatory Activation Profiles, Respectively

(A) BV2 microglia were activated with 100 ng/mL LPS or 20 ng/mL IL-4 for 24 h prior to co-culture with hVM1-Bcl-X_L cells.

(B) Immunocytochemical staining of activated BV2 microglia for the microglial marker Iba1. Scale bar: 100 μm.

(C) Cytokine profiling of microglia medium after 24 h of activation. Untreated, n = 5, N = 3; LPS, n = 7, N = 4; IL-4, n = 7, N = 4.

(D) ELISA for IGF1 of microglia medium at 24 h post activation. Untreated, n = 4, N = 3; LPS, n = 6, N = 4; IL-4, n = 6, N = 4. One-way ANOVA, Tukey's multiple comparison test. Mean ± SEM. $*p < 0.05$, $**p < 0.01$, $***p < 0.001$.

See Figures S5 and S7 for additional data.

differentiation revealed that neither pro- nor anti-inflammatory activation had changed the positive effect of the BV2 microglia (Figures 7A–7C, S5B, and S5C). This could suggest that co-culturing with NSCs had altered the microglial secretome independently of the prior microglial activation state. Cytokine profiling of the conditioned medium, collected after 3 days of co-culture, showed that the microglial secretome was altered upon co-culturing (Figures 7E and S6), but the secretome of carry-on cultures also revealed that the degree of activation was reduced over time (Figure S7). Medium from all three co-culture groups contained similar levels of TNFα (145–180 pg/mL) and IL-1β (0.5–0.8 pg/mL), which were significantly increased compared with the control group. IL-2, IL-4, IL-5, IL-6, and KC/GROα were also detected in the culture medium but varied between the groups. As the effect on the dopaminergic differentiation of the NSCs was similar for untreated and stimulated BV2 microglia, TNFα and IL-1β could be the potential mediators. When the same medium was analyzed for IGF1 using ELISA, we saw a high content (1,000–5,000 pg/mL) for all co-culture groups compared with a non-detectable level in conditioned medium from controls (Figure 7D). Although the IGF1 content in the media from the LPS-activated BV2 microglia co-culture group was significantly lower than that of the untreated BV2 and IL-4-treated BV2 co-culture groups, the presence of high levels of IGF1 in all co-culture groups suggested that IGF1 was also a potential mediator.

We next investigated if direct addition of recombinant TNFα, IL-1β and IGF1 could increase the dopaminergic differentiation. Three concentrations matching the levels detected in the conditioned culture medium and two concentrations reflecting existing literature (Doherty, 2007; Ling et al., 1998; Supeno et al., 2013) were added to differentiating hVM1-Bcl-X_L cells. Both TNFα and IL-1β increased the yield of TH^+ neurons, whereas IGF1 had no significant effect (Figures 7F–7H). The most efficient concentrations of TNFα, IL-1β, and IGF1 were also tested on the iPSC-derived NSC line XCL1, which confirmed the positive effect of TNFα and IL-1β, but also IGF1 was found to increase the yield of TH^+ neurons from this cell line (Figure 7I).

DISCUSSION

In the present study, we have shown that co-culture of differentiating human NSCs with microglia stimulates dopaminergic differentiation, an effect that was consistent across different types of microglial and NSC lines (Table S1).

The direct co-culture, allowing physical cell-cell contact, was impaired by microglial overgrowth, so, based on both cell morphology and yield of TH^+ neurons, the indirect co-culture using semi-porous membrane inserts was found to be optimal. As these membranes allow only diffusible, non-physical interactions between the NSCs and microglia, secreted factors were likely responsible for the

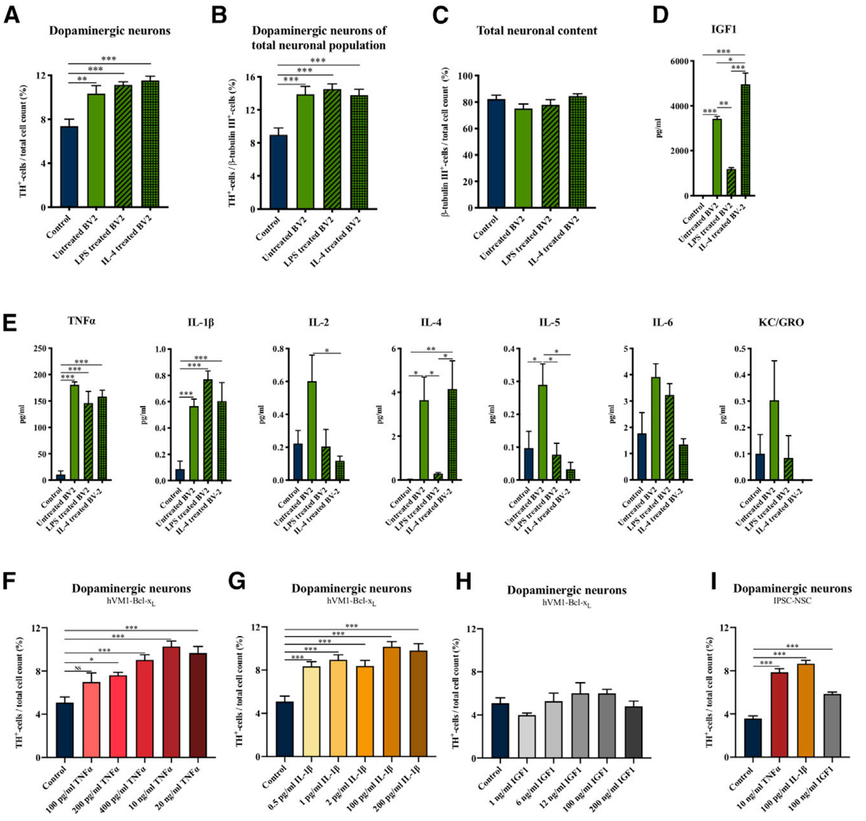

Figure 7. Increased Dopaminergic Differentiation of NSCs Independent on Prior Microglial Activation and Identification of TNFα, IL-1β, and IGF1 as Potential Mediators

(A–C) Immunocytochemical staining and quantification of hVM1-Bcl-X_L NSC-derived neurons after co-culture differentiation with pro- and anti-inflammatory activated BV2 microglia for (A) TH^+ neurons/total cell count, (B) TH^+ neurons/β-tubulin III^+ neurons, and (C) β-tubulin III^+ neurons/total cell count. One-way ANOVA, Dunnett's multiple comparison test with reference to control. Control, n = 20, N = 7; untreated BV2, n = 15, N = 5; LPS-treated BV2, n = 10, N = 4; IL-4 treated BV2, n = 12, N = 5.

(D) IGF1 ELISA; all groups, n = 4, N = 2, and (E) cytokine profiling; control, n = 6, N = 3; untreated BV2, LPS-treated BV2, and IL-4 treated BV2, n = 4, N = 2, of co-culture medium collected at day 3 of differentiation. One-way ANOVA, Tukey's multiple comparison test.

(F–H) Effect on TH^+ neurons/total cell count of direct addition of recombinant human (F) TNFα, (G) IL-1β, and (H) IGF1 to hVM1-Bcl-X_L NSCs during differentiation. One-way ANOVA, Dunnett's multiple comparison test with reference to control. All groups, n = 6, N = 2.

(I) Most efficient concentrations of recombinant TNFα, IL-1β, and IGF1 tested on iPSC-NSCs. One-way ANOVA, Dunnett's multiple comparison test with reference to control. All groups, n = 6, N = 2. Mean ± SEM. *$p < 0.05$, **$p < 0.01$, ***$p < 0.001$, NS = not significant. See Figures S6 and S7 for additional data.

observed increase in dopaminergic content. The positive effect of microglia co-culture on dopaminergic differentiation was supported by qRT-PCR, revealing increased expression of general dopaminergic markers and midbrain-specific transcription factors, and co-expression of the floorplate marker FOXA2 in TH^+ neurons. Previous studies have confirmed that all the applied NSC lines (hVM1-Bcl-X_L, hNS,1 and iPSC-NSC [XCL1]) generate TH^+ neurons of the midbrain dopaminergic phenotype (Bogetofte et al., 2019; Liste et al., 2004; Okarmus et al., 2020; Seiz et al., 2012).

Although the positive effect on dopaminergic differentiation was consistent across the investigated microglia and NSC lines, the effect on the general neurogenesis varied. For the hVM1-Bcl-X_L cells, CD11c$^+$ neonatal primary microglia increased the percentage of β-tubulin III^+-neurons, whereas no difference was found for unsorted neonatal primary microglia, and BV2 and CHME microglia. The increased dopaminergic differentiation caused by CHME and BV2 microglia was possibly at the expense of GABAergic differentiation, resulting in an unchanged general neurogenesis. This could suggest a difference between microglia cell lines in their effect on the differentiation of other neuronal subtypes than the dopaminergic, but the general neurogenic effect was also found to vary between the NSC lines. Differentiating hNS1 in co-culture with BV2 increased the percentage of β-tubulin III^+-neurons, and a similar trend was observed for the iPSC-NSCs (not quantified). This difference might be explained by overexpression of the anti-apoptotic protein Bcl-X_L in the hVM1-Bcl-X_L line as the effect of the microglia was partly apoptosis mediated.

Primary microglia isolated from adult mice failed to increase the dopaminergic differentiation of the NSCs. Adult and neonatal microglia have recently been shown to have very different gene expression profiles (Matcovitch-Natan et al., 2016; Wlodarczyk et al., 2017), and, as both CHME and BV2 microglia cell lines have been produced from neonatal microglia (Blasi et al., 1990; Janabi et al., 1995), our findings suggest that the dopaminergic neurogenic effect of microglia is restricted to microglia of embryonic origin. Our secretome comparison between the different microglia cells revealed secretion of all investigated cytokines; however, adult and primary microglia generally

secreted higher levels than BV2 and CHME. It can be speculated whether this is due to the isolation and sorting of the primary microglia prior to *in vitro* culturing, as such procedures have been shown to induce transient activation of microglia (Bohlen et al., 2019).

As microglia have been reported to play a variety of roles during brain development (Menassa and Gomez-Nicola, 2018), we investigated their effect on neuronal maturation, morphological development, proliferation, and cell death during dopaminergic differentiation of the NSCs. Although no apparent effect on the number of $MAP2^+$ mature neurons was observed, the dopaminergic neurons displayed a more mature morphology with increased neurite length. This is in line with previous studies on human fetal tissue where microglia were associated with axonal tracts during development (Monier et al., 2007; Verney et al., 2010) and microglial deficiency caused aberrant dopaminergic axonal outgrowth in mouse embryos (Squarzoni et al., 2014), suggesting a role of microglia in axonal development. A slight decline in proliferation and reduced apoptosis was observed at the beginning of differentiation in the presence of the microglia, suggesting neurotrophic support from microglia. Apoptotic and necrotic cell death was reduced at the end of differentiation when microglia were no longer present. As differentiating the NSCs in co-culture with microglia also increased the number of astrocytes, the reduced cell death at the end of differentiation may have been due to microglia-astrocyte crosstalk leading to increased neurotrophic support. The total cell count upon co-culture differentiation was markedly increased for both the hNS1 and iPSC-NSC cell lines but not for the hVM1-Bcl-X_L cells. It is possible that the overexpression of the anti-apoptotic protein Bcl-X_L in this cell line may have masked the anti-apoptotic effect of the microglia secretome.

Although previous studies have reported a selective neurogenic effect of anti-inflammatory activated microglia (Butovsky et al., 2006; Yuan et al., 2017), we found that the dopaminergic neurogenic effect on NSCs was independent of prior microglial activation as both untreated, pro-, and anti-inflammatory activated BV2 microglia increased the yields of dopaminergic neurons. In line with this, cytokine profiling of the conditioned co-culture media at day 3 of differentiation showed very different profiles for untreated, LPS-treated, and IL-4-treated BV2 groups compared with their cytokine release prior to co-culture, suggesting that NSC-microglia crosstalk changes the microglial secretome toward a neurogenic profile independent of the prior activation state. This was supported by secretome analysis comparing co-cultures with microglia carry-on monocultures at day 3, which revealed higher cytokine levels in medium from co-cultures, especially of TNFα and IL-1β, that were unaffected by prior activation. It was, however, also shown from the carry-on microglia that the degree of activation was reduced over time. In comparison, a previous study showed that iPSC-derived microglial progenitors activated with LPS displayed a more anti-inflammatory cytokine response when co-cultured with iPSC-derived cortical neuronal progenitors compared with corresponding monocultures (Haenseler et al., 2017). Furthermore, activation of embryonic microglia has previously been reported not to alter the increased survival of embryonic dopaminergic neurons observed in a primary co-culture cell model (Zietlow et al., 1999). NSC-microglia crosstalk is therefore most likely playing a role.

TNFα, IL-1β, and IGF1 were the only factors found at similar levels in the media for all co-culture groups, suggesting that they might be responsible for the increased dopaminergic neurogenesis. Embryonic microglia are known to secrete cytokines, including IL-1β and TNFα (Deverman and Patterson, 2009; Giulian et al., 1988; Munoz-Fernandez and Fresno, 1998), and IGF1 (Wlodarczyk et al., 2017). IL-1β is a recognized mitogen for astrocytes (Giulian et al., 1988; Wang et al., 2007) and has been found to increase astrogliogenesis while decreasing neurogenesis of hippocampal neural progenitor cells (NPCs) (Chen et al., 2013; Koo and Duman, 2008; Wang et al., 2007; Zunszain et al., 2012), but recombinant IL-1β has also been shown to enhance differentiation of mesencephalic NPCs into dopaminergic neurons (Ling et al., 1998). The IL-1β concentration detected in our conditioned co-culture medium was very low (0.5–0.8 pg/mL), but treatment of differentiating hVM1-Bcl-X_L NSCs with both low and high concentrations of recombinant human IL-1β increased the yield of dopaminergic neurons.

IGF1 exerts pleiotropic effects during embryogenesis and in adulthood, including NSC proliferation, astrogliogenesis, and neuronal survival, differentiation, and maturation (Nieto-Estevez et al., 2016). Microglia-induced neurogenesis in the early postnatal subventricular zone is not mediated through the release of IGF1 from microglia, however, but rather their release of IL-1β, IL-6, TNFα, and IFN-γ (Shigemoto-Mogami et al., 2014). To our knowledge, no previous studies have linked IGF1 with selective dopaminergic neurogenesis, and we found that $CD11c^+$ and $CD11c^-$ neonatal primary microglia increased the yields of dopaminergic neurons to a similar extent, although $CD11C^+$ microglia express much higher levels of IGF1 (Wlodarczyk et al., 2017). This suggests that IGF1 alone is not responsible for the increased dopaminergic differentiation observed in our experiments. Direct addition of recombinant human IGF1 to differentiating hVM1-Bcl-X_L did not affect the resulting number of dopaminergic neurons.

TNFα is an important regulator of developmental apoptosis and synaptogenesis (Mosser et al., 2017), but it is also implicated in the pathogenesis of neurodegenerative

conditions such as Parkinson disease (Mogi et al., 1994, 2000). It has previously been reported that exposure of E12.5 mouse ventral mesencephalic dopaminergic neurons to TNFα increased the number of dopaminergic neurons, while TNFα-treated cultures from E14-E16 mice displayed a decreased number of dopaminergic neurons due to apoptotic cell death (Doherty, 2007). This suggests that developing ventral mesencephalic dopaminergic neurons switch their response to TNFα from neurotrophic to neurotoxic as they mature. In accordance with this hypothesis, treatment of hVM1-Bcl-X_L NSCs with recombinant human TNFα was found to stimulate dopaminergic neuronal differentiation. Testing the effect of recombinant TNFα, IL-1β, and IGF-1 on another NSC line, iPSC-derived NSCs, revealed that all factors had a positive effect on dopaminergic differentiation, suggesting that IGF1, under certain circumstances, may also exert a positive role.

This is the first study to comprehensively demonstrate that co-culturing of human NSCs with microglia during differentiation enhances the yield of dopaminergic neurons. The effect was found to be consistent across different NSC lines but was restricted to microglia of embryonic origin. Pre-activation of the microglia did not change the positive effect, and TNFα, IL-1β, and IGF-1 were identified as potential key mediators. We provide evidence that the effect is mediated through reduced proliferation and decreased apoptotic/necrotic cell death taking place in a sequential manner during the differentiation process. These findings indicate an instructive role of microglia on dopaminergic neurogenesis and may provide new insights into potential inductive and protective factors that can improve *in vitro* derivation of human dopaminergic neurons.

EXPERIMENTAL PROCEDURES

See Supplemental Information for detailed methods.

Human NSC Lines

Cell isolation, genetic modification, and general characterization of the somatic human NSC lines hVM1-Bcl-X_L (ventral mesencephalic NSC line) and hNS1 (forebrain NSC line) are described elsewhere (Courtois et al., 2010; Krabbe et al., 2009, 2014; Villa et al., 2000, 2004, 2009). Propagation and dopaminergic differentiation were performed as previously described (Krabbe et al., 2009, 2014). The human iPSC-derived NSC line (XCL1) was obtained from XCell Science Inc. (Novato, CA) (Swistowski et al., 2010) and propagated according to standard protocols provided by the manufacturer. See Supplemental information for detailed procedures.

Microglia Cell Lines

Establishments of the mouse microglia cell line BV2 and the human microglia cell line CHME are described elsewhere (Blasi et al., 1990; Janabi et al., 1995). Propagation methods are described in Supplemental information.

Primary microglia were isolated from the CNS of P3-P5 C57BL/6j mice and from adult (8–10 weeks) C57BL/6j mice (Wlodarczyk et al., 2014). Cell isolation and sorting are described in Supplemental information.

Co-culture Procedures

NSCs were exposed to microglia-secreted factors during differentiation by three different co-culture setups: exposure to BV2 microglia-conditioned media, direct co-culture, or co-culture using semi-porous membrane inserts (Figure 1A).

Microglia-conditioned medium was generated by culturing BV2 microglia in HNSC.100 medium for 3 days and collecting the medium through a 0.22-μm filter (Sarstedt) before mixing 1:1 with new unconditioned medium. hVM1-Bcl-X_L cells were seeded in this medium at day 0 of differentiation. One-hundred percent media change was performed every third day using newly prepared conditioned medium mixed in the same way. The control group was differentiated in unconditioned HNSC.100 medium.

For direct co-culture, hVM1-Bcl-X_L cells were seeded together with BV2 microglia cells in a 1:50 ratio, and differentiation was performed as described in the Supplemental information. The control group did not contain any microglia cells.

For co-culture using semi-porous membrane inserts (Merck), NSCs were seeded and allowed to attach for half an hour before inserts were placed and microglia seeded on the inserts in a 1:5 ratio relative to the NSCs. Differentiation was performed as described in the Supplemental information, and inserts were removed at day 3 for hVM1-Bcl-X_L and hNS1 differentiations and at day 5 for iPSC-NSC differentiation. The control groups received empty inserts.

Immunocytochemistry, Bioimaging and Western Blotting

Immunocytochemistry, image analysis, and western blotting were performed as described in Supplemental information.

Microglial Activation and Cytokine Profiling

BV2 microglia were activated with either 100 ng/mL LPS from *Escherichia coli* O111:B4 (Sigma) or 20 ng/mL IL-4 (Peprotech) for 24 h in serum-free RPMI-1640 medium prior to co-culture setup (Kobayashi et al., 2013). Activation was confirmed by cytokine profiling using the V-PLEX Proinflammatoy Panel 1 Mouse kit (MesoScale Discovery) and IGF1 ELISA (Sigma) according to the manufacturers' instructions. The same MesoScale kit and IGF1 ELISA were used to screen the conditioned co-culture media at day 3 of differentiation and for carry-on microglia cultures.

TNFα, IL-1β, and IGF-1 Treatments

hVM1-Bcl-X_L cells and iPSC-NSCs were differentiated with addition of human recombinant TNFα (Sigma), IL-1β (R&D), or IGF-1 (R&D) during the first part of differentiation. Details are provided in Supplemental information.

Statistical Analysis

Analysis was performed in GraphPad Prism version 6.0 (GraphPad Software, United States) using two-tailed unpaired Student's t test, multiple t test, one-way ANOVA, or two-way ANOVA as

appropriate. Data are presented as mean ± SEM, and p values < 0.05 were considered statistically significant.

SUPPLEMENTAL INFORMATION

Supplemental Information can be found online at https://doi.org/10.1016/j.stemcr.2020.12.011.

AUTHOR CONTRIBUTIONS

Conceptualization, S.I.S., H.B., and M.M.; Methodology and Investigation, S.I.S., H.B., L.R., J.B.A., D.H., A.A., A.W., S.G.L., M.J., and J.O.; Resources, A.M.S., M.D.S., B.W.K., T.O., and M.M.; Data Curation, S.I.S; Writing – Original Draft, S.I.S., H.B., K.F., and M.M.; Writing – Review & Editing, all authors; Funding Acquisition, T.O., A.M.S. and M.M.

ACKNOWLEDGMENTS

We thank Dorte Lyholmer, Nadine Becker-von Buch, and Ulla Damgaard Munk for excellent technical assistance and Claire Gudex for proofreading the manuscript. This work was supported by the Innovation Fund Denmark (BrainStem; www.brainstem.dk), the Lundbeck Foundation, the Danish Parkinson Foundation, the Jascha Foundation, IMK Almene Fond, the A.P. Møller Foundation, and the Faculty of Health Sciences, University of Southern Denmark. Work at the CBMSO was supported by grants (to A.M.S.) SAF-2017-83241-R, RETICS TerCel RD16/0011/0032.

Received: January 16, 2020
Revised: December 17, 2020
Accepted: December 18, 2020
Published: January 21, 2021

REFERENCES

Blasi, E., Barluzzi, R., Bocchini, V., Mazzolla, R., and Bistoni, F. (1990). Immortalization of murine microglial cells by a v-raf/v-myc carrying retrovirus. J. Neuroimmunol. *27*, 229–237.

Bogetofte, H., Jensen, P., Ryding, M., Schmidt, S.I., Okarmus, J., Ritter, L., Worm, C.S., Hohnholt, M.C., Azevedo, C., Roybon, L., et al. (2019). PARK2 mutation causes metabolic disturbances and impaired survival of human iPSC-derived neurons. Front. Cell Neurosci. *13*, 297.

Bohlen, C.J., Bennett, F.C., and Bennett, M.L. (2019). Isolation and culture of microglia. Curr. Protoc. Immunol. *125*, e70.

Butovsky, O., Ziv, Y., Schwartz, A., Landa, G., Talpalar, A.E., Pluchino, S., Martino, G., and Schwartz, M. (2006). Microglia activated by IL-4 or IFN-gamma differentially induce neurogenesis and oligodendrogenesis from adult stem/progenitor cells. Mol. Cell Neurosci. *31*, 149–160.

Chen, E., Xu, D., Lan, X., Jia, B., Sun, L., Zheng, J.C., and Peng, H. (2013). A novel role of the STAT3 pathway in brain inflammation-induced human neural progenitor cell differentiation. Curr. Mol. Med. *13*, 1474–1484.

Courtois, E.T., Castillo, C.G., Seiz, E.G., Ramos, M., Bueno, C., Liste, I., and Martinez-Serrano, A. (2010). In vitro and in vivo enhanced generation of human A9 dopamine neurons from neural stem cells by Bcl-XL. J. Biol. Chem. *285*, 9881–9897.

Cunningham, C.L., Martinez-Cerdeno, V., and Noctor, S.C. (2013). Microglia regulate the number of neural precursor cells in the developing cerebral cortex. J. Neurosci. *33*, 4216–4233.

De Biase, L.M., Schuebel, K.E., Fusfeld, Z.H., Jair, K., Hawes, I.A., Cimbro, R., Zhang, H.Y., Liu, Q.R., Shen, H., Xi, Z.X., et al. (2017). Local cues establish and maintain region-specific phenotypes of basal ganglia microglia. Neuron *95*, 341–356 e346.

Deverman, B.E., and Patterson, P.H. (2009). Cytokines and CNS development. Neuron *64*, 61–78.

Doherty, G.H. (2007). Developmental switch in the effects of TNFalpha on ventral midbrain dopaminergic neurons. Neurosci. Res. *57*, 296–305.

Ekdahl, C.T., Claasen, J.H., Bonde, S., Kokaia, Z., and Lindvall, O. (2003). Inflammation is detrimental for neurogenesis in adult brain. Proc. Natl. Acad. Sci. U S A *100*, 13632–13637.

Ekdahl, C.T., Kokaia, Z., and Lindvall, O. (2009). Brain inflammation and adult neurogenesis: the dual role of microglia. Neuroscience *158*, 1021–1029.

Elmore, M.R., Najafi, A.R., Koike, M.A., Dagher, N.N., Spangenberg, E.E., Rice, R.A., Kitazawa, M., Matusow, B., Nguyen, H., West, B.L., et al. (2014). Colony-stimulating factor 1 receptor signaling is necessary for microglia viability, unmasking a microglia progenitor cell in the adult brain. Neuron *82*, 380–397.

Franco, R., and Fernandez-Suarez, D. (2015). Alternatively activated microglia and macrophages in the central nervous system. Prog. Neurobiol. *131*, 65–86.

Giulian, D., Young, D.G., Woodward, J., Brown, D.C., and Lachman, L.B. (1988). Interleukin-1 is an astroglial growth factor in the developing brain. J. Neurosci. *8*, 709–714.

Haenseler, W., Sansom, S.N., Buchrieser, J., Newey, S.E., Moore, C.S., Nicholls, F.J., Chintawar, S., Schnell, C., Antel, J.P., Allen, N.D., et al. (2017). A highly efficient human pluripotent stem cell microglia model displays a neuronal-co-culture-specific expression profile and inflammatory response. Stem Cell Reports *8*, 1727–1742.

Janabi, N., Peudenier, S., Heron, B., Ng, K.H., and Tardieu, M. (1995). Establishment of human microglial cell lines after transfection of primary cultures of embryonic microglial cells with the SV40 large T antigen. Neurosci. Lett. *195*, 105–108.

Kobayashi, K., Imagama, S., Ohgomori, T., Hirano, K., Uchimura, K., Sakamoto, K., Hirakawa, A., Takeuchi, H., Suzumura, A., Ishiguro, N., et al. (2013). Minocycline selectively inhibits M1 polarization of microglia. Cell Death Dis. *4*, e525.

Koo, J.W., and Duman, R.S. (2008). IL-1beta is an essential mediator of the antineurogenic and anhedonic effects of stress. Proc. Natl. Acad. Sci. U S A *105*, 751–756.

Krabbe, C., Bak, S.T., Jensen, P., von Linstow, C., Martinez Serrano, A., Hansen, C., and Meyer, M. (2014). Influence of oxygen tension on dopaminergic differentiation of human fetal stem cells of midbrain and forebrain origin. PLoS One *9*, e96465.

Krabbe, C., Courtois, E., Jensen, P., Jorgensen, J.R., Zimmer, J., Martinez-Serrano, A., and Meyer, M. (2009). Enhanced dopaminergic differentiation of human neural stem cells by synergistic effect of

Bcl-xL and reduced oxygen tension. J. Neurochem. *110*, 1908–1920.

Lawson, L.J., Perry, V.H., Dri, P., and Gordon, S. (1990). Heterogeneity in the distribution and morphology of microglia in the normal adult mouse brain. Neuroscience *39*, 151–170.

Ling, Z.D., Potter, E.D., Lipton, J.W., and Carvey, P.M. (1998). Differentiation of mesencephalic progenitor cells into dopaminergic neurons by cytokines. Exp. Neurol. *149*, 411–423.

Liste, I., Garcia-Garcia, E., and Martinez-Serrano, A. (2004). The generation of dopaminergic neurons by human neural stem cells is enhanced by Bcl-XL, both in vitro and in vivo. J. Neurosci. *24*, 10786–10795.

Marin-Teva, J.L., Dusart, I., Colin, C., Gervais, A., van Rooijen, N., and Mallat, M. (2004). Microglia promote the death of developing Purkinje cells. Neuron *41*, 535–547.

Matcovitch-Natan, O., Winter, D.R., Giladi, A., Vargas Aguilar, S., Spinrad, A., Sarrazin, S., Ben-Yehuda, H., David, E., Zelada Gonzalez, F., Perrin, P., et al. (2016). Microglia development follows a stepwise program to regulate brain homeostasis. Science *353*, aad8670.

Menassa, D.A., and Gomez-Nicola, D. (2018). Microglial dynamics during human brain development. Front. Immunol. *9*, 1014.

Miyamoto, A., Wake, H., Ishikawa, A.W., Eto, K., Shibata, K., Murakoshi, H., Koizumi, S., Moorhouse, A.J., Yoshimura, Y., and Nabekura, J. (2016). Microglia contact induces synapse formation in developing somatosensory cortex. Nat. Commun. *7*, 12540.

Mogi, M., Harada, M., Riederer, P., Narabayashi, H., Fujita, K., and Nagatsu, T. (1994). Tumor necrosis factor-alpha (TNF-alpha) increases both in the brain and in the cerebrospinal fluid from parkinsonian patients. Neurosci. Lett. *165*, 208–210.

Mogi, M., Togari, A., Kondo, T., Mizuno, Y., Komure, O., Kuno, S., Ichinose, H., and Nagatsu, T. (2000). Caspase activities and tumor necrosis factor receptor R1 (p55) level are elevated in the substantia nigra from parkinsonian brain. J. Neural Transm. (Vienna) *107*, 335–341.

Monier, A., Adle-Biassette, H., Delezoide, A.L., Evrard, P., Gressens, P., and Verney, C. (2007). Entry and distribution of microglial cells in human embryonic and fetal cerebral cortex. J. Neuropathol. Exp. Neurol. *66*, 372–382.

Monje, M.L., Toda, H., and Palmer, T.D. (2003). Inflammatory blockade restores adult hippocampal neurogenesis. Science *302*, 1760–1765.

Morello, F., and Partanen, J. (2015). Diversity and development of local inhibitory and excitatory neurons associated with dopaminergic nuclei. FEBS Lett. *589*, 3693–3701.

Mosher, K.I., Andres, R.H., Fukuhara, T., Bieri, G., Hasegawa-Moriyama, M., He, Y., Guzman, R., and Wyss-Coray, T. (2012). Neural progenitor cells regulate microglia functions and activity. Nat. Neurosci. *15*, 1485–1487.

Mosser, C.A., Baptista, S., Arnoux, I., and Audinat, E. (2017). Microglia in CNS development: shaping the brain for the future. Prog. Neurobiol. *149-150*, 1–20.

Munoz-Fernandez, M.A., and Fresno, M. (1998). The role of tumour necrosis factor, interleukin 6, interferon-gamma and inducible nitric oxide synthase in the development and pathology of the nervous system. Prog. Neurobiol. *56*, 307–340.

Nieto-Estevez, V., Defterali, C., and Vicario-Abejon, C. (2016). IGF-I: a key growth factor that regulates neurogenesis and synaptogenesis from embryonic to adult stages of the brain. Front. Neurosci. *10*, 52.

Okarmus, J., Bogetofte, H., Schmidt, S.I., Ryding, M., Garcia-Lopez, S., Ryan, B.J., Martinez-Serrano, A., Hyttel, P., and Meyer, M. (2020). Lysosomal perturbations in human dopaminergic neurons derived from induced pluripotent stem cells with PARK2 mutation. Sci. Rep. *10*, 10278.

Paolicelli, R.C., Bolasco, G., Pagani, F., Maggi, L., Scianni, M., Panzanelli, P., Giustetto, M., Ferreira, T.A., Guiducci, E., Dumas, L., et al. (2011). Synaptic pruning by microglia is necessary for normal brain development. Science *333*, 1456–1458.

Parkhurst, C.N., Yang, G., Ninan, I., Savas, J.N., Yates, J.R., 3rd, Lafaille, J.J., Hempstead, B.L., Littman, D.R., and Gan, W.B. (2013). Microglia promote learning-dependent synapse formation through brain-derived neurotrophic factor. Cell *155*, 1596–1609.

Polazzi, E., and Monti, B. (2010). Microglia and neuroprotection: from in vitro studies to therapeutic applications. Prog. Neurobiol. *92*, 293–315.

Pont-Lezica, L., Beumer, W., Colasse, S., Drexhage, H., Versnel, M., and Bessis, A. (2014). Microglia shape corpus callosum axon tract fasciculation: functional impact of prenatal inflammation. Eur. J. Neurosci. *39*, 1551–1557.

Schafer, D.P., Lehrman, E.K., Kautzman, A.G., Koyama, R., Mardinly, A.R., Yamasaki, R., Ransohoff, R.M., Greenberg, M.E., Barres, B.A., and Stevens, B. (2012). Microglia sculpt postnatal neural circuits in an activity and complement-dependent manner. Neuron *74*, 691–705.

Seiz, E.G., Ramos-Gomez, M., Courtois, E.T., Tonnesen, J., Kokaia, M., Liste Noya, I., and Martinez-Serrano, A. (2012). Human midbrain precursors activate the expected developmental genetic program and differentiate long-term to functional A9 dopamine neurons in vitro. Enhancement by Bcl-X(L). Exp. Cell Res. *318*, 2446–2459.

Shigemoto-Mogami, Y., Hoshikawa, K., Goldman, J.E., Sekino, Y., and Sato, K. (2014). Microglia enhance neurogenesis and oligodendrogenesis in the early postnatal subventricular zone. J. Neurosci. *34*, 2231–2243.

Squarzoni, P., Oller, G., Hoeffel, G., Pont-Lezica, L., Rostaing, P., Low, D., Bessis, A., Ginhoux, F., and Garel, S. (2014). Microglia modulate wiring of the embryonic forebrain. Cell Rep. *8*, 1271–1279.

Su, P., Zhang, J., Zhao, F., Aschner, M., Chen, J., and Luo, W. (2014). The interaction between microglia and neural stem/precursor cells. Brain Res. Bull. *109*, 32–38.

Supeno, N.E., Pati, S., Hadi, R.A., Ghani, A.R., Mustafa, Z., Abdullah, J.M., Idris, F.M., Han, X., and Jaafar, H. (2013). IGF-1 acts as controlling switch for long-term proliferation and maintenance of EGF/FGF-responsive striatal neural stem cells. Int. J. Med. Sci. *10*, 522–531.

Swistowski, A., Peng, J., Liu, Q., Mali, P., Rao, M.S., Cheng, L., and Zeng, X. (2010). Efficient generation of functional dopaminergic

neurons from human induced pluripotent stem cells under defined conditions. Stem Cells *28*, 1893–1904.

Tronnes, A.A., Koschnitzky, J., Daza, R., Hitti, J., Ramirez, J.M., and Hevner, R. (2016). Effects of lipopolysaccharide and progesterone exposures on embryonic cerebral cortex development in mice. Reprod. Sci. *23*, 771–778.

Verney, C., Monier, A., Fallet-Bianco, C., and Gressens, P. (2010). Early microglial colonization of the human forebrain and possible involvement in periventricular white-matter injury of preterm infants. J. Anat. *217*, 436–448.

Villa, A., Liste, I., Courtois, E.T., Seiz, E.G., Ramos, M., Meyer, M., Juliusson, B., Kusk, P., and Martinez-Serrano, A. (2009). Generation and properties of a new human ventral mesencephalic neural stem cell line. Exp. Cell Res. *315*, 1860–1874.

Villa, A., Navarro-Galve, B., Bueno, C., Franco, S., Blasco, M.A., and Martinez-Serrano, A. (2004). Long-term molecular and cellular stability of human neural stem cell lines. Exp. Cell Res. *294*, 559–570.

Villa, A., Snyder, E.Y., Vescovi, A., and Martinez-Serrano, A. (2000). Establishment and properties of a growth factor-dependent, perpetual neural stem cell line from the human CNS. Exp. Neurol. *161*, 67–84.

Wakselman, S., Bechade, C., Roumier, A., Bernard, D., Triller, A., and Bessis, A. (2008). Developmental neuronal death in hippocampus requires the microglial CD11b integrin and DAP12 immunoreceptor. J. Neurosci. *28*, 8138–8143.

Wang, X., Fu, S., Wang, Y., Yu, P., Hu, J., Gu, W., Xu, X.M., and Lu, P. (2007). Interleukin-1beta mediates proliferation and differentiation of multipotent neural precursor cells through the activation of SAPK/JNK pathway. Mol. Cell Neurosci. *36*, 343–354.

Wlodarczyk, A., Holtman, I.R., Krueger, M., Yogev, N., Bruttger, J., Khorooshi, R., Benmamar-Badel, A., de Boer-Bergsma, J.J., Martin, N.A., Karram, K., et al. (2017). A novel microglial subset plays a key role in myelinogenesis in developing brain. EMBO J. *36*, 3292–3308.

Wlodarczyk, A., Lobner, M., Cedile, O., and Owens, T. (2014). Comparison of microglia and infiltrating CD11c(+) cells as antigen presenting cells for T cell proliferation and cytokine response. J. Neuroinflammation *11*, 57.

Yang, F., Liu, Z.R., Chen, J., Zhang, S.J., Quan, Q.Y., Huang, Y.G., and Jiang, W. (2010). Roles of astrocytes and microglia in seizure-induced aberrant neurogenesis in the hippocampus of adult rats. J. Neurosci. Res. *88*, 519–529.

Yuan, J., Ge, H., Liu, W., Zhu, H., Chen, Y., Zhang, X., Yang, Y., Yin, Y., Chen, W., Wu, W., et al. (2017). M2 microglia promotes neurogenesis and oligodendrogenesis from neural stem/progenitor cells via the PPARgamma signaling pathway. Oncotarget *8*, 19855–19865.

Zietlow, R., Dunnett, S.B., and Fawcett, J.W. (1999). The effect of microglia on embryonic dopaminergic neuronal survival in vitro: diffusible signals from neurons and glia change microglia from neurotoxic to neuroprotective. Eur. J. Neurosci. *11*, 1657–1667.

Zunszain, P.A., Anacker, C., Cattaneo, A., Choudhury, S., Musaelyan, K., Myint, A.M., Thuret, S., Price, J., and Pariante, C.M. (2012). Interleukin-1beta: a new regulator of the kynurenine pathway affecting human hippocampal neurogenesis. Neuropsychopharmacology *37*, 939–949.

Stem Cell Reports, Volume *16*

Supplemental Information

Microglia-Secreted Factors Enhance Dopaminergic Differentiation of Tissue- and iPSC-Derived Human Neural Stem Cells

Sissel Ida Schmidt, Helle Bogetofte, Louise Ritter, Jette Bach Agergaard, Ditte Hammerich, Amina Arslanagic Kabiljagic, Agnieszka Wlodarczyk, Silvia Garcia Lopez, Mia Dahl Sørensen, Mie Lærkegård Jørgensen, Justyna Okarmus, Alberto Martínez Serrano, Bjarne Winther Kristensen, Kristine Freude, Trevor Owens, and Morten Meyer

Supplemental Information

Supplemental Table of Contents

Figure S1. Additional data for the different co-culture setups, Related to Figure 1.

Figure S2. Consistent positive effect of BV2 and CHME microglia on dopaminergic differentiation of the iPSC-NSC line XCL-1, Related to Figure 3.

Figure S3. Further characterization of differentiated hVM1-Bcl-X_L/BV2/CHME co-cultures and effects on total cell count for co-culture with different primary microglial cell types, Related to Figure 3.

Figure S4. Secretome comparison between BV2, CHME, adult and neonatal primary microglia, Related to Figure 3.

Figure S5. Dose-response data for BV2 microglial activation with LPS and IL-4, Related to Figure 5 and 6.

Figure S6. Secretome comparison of BV2 microglia in co-culture vs. carry-on monocultures, Related to Figure 7.

Figure S7. Secretome analysis of activated BV2 carry-on cultures, Related to Figure 6 and 7.

Table S1. Result overview for the different co-culture combinations of NSC and microglial cell lines.

Supplemental Experimental Procedures

Supplemental References

Figure S1

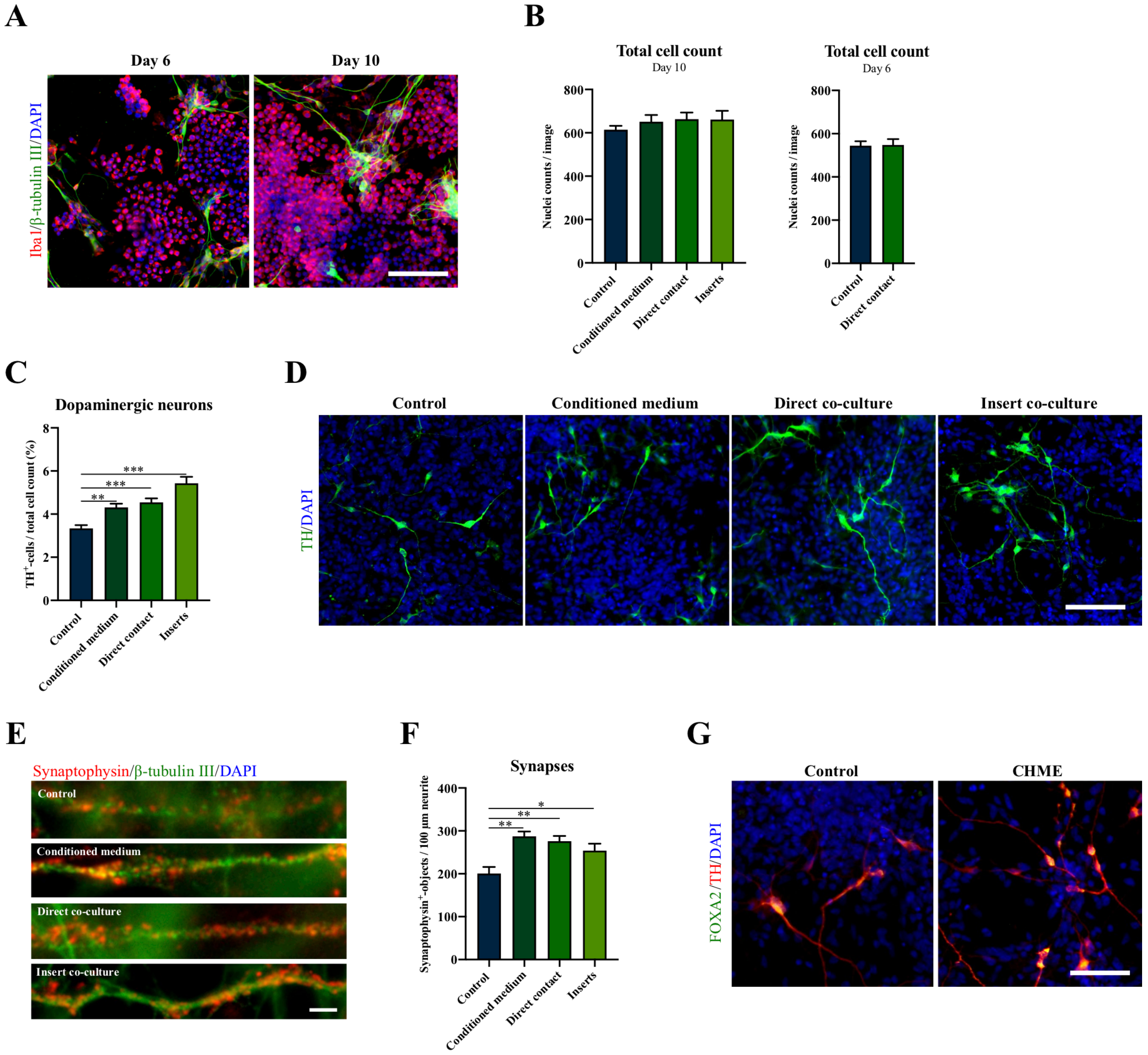

Figure S1. Additional data for the different co-culture setups, Related to Figure 1. (A) Immunofluorescence staining for the microglial marker Iba1 and the pan-neuronal marker β-tubulin III of day 6 and 10 direct co-cultures showing BV2 microglial overgrowth during the direct co-culture differentiation of hVM1-Bcl-X_L NSCs. Scalebar: 100 µm. (B) Total cell counts for the different co-culture setups with BV2 microglia and hVM1-Bcl-X_L NSCs. Day 10: control, n = 17, N= 6; conditioned medium, n = 10, N = 4; direct contact, n = 10, N = 2; inserts, n = 13, N = 4; day 6: control, n = 14, N = 4; direct contact, n = 14, N = 4. (C-D) The different co-culture setups; conditioned media, direct or indirect co-culture using membrane inserts, tested on the iPSC-NSC line XCL1 and the human CHME microglia cell line showing (C) TH^+ neurons/total cell count and (D) representative images of TH^+ neurons. Control, n = 9, N = 2; conditioned medium, direct contact and inserts, n = 6, N = 2. (E-F) Synaptogenesis, quantified as synaptophysin+ objects/100 µm neurite, in the different co-culture setups of iPSC-NSC/CHME. Scalebar: 5 µm. All groups, n = 4, N = 2. (G) Expression of the floorplate marker FOXA2 in TH^+ neurons in indirect iPSC-NSC/CHME co-cultures. Scale bar: 100 µm. One-way ANOVA, Dunnett's multiple comparison test with reference to control. Mean ± SEM. *($p < 0.05$), **($p < 0.01$), ***($p < 0.001$).

Figure S2

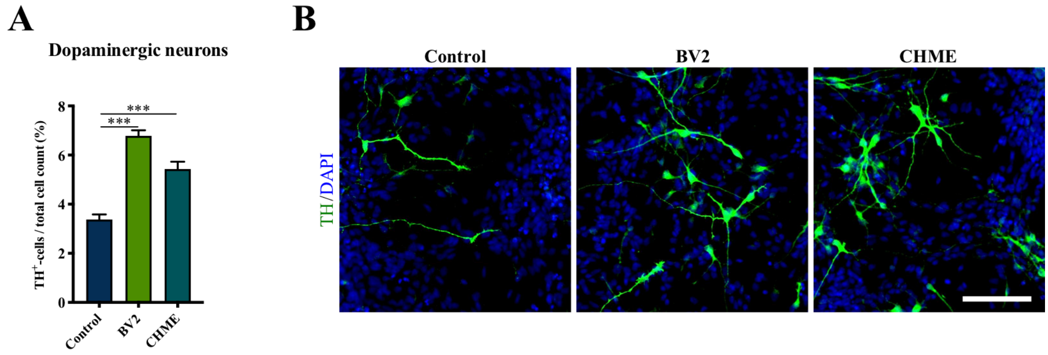

Figure S2. Consistent positive effect of BV2 and CHME microglia on dopaminergic differentiation of the iPSC-NSC line XCL-1, Related to Figure 3. (A) TH^+ neurons/total cell count and (B) representative images of TH^+ neurons. Scalebar: 100 µm. One-way ANOVA, Dunnett's multiple comparison test with reference to control. All groups, $n = 6$, $N = 2$. Mean ± SEM. ***($p < 0.001$).

Figure S3

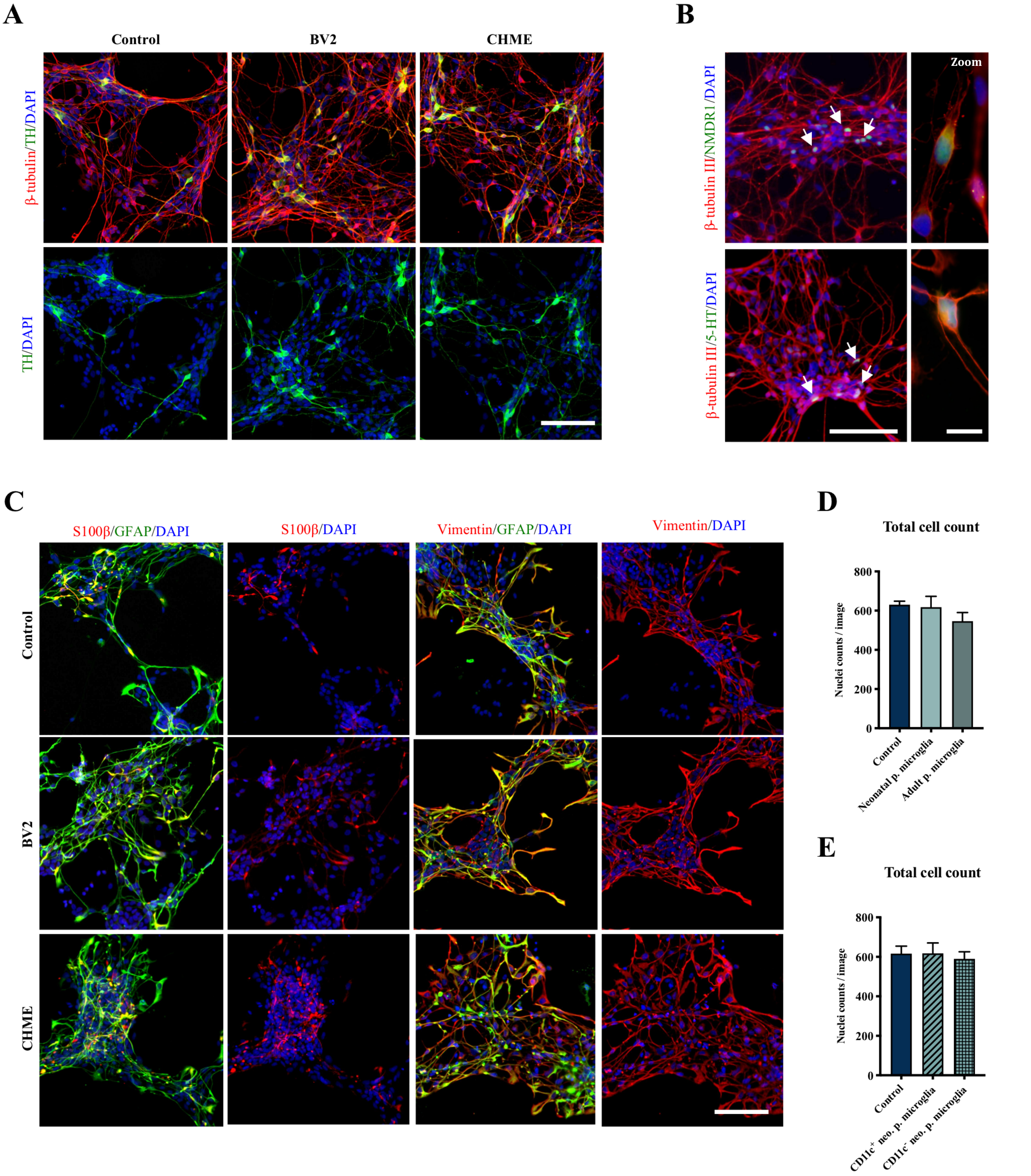

Figure S3. Further culture characterization of differentiated hVM1-Bcl-X_L/BV2/CHME co-cultures and effects on total cell count for co-culture with different primary microglial cell types, Related to Figure 3. (A) Immunofluorescence staining for TH and β-tubulin III of hVM1-Bcl-X_L NSCs differentiated in co-culture with BV2 or CHME microglia. Scalebar: 100 µm. (B) The differentiated hVM1-Bcl-X_L co-cultures contained very few glutaminergic (NMDR1$^+$) and serotonergic (5-HT$^+$) cell clusters (>1%). Scalebar: 100 µm. Scalebar zoomed image: 10 µm. (C) GFAP$^+$ astrocytes in the differentiated hVM1-Bcl-X_L co-cultures co-expressed S100β and Vimentin. Scalebar: 100 µm. (D-E) Total cell count of hVM1-Bcl-X_L cells differentiated in co-culture with (D) unsorted neonatal and adult primary microglia; control, n = 11, N = 4; neonatal p. microglia, n = 9, N = 2; adult p. microglia, n = 12, N = 4, or (E) neonatal primary

microglia sorted for CD11c; all groups, n = 8, N = 4. One-way ANOVA, Dunnett's multiple comparison test with reference to control. Mean ± SEM.

Figure S4:

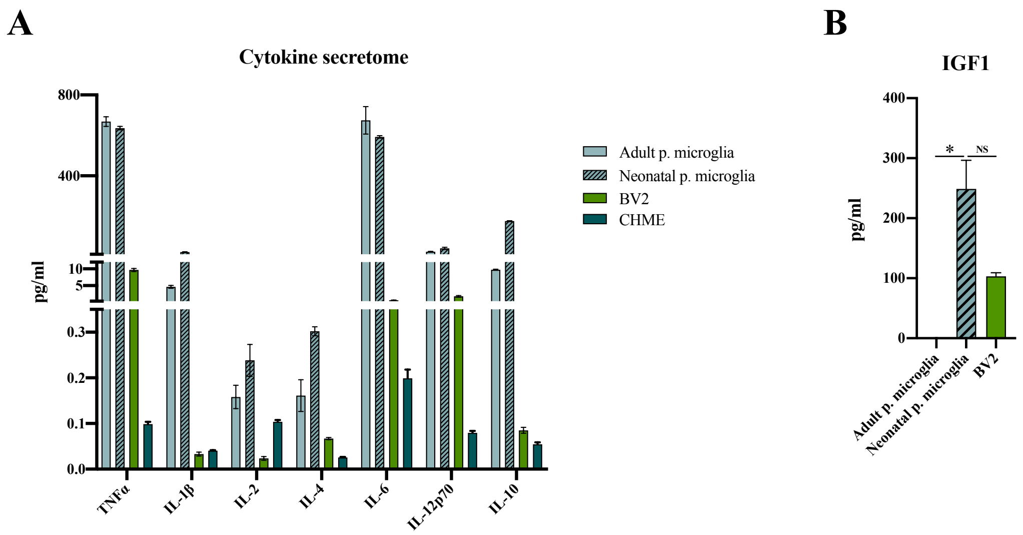

Figure S4. Secretome comparison between BV2, CHME, adult and neonatal primary microglia, Related to Figure 3. (A) Cytokine profiling of medium from adult and neonatal primary microglia, BV2 and CHME microglia. Cytokines were detected for all types of microglia, but higher concentrations were seen for primary microglia (statistical differences not indicated in the figure). All groups, n = 4, N = 2. (B) ELISA for IGF1 of medium from adult and neonatal primary microglia and BV2. All groups, n = 2, N = 2. Values are adjusted for microglia cell density. One-way ANOVA, Tukey's multiple comparison test. Mean ± SEM. *($p < 0.05$), NS = not significant.

Figure S5

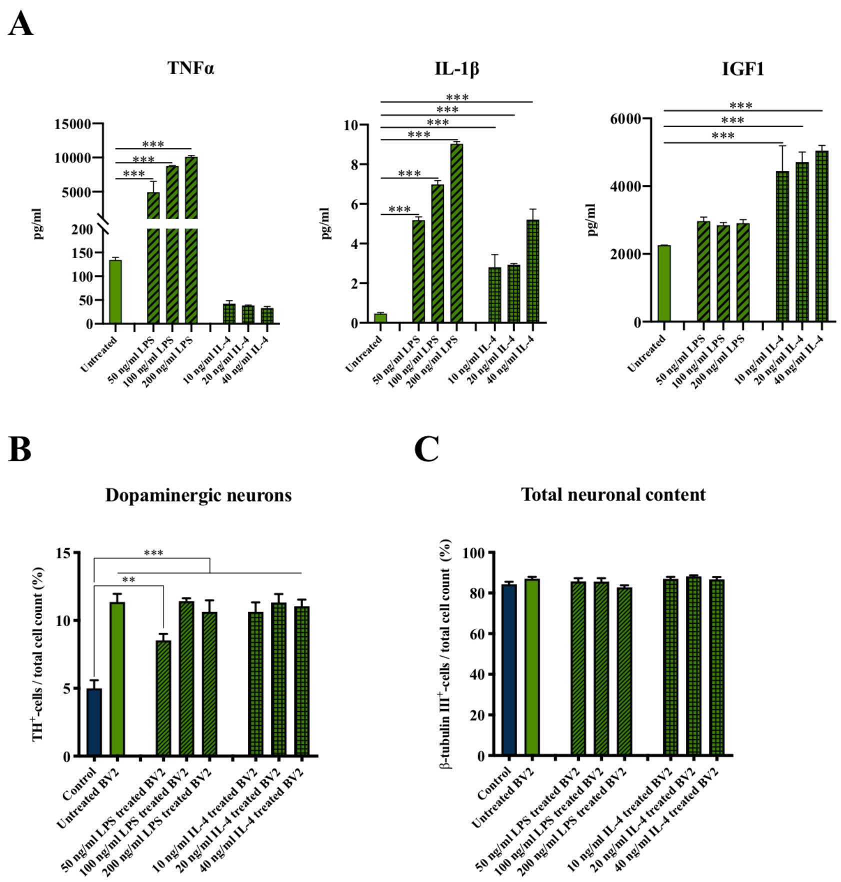

Figure S5. Dose-response data for BV2 microglial activation with LPS and IL-4, Related to Figure 6 and 7. (A) Secretome analysis of selected factors (TNFα, IL-1β and IGF1) in BV2 microglia medium 24 hours post activation with LPS or IL-4. LPS concentrations tested; 50, 100 and 200 ng/ml, IL-4 concentrations tested; 10, 20 and 40 ng/ml. All groups, n = 2-3, N = 2. (B-C) Differentiation outcome of hVM1-Bcl-X_L NSCs in co-culture with activated BV2 microglia evaluated by (B) TH^+ neurons/total cell count and (C) β-tubulin III^+ neurons/total cell count. One-way ANOVA, Dunnett's multiple comparison test with reference to control. All groups, n = 6, N = 2. Mean ± SEM. **($p < 0.01$), ***(p < 0.001).

Figure S6

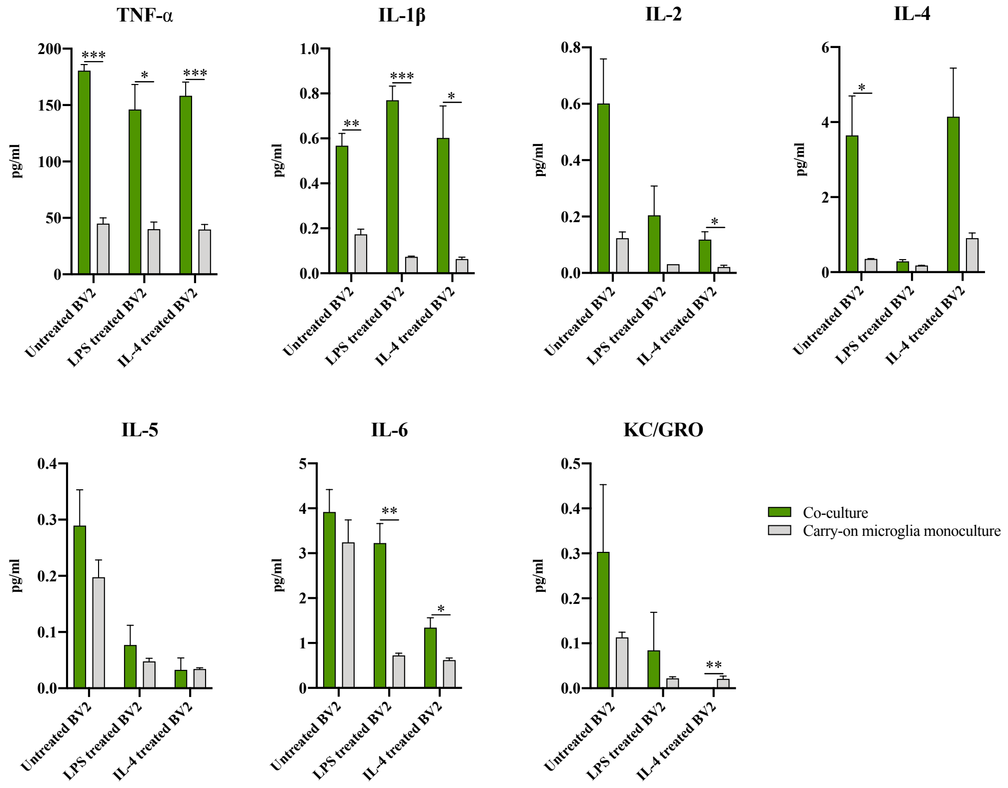

Figure S6. Secretome comparison of BV2 microglia in co-culture vs. carry-on monocultures, Related to Figure 7. Cytokine profiling of medium from day 3 co-cultures compared to day 3 carry-on BV2 monocultures showing a general increased cytokine secretion from co-cultures. Multiple t-test, Holm-Sidak's multiple comparison test. All co-culture groups, $n = 4$, $N = 2$; all carry-on groups, $n = 3$, $N = 2$. Values are adjusted for microglia cell density. Mean ± SEM. *($p < 0.05$), **($p < 0.01$), ***($p < 0.001$).

Figure S7

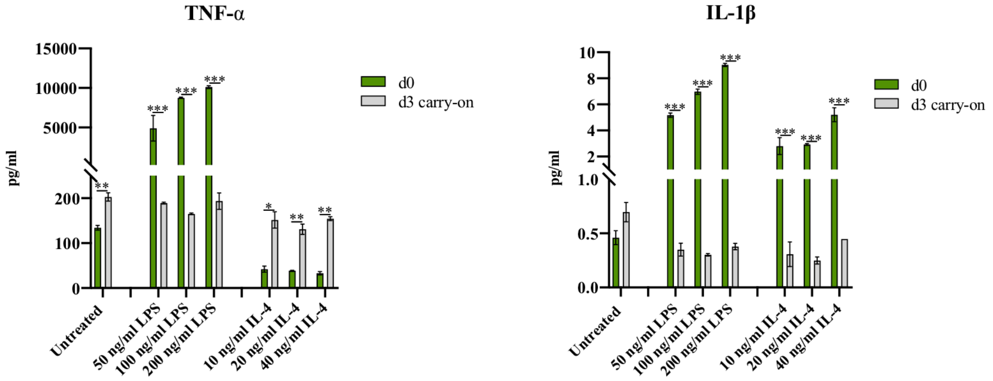

Figure S7. Secretome analysis of activated BV2 carry-on cultures, Related to Figure 6 and 7. Cytokine profiling of medium from day 3 carry-on microglia cultures activated with increasing concentrations of LPS and IL-4 for 24 hours at day 0 (selected cytokines presented; TNFα and IL-1β). Multiple t-test, Holm-Sidak's multiple comparison test. All groups, n = 2-3, N = 2. Mean ± SEM. **($p < 0.01$), ***($p < 0.001$).

Table S1: Result overview for the different co-culture combinations of NSC and microglial cell lines.

**Cell lines: NSCs + microglia**	**hVM1-Bcl-X_L + BV2**	**hVM1-Bcl-X_L + CHME**	**hVM1-Bcl-X_L + neonatal primary microglia**	**hVM1-Bcl-X_L + adult primary microglia**	**hNS1 + BV2**	**iPSC-NSC + BV2**	**iPSC-NSC + CHME**
TH$^+$ neurons/total cell count	↑*	↑*	↑*	↔	↑*	↑*	↑*
Total cell count	↔	↔	↔	↔	↑*	↑*	-
TH$^+$ neurons/β-tubulin III$^+$ neurons	↑*	↑*	↑*	↔	-	-	-
β-tubulin III$^+$ neurons/total cell count	↔	↔	↔/↑	↔	↑*	-	-
GABA$^+$ neurons/ total cell count	↓	↓*	-	-	-	-	-
GFAP$^+$ astrocytes/ total cell count	↑	↑*	-	-	-	-	-

Abbreviations: NSCs, neural stem cells; TH, tyrosine hydroxylase; GFAP, glial fibrillary acidic protein. Arrows mark whether relative cell counts are increased (↑), decreased (↓) or unchanged (↔). Significant changes are marked with *.

Supplemental Experimental Procedures

Ethics

Human cell lines were used in accordance with Danish national regulations, the ethical guidelines issued by the Network of European CNS Transplantation and Restoration (NECTAR), and the International Society for Stem Cell Research (ISSCR). The Research Ethics Committee of the Region of Southern Denmark approved the study prior to initiation (S-20130101). Ethic statements about the human fetal origin of the NSC lines hVM1-Bcl-X_L and hNS1 can be found in the original paper describing the hVM1-Bcl-X_L cell line (Villa et al., 2009).
All animal experiments were approved by the Danish Animal Experiments Inspectorate (approval number 2014-15-0201-00369).

Propagation and differentiation of NSCs

The NSC lines hVM1-Bcl-X_L and hNS1 were propagated in poly-L-lysine (PLL, 10 µg/ml, Sigma)-coated flasks in HNSC.100 medium consisting of DMEM/F12 with Glutamax (Gibco), 0.6% D-glucose (Sigma), 0.5% 1 M Hepes (Gibco), 0.5% AlbuMAX-I (Gibco), 1% N2 supplement (Gibco), 1% NEAA (Sigma), and 1% Penicillin/streptomycin (Gibco), supplemented with 20 ng/ml recombinant human epidermal growth factor (rh-EGF, R&D Systems) and 20 ng/ml recombinant human basic fibroblast growth factor (rh-bFGF, R&D Systems). Medium change was performed every third day and cells were passaged when 80-90% confluent, by dissociating the cells for 5 min with trypsin-EDTA (Gibco). For differentiation, hVM1-Bcl-X_L cells were seeded onto PLL-coated plates at a density of 50,000 cells/cm^2 and spontaneously differentiated to neurons for 10 days by withdrawal of growth factors (rh-EGF and rh-bFGF) from the HNSC.100 medium. A 50% medium change was performed every third day. The hNS1 cells were seeded onto PLL-coated plates at a density of 50,000 cells/cm^2 and differentiated for 14 days using the CK4 protocol (Krabbe et al., 2009); HNSC.100 medium supplemented with 50 ng/ml recombinant human fibroblast growth factor 8 (rh-FGF8, R&D Systems) for the first three days, followed by HNSC.100 medium supplemented with 25 µM forskolin (Sigma), 5 ng/ml recombinant human glial cell line-derived neurotrophic factor (rh-GDNF, R&D Systems), and 25 ng/ml recombinant human sonic hedgehog (R&D Systems) for the remaining 11 days with a 50% medium change every third day.
The iPSC-derived NSC line XCL1 was propagated on Geltrex (Gibco)-coated plates in Neurobasal medium supplemented with 1x B27 (Gibco), 2 mM NEAA (Gibco), 2 mM GlutaMAX-1 (Gibco), 1% penicillin/streptomycin (Gibco) and 10 ng/ml rh-bFGF (R&D Systems,). Medium change was performed every other day and cells were passaged 1:3 when 80-90% confluent, by dissociating the cells for 5 min with accutase (Gibco). For differentiation iPSC-derived NSCs were seeded onto plates coated with 20 µg/ml poly-L-ornithine (PLO, Sigma) and 10 µg/ml laminin (Life Technologies) at a density of 50,000 cells/cm^2. The first 10 days of differentiation were carried out in DOPA Induction Medium (XCell Science) supplemented with DOPA Induction Supplement A, B and C (XCell Science) and 200 ng/ml rh-SHH (Peprotech) with 50% medium change every other day. At days 5 and 10, cells were passaged using accutase for 5 min and plated at a density of 50,000 cells/cm^2 unless otherwise specified. At day 10, the medium was changed to DOPA Maturation Medium (XCell Science) with DOPA Maturation Supplement A (XCell Science) until day 16 and DOPA Maturation Supplement B (XCell Science) until day 25 with 50% medium change every other day.
NSC cultures were propagated in an incubator at 37°C with 5% CO2 and 95% humidified air containing 20% O_2 and differentiated at either the same conditions or at low O_2 tension (5% CO_2, 92% N_2, and 3% O_2) monitored by an O_2-sensitive alarm system (Forma Scientific Inc., OH, USA).

Propagation of microglial cells

BV2 and CHME microglial cell lines were propagated in PLO (15 µg/ml, Sigma)-coated flasks in RPMI-1640 with Glutamax (Gibco) supplemented with 5% heat inactivated Fetal Bovine Serum (FBS, Gibco) and 1% penicillin/streptomycin (Gibco). The culture medium was changed every second to third day and passaged 1:20 when 80-90% confluent by scraping off cells using a sterile cell scraper.
Neonatal and adult primary microglia were cultured similar to the BV2 and CHME cells (cell isolation is described below).

Mice

C57BL/6j female mice aged 7–8 weeks were obtained from Taconic Europe A/S and maintained as a breeding colony in the Biomedical Laboratory, University of Southern Denmark (Odense). Neonatal C57BL/6j mice (P3-5) and adult mice (8 weeks) used for experiments were of mixed sex.

Primary microglia isolation

The mice were anesthetized with 200 mg/kg pentobarbital and intracardially perfused with ice-cold PBS and brain and spinal cord tissues were collected. For isolating total unfractioned microglia from neonatal and adult mice, tissues were

dissociated using Neural Tissue Dissociation Kit (P) (Miltenyi Biotec) and single cells were collected after centrifugation in 37% Percoll (GE Healthcare Biosciences AB). Microglia were isolated by magnetic separation using CD11b (Microglia) MicroBeads (Miltenyi Biotec). All steps were done according to manufacturer's protocols.

For isolating CD11c$^+$ and CD11c$^-$ neonatal microglia, a single-cell suspension was generated by forcing the tissue through a 70 mm cell strainer (BD Biosciences) and single cells were collected after centrifugation in 37% Percoll. They were first incubated with anti-CD45 (Clone 30-F11; Biolegend), anti-CD11b (Clone M1/70; Biolegend), and biotin conjugated anti-CD11c (Clone HL3; BD Pharmingen) antibodies in PBS with 2% FBS and finally with streptavidin-APC (Biolegend). Cell populations were gated based on isotype-matched control antibodies as CD45dim CD11b$^+$ CD11c$^-$ (CD11c$^-$ microglia), CD45dim CD11b$^+$ CD11c$^+$ (CD11c$^+$ microglia) and sorted on a FACSAriaTM III cell sorter (BD Biosciences).

Immunocytochemistry

Cells were fixed in 4% paraformaldehyde (PFA, Sigma) in 0.15 M phosphate buffer (pH 7.4) for 20 min and washed for 3x15 min in 0.05 M Tris-buffered saline (TBS, pH 7.4) with 0.1% Triton X-100 (Sigma). For DAB staining cells were pre-incubated in TBS containing 10% FBS (Sigma) for 30 min to block unspecific antibody binding before incubation overnight (ON) at 4°C with primary antibodies diluted in TBS/10% FBS serum. Cells were washed 3x15 min in TBS/0.1% Triton X-100 and incubated for 1 hour at room temperature (RT) with biotinylated secondary donkey anti-rabbit IgG (GE Healthcare), sheep anti-mouse IgG (GE Healthcare), or goat anti-rat IgG (Vector Laboratories) diluted 1:200 in TBS/10% FBS serum. After rinsing in TBS/1% Triton-X-100, cells were incubated for 1 hour at RT in horseradish peroxidase (HRP) conjugated streptavidin (GE Healthcare) diluted 1:200 in TBS/10% FBS serum. Cells were then washed in TBS before visualization of the immunocytochemical complexes with 0.01% 3.3'-diaminobenzidine (DAB, Sigma) and 0.015% H_2O_2 (Merck) in TBS followed by mounting using glass coverslips and Aquatex (Merck #108562).

For immunofluorescence staining cells were incubated ON with primary antibodies and washed in TBS/0,1% Triton X-100 as described above. Cells were then incubated with secondary Alexa Fluor 555 goat anti-mouse IgG (Molecular Probes) and/or Alexa Fluor 488 goat anti-rabbit IgG (Invitrogen) diluted 1:500 in TBS/10% FBS serum for 2 hours at RT. Cell nuclei were counterstained with 10 μM 4'.6-diamidino-2-phenylindole (DAPI) (Sigma) for 15 min at RT. Cultures were mounted onto glass slides using ProLong® Diamond (Molecular Probes).

Primary antibodies used: mouse anti-human nuclei (HN, Merck #MAB1281) 1:500, rabbit anti-tyrosine hydroxylase (TH, Merck #AB152) 1:600, mouse anti-TH (Merck #MAB5280) 1:2000, mouse anti-β-tubulin III (Sigma #T8660) 1:2000, rabbit anti-β-tubulin III (Sigma #T2200) 1:2000, mouse anti-synaptophysin (Sigma #S5768) 1: 200, goat anti-FOXA2 (R&D, #AF2400) 1:250, rabbit anti-Iba1 (Wako #019-19741) 1:300, rabbit anti-GABA (Sigma #A2052) 1:2000, rabbit anti-NMDAR-1 (Millipore, #AB9864) 1: 100, mouse anti-5-HT (DAKO #M0758) 1:1000, rabbit anti-glial fibrillary acidic protein (GFAP, DAKO #Z0334) 1:4000, mouse anti-S100β (Sigma S2532) 1: 1000, mouse anti-vimentin (Santa Cruz #SC373717) 1:200, mouse anti-microtubule-associated protein 2a+b (MAP2, Sigma #M1406) 1:2000, and mouse anti-ki67 (BD Pharmingen #550609) 1:500, rabbit anti-cleaved caspase 3 (Cell Signaling #9661) 1: 400.

Bioimaging and image analysis

Cell counts on 3.3'-diaminobenzidine (DAB) stained cultures were performed by bright-field microscopy (Olympus) in 16 randomly selected areas per well using an ocular grid (Olympus) and normalized to total cell numbers as quantified from HN counts.

Fluorescence images were acquired either on a fluorescence microscope (Olympus) for five randomly chosen areas per coverslip or using an ImageXpress automated imaging system (Molecular Device). Cell counts were performed using the Cell Counter plugin for ImageJ and normalized to the total cell numbers as quantified by CellProfiler analysis for DAPI$^+$ nuclei (Carpenter et al., 2006; Schneider et al., 2012). Synaptophysin$^+$ objects were quantified automatically in ImageJ and normalized to neurite length. Morphological analysis was performed on hNS1-derived TH$^+$ neurons using the NeuronJ plugin for ImageJ (Meijering et al., 2004).

Western blotting

Cell pellets were lysed in phosphate buffered saline (PBS) with 1% Triton-X-100 and protease inhibitor (Complete Tablets, Roche) and sonicated for 3x10 sec at amplitude 2 microns on ice. Protein concentrations were measured with bicinchoninic acid assay (BCA, Pierce) and equal amounts of protein from each sample were denatured for 10 min at 70ºC in PBS. Proteins (10 μg) were separated on 4-12% Bis-Tris gels (NuPAGE) at 200V for 50 min with MOPS running buffer (NuPAGE) supplemented with 0.25% antioxidant (NuPAGE) and transferred to a polyvinylidene difluoride (PVDF) membranes (Invitrogen) at 20V for 8 min using the iBlot transfer system (Invitrogen). Plus2 Prestained Protein Standard (SeeBlue) was used to estimate the molecular weight of the proteins. Membranes were blocked for 60 min at 4°C in 5% skim milk (Natur Drogeriet) diluted in TBS/0.05% Tween-20 before incubation ON at 4°C with primary antibodies diluted in TBS/0.05% Tween-20. After repeated washing in TBS/0.05% Tween-20, membranes were incubated for 1 hour at RT with HRP-conjugated secondary rabbit anti-mouse IgG (DAKO) diluted 1:2000 in TBS/0.05% Tween-

20. Subsequently, membranes were repeatedly washed in TBS/0.05% Tween-20 and developed using luminol-based enhanced chemiluminescence (ECL kit, ThermoFisher Scientific) on a ChemiDoc MP imaging system (Bio-Rad). As loading control, all blots were subsequently incubated ON at 4°C with mouse anti-α actin antibody (Merck) diluted 1:6000 in TBS/0.05% Tween-20 and developed as described above.

RNA isolation, cDNA synthesis, and quantitative real-time polymerase chain reaction

For mRNA extraction, co-culture and control differentiated hVM1-Bcl-X_L cells were harvested in Trizol lysis reagent (Life Technologies) at day 10 of differentiation and lysed by vortexing for 30 sec. The RNA was purified using the RNeasy Mini kit (Qiagen) and treated with DNaseI kit according to the manufacturer's instruction. cDNA was synthesized from 250 ng of total RNA using the High Capacity cDNA Archive Kit (Applied Biosystems, Thermo Fischer). Real-time PCR of selected genes was performed using TaqMan-probe assays in an AB fast-7900HT System (Applied Biosystems, Thermo Fisher) under standard running conditions. Predesigned TaqMan assays are listed in Supplemental Information. Three replicates were run for each combination of genes and samples. Ct assignment was performed using the Sequence Detection System 2.4 software (Applied Biosystems) to set baseline and threshold parameters. Results were analyzed using the comparative method (2^{-ddCt}) and normalized to endogenous expression of *18S*, *GAPDH*, and *HPRT*.

TaqMan qPCR primers:

Target	Assay ID
VMAT2	Hs00161858_m1
DAT	Hs00168988_m1
AADC	Hs01105042_m1
PITX3	Hs00374504_m1
EN1	Hs00154977_m1
LMX1A	Hs00602600_m1
GAPDH	Hs02758991_g1
HPRT	Hs02800695_m1
18S	Hs03003631_g1

Cell death measurements

Nuclear morphological analysis was performed at day 5, 10, and 25 during differentiation of iPSC-derived NSCs. The number of fragmented $DAPI^+$-nuclei was used to estimate apoptotic cell death, and the number of pyknotic $DAPI^+$-nuclei was used to estimate necrotic cell death in the cultures (Dindler et al., 2018). Nuclear counting was performed in a blinded manner and normalized to total nuclei count.

Necrotic cell death was additionally estimated from lactate dehydrogenase release in iPSC-derived neural cultures during differentiation (day 5, 10 and 25) using the CytoTox96® Non-Radioactive Cytotoxicity Assay (Promega). The assay was performed according to the manufacturer's protocol.

LPS and IL-4 dose-response activation

BV2 microglia were activated with increasing concentrations of LPS (from Escherichia coli O111:B4, Sigma) and IL-4 (Peprotech) 24 hours prior to co-culture setup with hVM1-Bcl-X_L NSCs. Concentrations applied: LPS; 50, 100 and 200 ng/ml, IL-4; 10, 20 and 40 ng/ml.

TNFα, IL-1β and IGF1 treatment

The hVM1-Bcl-X_L NSC line were spontaneously differentiated for 10 days with addition of TNFα, IL-1β or IGF1 at day 0 in a dose-response setup. 50% media change were performed every third day. Five concentrations were tested for each factor; three concentrations matching the detected levels in the conditioned co-culture medium and two concentrations matching what has previously been used in the literature (Doherty, 2007; Ling et al., 1998; Supeno et al., 2013): TNFα; 100 pg/ml, 200 pg/ml, 400 pg/ml, 10 ng/ml and 20 ng/ml, IL-1β; 0.5 pg/ml, 1 pg/ml, 2 pg/ml, 100 pg/ml and 200 pg/ml, IGF1; 1 ng/ml, 6 ng/ml, 12 ng/ml, 100 ng/ml and 200 ng/ml.

The iPSC-NSC line XCL1 was differentiated for 25 days using the DOPA differentiation kit (XCell Science) with exposure to either 10 ng/ml TNFα, 100 pg/ml IL-1β or 100 ng/ml IGF1 during the first five days of differentiation.

Graphical illustrations:

Illustrations were made using BioRender and PowerPoint.

Supplemental References

Carpenter, A.E., Jones, T.R., Lamprecht, M.R., Clarke, C., Kang, I.H., Friman, O., Guertin, D.A., Chang, J.H., Lindquist, R.A., Moffat, J.*, et al.* (2006). CellProfiler: image analysis software for identifying and quantifying cell phenotypes. Genome Biol *7*, R100.

Dindler, A., Blaabjerg, M., Kamand, M., Bogetofte, H., and Meyer, M. (2018). Activation of Group II Metabotropic Glutamate Receptors Increases Proliferation but does not Influence Neuronal Differentiation of a Human Neural Stem Cell Line. Basic Clin Pharmacol Toxicol *122*, 367-372.

Doherty, G.H. (2007). Developmental switch in the effects of TNFalpha on ventral midbrain dopaminergic neurons. Neurosci Res *57*, 296-305.

Krabbe, C., Courtois, E., Jensen, P., Jorgensen, J.R., Zimmer, J., Martinez-Serrano, A., and Meyer, M. (2009). Enhanced dopaminergic differentiation of human neural stem cells by synergistic effect of Bcl-xL and reduced oxygen tension. J Neurochem *110*, 1908-1920.

Ling, Z.D., Potter, E.D., Lipton, J.W., and Carvey, P.M. (1998). Differentiation of mesencephalic progenitor cells into dopaminergic neurons by cytokines. Exp Neurol *149*, 411-423.

Meijering, E., Jacob, M., Sarria, J.C., Steiner, P., Hirling, H., and Unser, M. (2004). Design and validation of a tool for neurite tracing and analysis in fluorescence microscopy images. Cytometry A *58*, 167-176.

Schneider, C.A., Rasband, W.S., and Eliceiri, K.W. (2012). NIH Image to ImageJ: 25 years of image analysis. Nat Methods *9*, 671-675.

Supeno, N.E., Pati, S., Hadi, R.A., Ghani, A.R., Mustafa, Z., Abdullah, J.M., Idris, F.M., Han, X., and Jaafar, H. (2013). IGF-1 acts as controlling switch for long-term proliferation and maintenance of EGF/FGF-responsive striatal neural stem cells. Int J Med Sci *10*, 522-531.

Villa, A., Liste, I., Courtois, E.T., Seiz, E.G., Ramos, M., Meyer, M., Juliusson, B., Kusk, P., and Martinez-Serrano, A. (2009). Generation and properties of a new human ventral mesencephalic neural stem cell line. Exp Cell Res *315*, 1860-1874.
